# Simplified homology-assisted CRISPR for gene editing in *Drosophila*

**DOI:** 10.1093/g3journal/jkad277

**Published:** 2023-12-07

**Authors:** Anne E Rankin, Elizabeth Fox, Townley Chisholm, Nicole Lantz, Arjun Rajan, William Phillips, Elizabeth Griffin, Jaekeb Harper, Christopher Suhr, Max Tan, Jason Wang, Alana Yang, Ella S Kim, Naa Kwama A Ankrah, Praachi Chakraborty, Alistair C K Lam, Madeleine E Laws, Jackson Lee, Kyle K Park, Emily Wesel, Peter H Covert, Lutz Kockel, Sangbin Park, Seung K Kim

**Affiliations:** Phillips Exeter Academy, Exeter, NH 03833, USA; The Lawrenceville School, Lawrenceville, NJ 08648, USA; Phillips Exeter Academy, Exeter, NH 03833, USA; The Lawrenceville School, Lawrenceville, NJ 08648, USA; Phillips Exeter Academy, Exeter, NH 03833, USA; The Lawrenceville School, Lawrenceville, NJ 08648, USA; Phillips Exeter Academy, Exeter, NH 03833, USA; Phillips Exeter Academy, Exeter, NH 03833, USA; Phillips Exeter Academy, Exeter, NH 03833, USA; Phillips Exeter Academy, Exeter, NH 03833, USA; Phillips Exeter Academy, Exeter, NH 03833, USA; Phillips Exeter Academy, Exeter, NH 03833, USA; Phillips Exeter Academy, Exeter, NH 03833, USA; The Lawrenceville School, Lawrenceville, NJ 08648, USA; The Lawrenceville School, Lawrenceville, NJ 08648, USA; The Lawrenceville School, Lawrenceville, NJ 08648, USA; The Lawrenceville School, Lawrenceville, NJ 08648, USA; The Lawrenceville School, Lawrenceville, NJ 08648, USA; The Lawrenceville School, Lawrenceville, NJ 08648, USA; Stanford University, Stanford, CA 94305, USA; Stanford University, Stanford, CA 94305, USA; Department of Developmental Biology, Stanford University School of Medicine, Stanford, CA 94305, USA; Department of Developmental Biology, Stanford University School of Medicine, Stanford, CA 94305, USA; Department of Developmental Biology, Stanford University School of Medicine, Stanford, CA 94305, USA; Department of Medicine, Stanford University School of Medicine, Stanford, CA 94305, USA; Department of Pediatrics, Stanford University School of Medicine, Stanford, CA 94305, USA; Stanford Diabetes Research Center, Stanford, CA 94305, USA

**Keywords:** *Drosophila*, LexA, HACKy, CRISPR/Cas9

## Abstract

In vivo genome editing with clustered regularly interspaced short palindromic repeats (CRISPR)/Cas9 generates powerful tools to study gene regulation and function. We revised the homology-assisted CRISPR knock-in method to convert Drosophila *GAL4* lines to *LexA* lines using a new universal knock-in donor strain. A balancer chromosome–linked donor strain with both body color (*yellow*) and eye red fluorescent protein (RFP) expression markers simplified the identification of LexA knock-in using light or fluorescence microscopy. A second balancer chromosome–linked donor strain readily converted the second chromosome–linked *GAL4* lines regardless of target location in the *cis*-chromosome but showed limited success for the third chromosome–linked *GAL4* lines. We observed a consistent and robust expression of the yellow transgene in progeny harboring a *LexA* knock-in at diverse genomic locations. Unexpectedly, the expression of the *3xP3-RFP* transgene in the “dual transgene” cassette was significantly increased compared with that of the original single *3xP3-RFP* transgene cassette in all tested genomic locations. Using this improved screening approach, we generated 16 novel *LexA* lines; tissue expression by the derived *LexA* and originating *GAL4* lines was similar or indistinguishable. In collaboration with 2 secondary school classes, we also established a systematic workflow to generate a collection of *LexA* lines from frequently used *GAL4* lines.

## Introduction

*Drosophila melanogaster* is a powerful organism to investigate gene function in diverse biological settings, including embryonic development and metabolism. To study genes in specific *Drosophila* organs, compartments, or cell populations, investigators have developed binary gene expression systems (Brand and Perrimon 1993; Lai and Lee 2006; Potter *et al.* 2010; Kim *et al.* 2021). These systems combine (1) cell-specific *cis*-regulatory elements that drive the expression of a transgene encoding an exogenous transcriptional activator (e.g. *GAL4*), and (2) a responder transgene whose expression is directed by the transcriptional activator. However, novel challenges in studying more complex biological contexts like intercellular or interorgan communication necessitate parallel genetic manipulations of 2, or more, independent cell populations. Multiple independent binary expression systems can be combined in a single fly to study genetic perturbations of multiple tissues simultaneously. This approach has led to the conduct of powerful epistasis experiments between different tissues (Shim *et al*. 2013), simultaneous clonal lineage analysis of multiple cell populations (Lai and Lee 2006; Bosch *et al*. 2015), a visualization of specific physical cell–cell contacts (Gordon and Scott 2009; Bosch *et al*. 2015; Macpherson *et al*. 2015), and measures of hormonal responses in target cells (Tsao *et al.* 2023).

Simultaneous use of orthogonal binary expression systems requires the generation of independent cell-specific transgenic transcriptional activators. For the *LexA*/*LexAop* binary expression system, diverse tissue-specific *LexA* activator lines have been systematically generated by cloning and linking putative enhancers to *LexA* (Pfeiffer *et al.* 2010) or by inserting *LexA*-encoding transposons near endogenous enhancers (“enhancer trapping”; Kockel *et al.* 2016, 2019; Kim *et al.* 2023). This work has enabled detailed studies of tissue-specific *LexA* expression. To expand the collection of activator lines and to exploit the thousands of extant *GAL4* lines (FlyBase) as potential targets, Lin and Potter (2016) developed homology-assisted CRISPR knock-in (HACK) to replace *GAL4* with an orthogonal transcriptional activator. Similar CRISPR/Cas9-based approaches have been successfully applied to generate *LexA* lines from existing *GAL4* lines with well-characterized tissue expression patterns (Chang *et al.* 2022; Karuparti *et al.* 2023). However, screening and identifying successful but rare CRISPR gene editing in vivo has been limited by the need to use lines with “donor” sequences at chromosomal locations proximal to *GAL4* target sequences (Lin and Potter 2016) or by relying on the target *GAL4* tissue expression patterns (Karuparti *et al.* 2023).

We postulated that a HACK donor construct located on a balancer chromosome carrying multiple inversions could alleviate the proximal and distal effects of the donor and target interactions observed in *cis*-chromosomal HACK (Lin and Potter 2016). In addition, the original HACK donor plasmids are constructed with a *3xP3-RFP* transgene cassette whose expression varies at different genomic locations (Horn *et al.* 2000); thus, identifying the conversion of *GAL4* lines at some chromosomal locations has been challenging. To enhance the efficiency of identifying CRISPR-based gene editing, we added a body-color marker transgene, *yellow*^+t7.7^, in the donor construct, so that successful gene targeting can be identified using light and fluorescence microscopy. With this new donor in the *CyO* balancer chromosome, we converted *GAL4* lines with comparable efficiencies at multiple genomic locations, establishing a universal HACK donor approach to generate novel *LexA* lines with well-characterized expression patterns.

## Materials and methods

### *Drosophila* strains

Except for the *LexA.G4HACK* (abbreviated as *LexA.G4H* hereafter) donor lines, all other *Drosophila* lines provided in Table 1, Fig. 3, and Supplementary Fig. 2 were obtained from the Bloomington Drosophila Stock Center (BDSC).

**Table 1.** Genotypes of the original *GAL4* and converted *LexA* lines and their conversion rate using v1 and v2 donors.

BDSC	GAL4 transgene or insertion	Location	Version 1 conversion % [# males RFP(+) w(+)/w(+)]	Version 2 conversion % [# males RFP(+) w(+)/w(+)]	loxP cassette–removed LexA lines
25750	P{w[+mW.hs] = GawB}elav[C155]	Chr X, 1B8	1.2% (4/∼300)	n.d.	P{w[+mW.hs] = ET-lexA::GAD.GB}elav[C155-LG]
8860	P{w[+mW.hs] = GawB}Bx[MS1096]	Chr X, 17C3	1.0% (6/∼600)	n.d.	P{w[+mW.hs] = ET-lexA::GAD.GB}Bx[MS1096-LG]
29968	P{w[+mW.hs] = GawB}Feb36	Chr 2, 22A8	0.5% (2/459)*^a^*	0.4% (12/2967)*^b^*	P{w[+mW.hs] = ET-lexA::GAD.GB}Feb36
26160	P{w[+mW.hs] = GawB}VGlut[OK371]	Chr 2, 22E1	2.8% (12/425)*^a^*	2.5% (82/3238)*^b^*	P{w[+mW.hs] = ET-lexA::GAD.GB}VGlut[OK371-LG]
	P{y[+t7.7] w[+mC] = Dilp215-1-GAL4}attP40	Chr 2, 25C6	n.d.	3.0% (22/736)	P{y[+t7.7] w[+mC] = Dilp215-1-LexA.G4H}attP40
5818	P{w[+mW.hs] = GawB}459.2	Chr 2, 32F1	3.5% (23/651)*^a^*	0.4% (3/829)*^b^*	P{w[+mW.hs] = ET-lexA::GAD.GB}459.2
25373	P{w[+mW.hs] = GawB}dimm[929] crc[929]	Chr 2, 39C2	n.d.	0.1% (2/1781)	P{w[+mW.hs] = ET-lexA::GAD.GB}dimm[929-LG] crc[929-LG]
30026	P{w[+mW.hs] = GawB}GH146	Chr 2, 51A4	0.7% (5/683)*^a^*	0.9% (10/1114)*^b^*	P{w[+mW.hs] = ET-lexA::GAD.GB}GH146
30139	P{w[+mC] = Hml-GAL4.Delta}2	Chr 2, 57D13	5.3% (42/799)*^a^*	2.4% (34/1417)*^b^*	P{w[+mC] = Hml-LexA.G4H.Delta}2
47473	P{y[+t7.7] w[+mC] = GMR16H11-GAL4}attP2	Chr 3, 68A4	n.d.	0.0% (0/543)	
33832	P{w[+mC] = r4-GAL4}3	Chr 3, 68C13	n.d.	0.8% (4/516)	P{w[+mC] = r4-LexA.G4H}3
43343	P{w[+mW.hs] = GawB}bbg[C96]	Chr 3, 70D7-70E1	n.d.	0.5% (4/784)	P{w[+mW.hs] = ET-lexA::GAD.GB}bbg[C96-LG]
8816	P{w[+mW.hs] = GawB}D42	Chr 3, 71C2	n.d.	0.5% (2/381)	P{w[+mW.hs] = ET-lexA::GAD.GB}D42
5138	P{w[+mC] = tubP-GAL4}LL7	Chr 3, 79A2	n.d.	0.1% (1/688)	P{w[+mC] = tubP-LexA.G4H}LL7
7415	P{w[+m*] = GAL4}repo	Chr 3, 90F9	n.d.	0.4% (2/534)	P{w[+m*] = ET-lexA::GAD}repo[LG]
32079	P{w[+mC] = ppk-GAL4.G}3	Chr 3, 94D3	0.4% (3/844)*^a^*	0.2% (1/489)*^b^*	P{w[+mC] = ppk-LexA.G4H.G}3

Source IDs and genotypes of *GAL4* lines selected for the gene conversions and their conversion rates by donor version; *^a^* and *^b^* indicate lines with data for both donor versions. Following the convention of FlyBase genotype nomenclatures, the converted lines from *P{GawB}*-based enhancer trap *GAL4* insertions were named as *P{ET-lexA::GAD.GB}*, and the converted lines from cloned enhancer–driven *GAL4* transgenes were named by replacing *GAL4* in the original genotypes with *LexA.G4H*. n.d., not determined.

*^a^* Converted by students using v1.

*^b^* Converted by students using v2.

### Generation of version 1 and version 2 *LexA.G4H* donor strains

The construction of pHACK-GAL4>nlsLexA::GADfl (v1) and its insertion into *PBac{y^+^-attP-9A}42A13* on the *CyO* balancer chromosome were described previously (Chang *et al.* 2022). The *CyO* balancer chromosome with the v1 donor transgene was combined with the *PBac{y+^mDint2^ GFP^E.3xP3^=vas-Cas9}VK00027* transgene on the third chromosome (BDSC 51324) to make a fully functional v1 donor strain as previously reported (Chang *et al.* 2022).

A total of 4,965-bp *y^+^*^t7.7^ fragment carrying 2,882-bp *yellow* gene enhancer and promoter and 2,038-bp *yellow* gene cDNA sequence was amplified from pCaryP (Groth *et al.* 2004) using the primers *y^+^*^t7.7^_F2 (5′-ATTAGTCTCTAATTGAATGACGTCGCATACTTACATTTTTTCCGCTTTTTCCG-3′) and *y^+^*^t7.7^_R (5′-GCTATACGAAGTTATGACGTCGTCGACTATTAAATGATTATCGCCCGATTACC-3′). The amplified transgene fragment was inserted to an AatII site between the multimerized *Pax6* responsive “3xP3” promoter (Horn *et al.* 2000) and a *loxP* site on pHACK-GAL4>nlsLexA::GADfl (v1) using the NEBuilder HiFi DNA Assembly Cloning Kit (New England BioLabs, E5520S). This resulted in the generation of a *loxP*-flanked dual transgene cassette (*loxP*-RFP-3xP3-*yellow* transgene-*yellow* enhancer-*loxP*). The resulting construct pHACKy-GAL4>nlsLexA::GADfl (v2, GenBank Accession OR687150) carrying both *3xP3-RFP* and *yellow* transgene markers was inserted into the *PBac{y^+^=attP-9A}42A13* site on the *CyO* chromosome (the same site as the v1 donor construct). The *CyO* balancer chromosome with the v2 donor transgene was combined with the *M{GFP^E.3xP3^=vas-Cas9.RFP^−^}ZH-2A* transgene on X chromosome (BDSC 55821) to make a fully functional v2 donor strain.

### Intercross strategy for CRISPR/Cas9-based conversion of *GAL4* to *LexA.G4H*

All genetic crosses were incubated at 25°C to control developmental speed and to enhance the *Curly* wing phenotype for ease of scoring. For the F0 intercross, each vial contained 4 males of the *GAL4* line and 4 virgin females of the *LexA* donor line (either v1 or v2). The F0 intercross was transferred to new vials every 3 days for 2 weeks. When F1 progeny emerged, each male progeny carrying *w^+^* and *CyO* was mated to 2 virgin females of *y^1^ w^1118^* (BDSC 6598). Although additional virgin females may produce more F2 progeny to screen, only 2 females were used per a vial to prevent overcrowded F2 progeny that may suppress the *Curly* wing phenotype. At least 20 mating pairs were set up to identify independent conversion events from different males. These F1 mating pairs were transferred to new vials once after 5 days of mating to extend the number of F2 male progeny to screen for, but we found that this may not be necessary if 40 or more mating pairs were initially set up. For the v1 HACK donor line, F2 male progeny with *w^+^* and non-*CyO* markers were selected and screened for red fluorescent protein (RFP) expression in ocelli under a fluorescence stereo microscope. For the v2 HACK donor line, we screened for males carrying *w^+^*, *y^+^*, and non-*CyO* markers under a light stereo microscope, and then confirmed their RFP expression in ocelli under a fluorescence stereo microscope. All F2 male progeny with *w^+^* and non-*CyO* markers were counted to calculate the overall conversion rates provided in Table 1. To assess the HACK-mediated gene conversion efficiency in independent male germlines, we measured the frequencies of gene conversion events from each mating pair and plotted them in Supplementary Fig. 1. *GAL4* stocks usually carry a wild-type Y chromosome, but we noted that some *GAL4* stocks harbor undocumented *Dp(1; Y)y^+^* chromosomes and could interfere with body color–based screening in F2 generation. Two independently converted males per each *GAL4* line were saved for further analysis.

### Removal of *loxP* cassette from HACK-converted *LexA.G4H* lines

A single converted F2 male was mated to 2 virgin females carrying *P{Crey}* on the X chromosome (BDSC 766). A single F3 male carrying the *w^+^* marker was mated to 2 virgin females of *y^1^ w^1118^* (BDSC 6598). A single founder F4 male with *w^+^*, but without the *y^+^* cuticle color marker or RFP expression in the ocelli, was mated to a balancer line (e.g. BDSC 59967) to isolate the chromosome carrying *LexA.G4H* with only *w^+^* marker. Even without a heat shock, all F4 males that we have seen were without RFP and *y^+^* markers, indicating the high expression of *Cre* in F3 male germlines harboring the *P{Crey}* transgene.

### PCR genotyping and sequencing of converted *LexA.G4H* lines

Genomic DNAs from the original *GAL4*, HACK donor, and converted *LexA* male flies were extracted as previously reported (Chang *et al.* 2022). One microliter of the extracted genomic DNA was added to 19 μl of PCR master mix containing 7 μl of water, 10 μl of Q5 Hot Start High-Fidelity 2× Master Mix (NEB M0494S), 1 μl of 10 μM primer 1 (5′- ATGAAGCTACTGTCTTCTATCGAACAAGC-3′) for a GAL4 sequence, and 1 μl of 10 μM primer 2 (5′- GGCATACCCGTTTGGGATATATGATCC-3′) for a HACK donor sequence. After a 30-s denaturing period at 98°C, 35 cycles of PCR amplification were performed as a 10-s denaturing period at 98°C, a 30-s annealing period at 60°C, and a 1-min extension period at 72°C. The PCR reactions from *GAL4*, donor, and converted flies were resolved in TAE-agarose gel electrophoresis. A total of 1367-bp-long PCR product was amplified only from converted flies, isolated using the Zymoclean Gel DNA Recovery Kit (Zymo Research D4008), and sequenced from both ends using primer 1 and primer 2.

### Imaging of reporter gene expression

*P{10XUAS-IVS-mCD8::GFP}attP2* (BDSC 32185) and *P{13XLexAop2-mCD8::GFP}attP2* (BDSC 32203) were used to compare the expression patterns of the original *GAL4* and converted *LexA.G4H* line pairs. Because of genomic positional effects on reporter transgene expression (Pfeiffer *et al.* 2010), we avoid using reporters (e.g. BDSC 66680 used in Chang *et al.* 2022) located in other genomic locations. Four virgin females carrying green fluorescent protein (GFP) reporters were mated to a single male of *GAL4*, *LexA.G4H* (RFP^+^), or *LexA.G4H* (RFP^−^) lines. The mating pairs were transferred to new vials every 2 days until the imaging of expression patterns had been completed. For imaging larval tissues, inverted third instar larvae at the wandering stage were fixed at 4% paraformaldehyde in PBS for >16 h at 4°C and washed 3 times in PBS containing 0.1% Triton X-100. Larval brains and imaginal discs were dissected from the washed carcass, transferred onto a glass slide, immersed in 6 μl of the mounting media with 4',6-diamidino-2-phenylindole (DAPI) (Vectashield H-1200) for 1 min, and mounted under an 18 × 18 cover glass. The images of GFP, RFP, and DAPI channels were captured on a compound fluorescence microscope and edited using ImageJ software (NIH). For the live imaging of early pupal hemocytes, third instar larvae at the wondering stage were starved on a 2% agar plate for 4 h, and circulating hemocytes in pupating larvae were imaged under a fluorescence stereo microscope for 30 s (Supplementary Movie 1).

## Results

### A simplified genetic strategy for identifying successful gene conversion in vivo

**Fig. 1.**
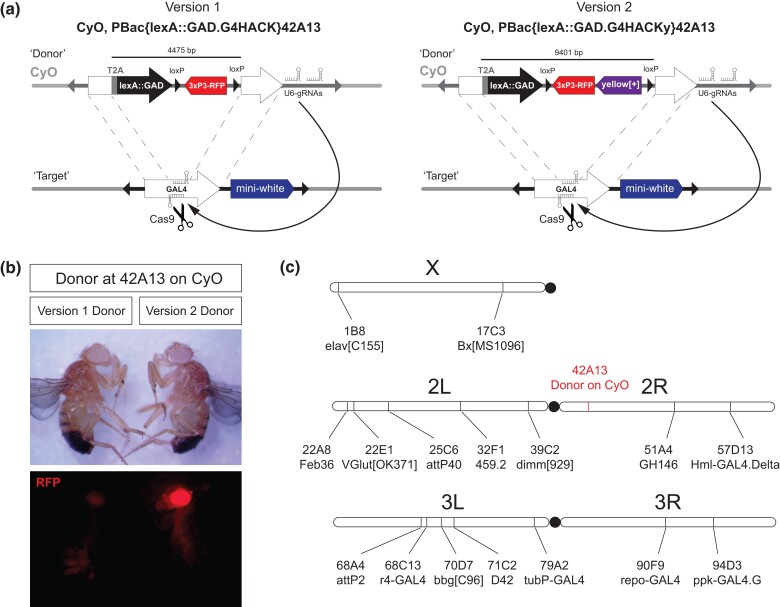
The designs of *LexA.G4HACK* donors for the CRISPR/Cas9-mediated *GAL4* gene conversion and chromosomal locations of GAL4 targets. a) The genetic designs of 2 *LexA.G4HACK* donors for HACK-mediated gene conversion. A DNA double-strand break generated by *vas-Cas9* and gRNAs targeting the *GAL4* sequence in germline chromosomes can be repaired by homology-assisted CRISPR/Cas9 knock-in of a donor transgene located in a balancer chromosome. The version 2 donor carries a *loxP*-flanked dual transgene cassette. Both versions of the donor are inserted in the same attP site on the *CyO* balancer to enable an unbiased comparison of the donor efficiency differences potentially generated by different repair template sizes. b) The version 2 donor transgene at the genomic location of 42A13 on the *CyO* balancer showed an improved *3xP3-RFP* expression compared with the version 1 donor at the same location. The *yellow*^+^ phenotypes in both flies shown are from *PBac{y^+^-attP-9A}42A13* on the *CyO* balancer. c) The chromosomal locations of selected *GAL4* targets for HACK-mediated gene conversion and the donor location.

A red fluorescent eye marker, *3xP3-RFP*, was used in the original HACK study to detect the successful editing of *GAL4* (Lin and Potter 2016, Chang *et al.* 2022; hereafter the version 1 donor or “v1”). However, the genomic positional effects of the *3xP3-RFP* expression hinder the efficient screening of rare knock-in events, thus limiting the HACK approach. To identify and verify a successful HACK gene conversion with an independent transgene marker, we produced a new transgenic donor strain harboring a 5 kb *y*^+t7.7^ transgene carrying the *yellow* gene enhancer and intron-less *yellow* coding sequence, inserted next to the *3xP3-RFP* transgene (Fig. 1a: see *Materials and methods*). Briefly, we generated a plasmid construct called pHACKy-GAL4>nlsLexA::GADfl (version 2 donor or “v2” hereafter) and inserted this in the *PBac{y+-attP-9A}42A13* genomic site on the *CyO* balancer, the same position as pHACK in v1 donors (Fig. 1a: see *Materials and methods*). Unexpectedly, adults harboring the v2 donor had enhanced RFP expression in eyes and ocelli compared with the v1 donor at the same molecular location (Fig. 1b), indicating that the 5-kb *yellow*^*+t7.7*^ transgene may have improved the expression of the neighboring *3xP3-RFP* transgene in this genomic location.

For the v1 donor experiment, the *PBac{vas-Cas9}VK00027* transgene located on the third chromosome (BDSC 51324: Port *et al.* 2015) was used (Chang *et al.* 2022). With the v2 donor, we switched to the X-linked *M{vas-Cas9.RFP^−^}ZH-2A* transgene in a *yellow* background (BDSC 55821, Port *et al.* 2015) to facilitate the screening of *yellow* transgene integration events (see *Materials and methods*). To determine whether the additional 5-kb payload in the v2 donor and the use of a different *Cas9* transgene would affect the overall HACK efficiency, we measured *GAL4* > *LexA.G4H* conversion in 6 *GAL4* lines (*^a^* and *^b^* in Table 1) using the v1 and v2 HACK donors. Overall, the HACK efficiencies of v2 were slightly lower (1.4%, *n* = 10,054) than those of the v1 donor (2.3%, *n* = 3,861). However, the relative HACK efficiencies among the different target locations appeared similar between v1 and v2 except for the 32F1 location, indicating that the v2 HACK donor is comparable with v1 in *GAL4* target–gene conversion efficiency.

To assess the frequency of gene conversion (*GAL4* to *LexA.G4H*) in the germ cell lineage of individual male flies, we measured the frequencies of conversion events stemming from individual male mating. This was contrasted with the measurement of the overall conversion rate (Table 1), which reflects data pooled from a standard-sized F1 intercross (*n* = 40); this quantification scheme differs slightly from that of a prior study (Lin and Potter 2016), which combined data from 4 males to determine conversion rates. Conversion frequency from an individual F1 male was scored (red number on each bar in Supplementary Fig. 1). In the lines with higher overall conversion rates (*OK371-GAL4* and *Hml-GAL4* in Supplementary Fig. 1), we observed that conversions were more frequent from independent males (40/94 and 17/59), with only a few male germ lines (12/94 and 3/59) producing 3 or more conversion events. Conversely, lines with lower overall conversion rates produced conversions less often from independent males (2/23 for *459.2-GAL4* and 2/37 for *dimm-GAL4*) but did not necessarily produce a smaller batch of conversion events (all 22 events found in 1/16 mating for *Ilp215-1-GAL4* at *attP40*). We conclude that the parallel screening of a relatively large number (e.g. *n* > 40) of male germlines would improve the efficiency and speed of identifying the successful targeting of genes with low-frequency conversion (see *Materials and methods*).

HACK-mediated gene conversions on second chromosome–linked *GAL4* lines (*cis*-chromosomal HACK) were all successful (*n* = 7/7), with efficiency rates averaging between 0.1% and 5.3% (Table 1). Prior studies of *cis*-chromosomal HACK found that HACK donors more proximal to *cis*-targets converted at higher efficiency than distal donors (Lin and Potter 2016). However, with a single donor location on the second balancer chromosome *CyO*, we did not observe this proximity effect on 2 homologous chromosomes. For example, using distally located (42A13) donors on the *CyO* balancer, 2 *GAL4* targets closely located at 22A8 and 22E1 show respective HACK efficiency rates of 0.4–0.5% vs 2.5–2.8%, indicating that a homology-directed repair (HDR) donor located on a balancer chromosome can be successfully used to convert distally located targets on its homologous chromosome. However, the large difference in conversion efficiency observed (5–7-fold) at the neighboring target genomic locations suggests that HACK efficiencies are likely determined by molecular locations of targets rather than the donor location on a chromosome with multiple inversions. For third chromosome–linked *GAL4* lines (*trans*-chromosomal HACK), 6/7 conversions were successful, but the average conversion efficiency rate was lower (0–0.8%: Table 1), which is in agreement with that of the prior study showing that *trans-*chromosomal HACK is possible but less efficient (Lin and Potter 2016). In sum, the v2 HACK donor on the *CyO* balancer showed comparable performance with v1 and can be used for both *cis-*chromosomal and *trans-*chromosomal HACKing of *GAL4* lines to *LexA.G4H*.

### Visible phenotypes permit efficient screening and the identification of successful HACKing

**Fig. 2.**
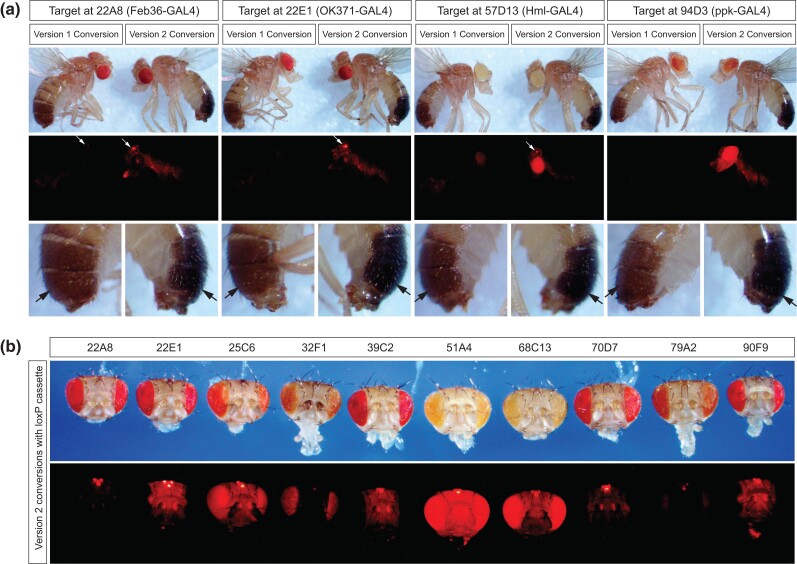
Improved RFP expression of integrated version 2 donor at various genomic locations. a) A phenotypic comparison of F2 males with successful donor integrations at different targets. RFP expression in ocelli (white arrows) was more consistently observed in version 2 integration sites than in the corresponding version 1 integration sites. The version 2 integration events can also be identified by black pigment expression in tail segments of the *y*^1^*w*^1118^ mutant genetic background (black arrows). b) The RFP expression of the integrated version 2 donor at different genomic locations. Adult heads of converted males were arranged based on target locations. RFP expression in ocelli was consistently high in all locations, but the expression in compound eyes was highly variable in different locations. Note that the expression of *mini-white* and *3xP3-RFP* was inversely correlated in compound eyes (see text).

Based on our observation of brighter RFP expression in v2 donor flies compared with v1 donors (Fig. 1b), we postulated that this difference might persist after CRISPR-based *GAL4*>*LexA.G4H* conversion. We compared the RFP expression after conversion at 4 different genomic locations (22A8, 22E1, 68C13, and 94D3). In each, the integrated v2 donor showed the bright RFP expression in ocelli (white arrows, Fig. 2a). To assess the RFP expression after CRISPR/Cas9-mediated targeting with v2 donors at diverse genomic target locations, we compared heads of 10 converted *GAL4*>*LexA.G4H* flies (Fig. 2b). After the successful conversion of all 10 lines, we observed that the RFP expression in compound eyes was variable at different loci, as previously reported (Horn *et al.* 2000), but the RFP expression in ocelli cells was observed in all integration sites. Thus, ocelli-based screening provides a reliable method for identifying v2 donor–generated conversion events with a fluorescence stereomicroscope.

**Fig. 3.**
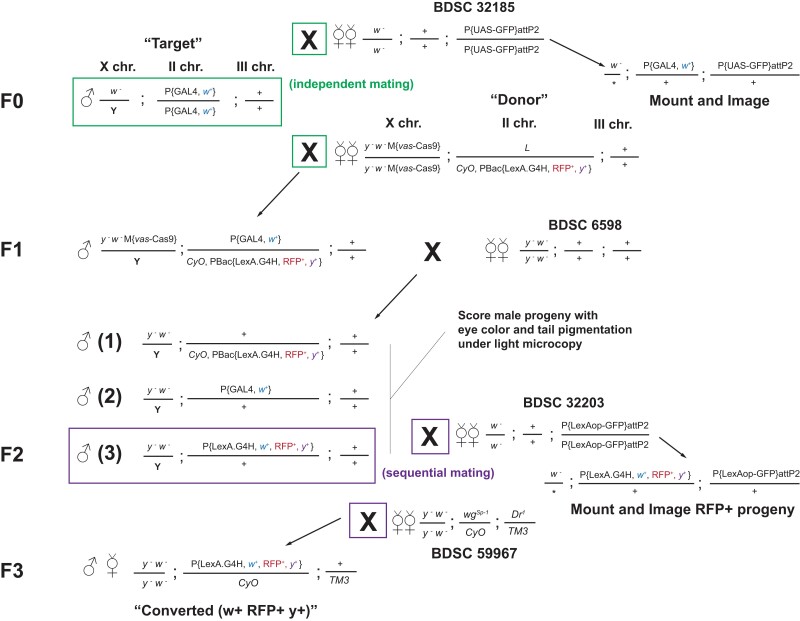
Mating scheme for converting the second chromosome–linked *GAL4* lines to *LexA.G4H* and imaging reporter expression. The parental mating (F0) was set up with a male carrying the *GAL4* transgene (“Target”) and virgin females carrying vasa-Cas9 on the X chromosome and a HACK donor on the *CyO* balancer (“Donor”). In parallel, a male carrying the same “Target” *GAL4* transgene was also mated with virgin females carrying the *UAS-GFP* transgene (BDSC 32185) for the documentation of the GFP expression pattern of the “Target” *GAL4*. These mating pairs were transferred to new vials every 2 days for 6 times. The larval, pupal, and adult progenies from *UAS-GFP* mating were imaged for GFP expression patterns based on prior characterizations of the “Target” *GAL4* line. Up to 80 individual mating pairs were set up for an F1 male progeny carrying all 3 transgenes and 2 virgin females of *y*^1^*w*^1118^ (BDSC 6598). In F2 generation, noncurly male flies carrying mini white transgene were scored for RFP expression in ocelli and/or yellow transgene expression in tail segments. If identified, a single F2 male carrying the mini-white and RFP transgenes was first mated with virgin females carrying *LexAop-GFP* transgene (BDSC 32203) for 3 days. The same male was mated again with different virgin females carrying balancer chromosomes (e.g. BDSC 59967) to isolate the chromosome with the modified transgene [“Converted (RFP^+^)”].

In addition to the RFP expression, conversion with the v2 donor also led to progeny with visibly darker-pigmented abdominal segments (black arrows in Fig. 2a), consistent with the expression of the *yellow* transgene (*y*^+t7.7^) in a *yellow* mutant (*y*^1^) genetic background. Thus, the *yellow* transgene embedded in the v2 donor sequence simplified screening for HACKy-mediated gene conversion events with bright-field microscopy (Fig. 3), followed by a confirmation of RFP expression with fluorescence microscopy. Although the v2 donor showed slightly reduced HACK efficiencies compared with v1, the improved RFP expression of v2 would make screening by fluorescence easier. Thus, we recommend designing future new HACK constructs with a *Cre*-excisable dual transgene cassette.

To establish multiple converted *LexA* lines from an independent HACK event, a single F2 male carrying RFP^+^, *y*^+^, and *w*^+^ markers was selected from independent mating pairs. We established 2 or 3 independent *LexA* conversion lines and assessed the tissue expression pattern of a *LexAop* reporter, compared with the expression of a *UAS* reporter in the original *GAL4* line (Fig. 3). All the tested *LexA* lines showed similar expression patterns to the original *GAL4* (see below), indicating that nonspecific and nontargeted random integration events of the donor sequence are rare. Once the *LexA* expression was confirmed, a newly established *LexA* line harboring the *loxP*-flanked *3xP3-RFP* and *yellow* dual transgene cassette was selected and mated to a *Cre*-expressing line to remove the dual transgene cassette (Supplementary Fig. 2; see *Materials and methods*). In summary, 2 markers in the v2 donor—*yellow* and RFP—simplified and facilitated the efficient screening of HACKy-mediated gene conversion events using light or fluorescence microscopy.

### Tissue expression patterns of originating *GAL4* and converted *LexA.G4H* lines

To test if the *LexA* expression in converted lines was identical to that in the originating *GAL4* line, we performed intercrosses to assess and compare *LexA*-dependent and *GAL4*-dependent reporter gene expressions. A single male from each original *GAL4* line was mated to virgin females carrying *10xUAS-mCD8::GFP*, and a single converted *LexA.G4H* male from each screen was mated to virgin females carrying *13xLexAop2-mCD8::GFP* (Fig. 3). To minimize the positional effects of reporter transgene expression, we used GFP reporter transgenes located at the same genomic location on the third chromosome, attP2 (Pfeiffer *et al.* 2010).

**Fig. 4.**
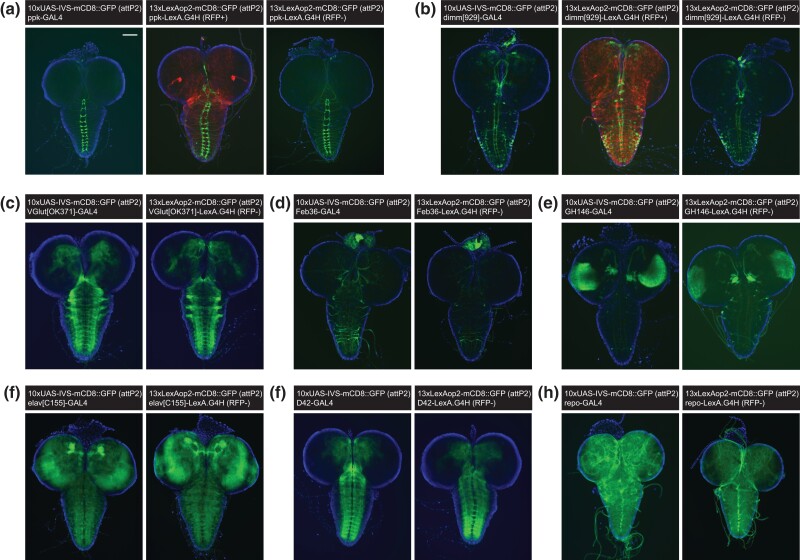
A comparison of larval brain reporter expression for the original *GAL4* and converted *LexA.G4H* lines. a) GFP reporter expression in the ventral nerve cords of larval brains driven by *ppk-GAL4* (left), *ppk-LexA.G4H* with RFP transgene (middle), and *ppk-LexA.G4H* with RFP cassette removed (right). The scale bar is 100 μm. b) GFP reporter expression in the neuroendocrine cells of larval brains driven by *dimm-GAL4* (left), *dimm-LexA.G4H* with RFP transgene (middle), and *dimm-LexA.G4H* with RFP cassette removed (right). c–h) GFP reporter expression in larval brains driven by *GAL4* (left) and *LexA.G4H* with RFP cassette removed (right) showing expression in vGlut neurons by *OK371* enhancer c), corpora cardiaca cells by *Feb36* enhancer d), brain hemispheres by *GH146* enhancer e), pan-neuronal cells by *C155* enhancer f), ventral nerve cords by *D42* enhancer g), and pan-glial cells by a cloned *repo* enhancer h).

The expression patterns of GFP in the converted *LexA* lines matched that of the original *GAL4* lines (Fig. 4a and b), an assessment that was less ambiguous after a *Cre*-mediated excision of the donor *loxP*-flanked *3xP3-RFP* and *yellow* transgene cassettes (see RFP^+^ cells in Fig. 4a and b; Supplementary Fig. 2). While this *loxP*-flanked transgene cassette did not appear to alter the expression of *LexA* lines, we removed this cassette in all the converted *LexA* lines.

**Fig. 5.**
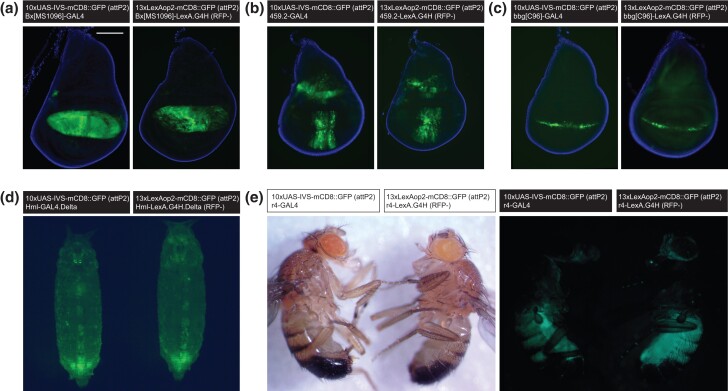
A comparison of reporter expression for the original *GAL4* and converted *LexA.G4H* lines in larval wing discs, pupal hemocytes, and adult fat bodies. a–c) GFP reporter expression in larval wing discs driven by *GAL4* (left) and *LexA.G4H* with RFP cassette removed (right) marking cells in the dorsal pouch by *MS1096* enhancer a), broad anterior-posterior boundaries by *459.2* enhancer b), and dorsal-ventral boundaries by *C96* enhancer c). The scale bar in a) is 100 μm. d) GFP reporter expression in early pupae driven by *GAL4* (left) and *LexA.G4H* with RFP cassette removed (right) showing expression in circulating hemocytes by a cloned *Hml* enhancer. The image is a still frame from a 30-s-long live imaging (Supplementary Movie 1). e) GFP reporter expression in the fat body of adult males driven by *r4-GAL4* (left side of each image) and *r4-LexA.G4H* with RFP cassette removed (right side of each image).

In the third instar larval brains of converted *LexA.G4H* lines, the GFP reporter expression patterns appeared indistinguishable from reporter expression in the original *GAL4* lines (Fig. 4). However, the intensity of the GFP signal of some converted LexA lines (Fig. 4e and h) appeared slightly reduced, compared with the reporter GFP signal in the original GAL4 lines. In the third instar wing discs, the converted *LexA* lines that drive reporter expression in the dorsal compartment of the wing disc (Fig. 5a), the entire wing disc (Fig. 5b), or the dorsoventral boundary of the wing disc (Fig. 5c) showed identical patterns to the original *GAL4* lines. In whole animal live imaging, mCD8::GFP signals on circulating hemocytes that migrate from anterior to posterior in early pupa (Supplementary Movie 1) also appeared identical between the *GAL4* and *LexA* lines (Fig. 5d). Compared with the original *GAL4* lines, converted lines expressing *LexA* in the adult abdomen and head fat body also showed similar reporter GFP expression patterns (Fig. 5e). Taken together, our analysis confirmed that the transactivation functions of converted *LexA.G4H* lines are indistinguishable from the original *GAL4* fly lines.

### Innovating secondary school curricula for the systematic generation of LexA enhancer lines

To test if the CRISPR-based Stan-X curriculum could be implemented in secondary school classes, we partnered with 2 secondary schools that had previously collaborated with us to develop relevant fruit fly-based science instruction (https://www.stan-x.org). As conversion targets, we selected the *GAL4* lines whose expression patterns were previously well characterized. We sequentially developed 2 courses for teaching fly genetics covering the CRISPR/Cas9-mediated gene conversion, larval tissue dissection, and fluorescence imaging techniques (Supplementary Fig. 3). In the first course using the v1 donor, 6 students focused on experimental design, execution, and interpretation and successfully converted assigned GAL4 lines (*^a^* in Table 1) over a 10.5-week schedule (Supplementary Fig. 3a). Students performed intercrosses and screened for a “HACKed *GAL4*” and then stabilized the chromosome carrying each converted *LexA* driver over a balancer chromosome. Suggestions from students and instructors for improving the course included: (1) enhancing the RFP expression in future studies to ease the screening and identification of converted *LexA* lines and (2) considering additional visible phenotypes to identify converted flies, since the access to fluorescence stereomicroscope during this course was a significant “bottleneck.” To address these, we developed the v2 donor and tested its use in a second course (Supplementary Fig. 3b). This subsequent work (1) established balanced, “genetically stable” *LexA* lines in a uniform genetic background (*y*^1^*w*^1118^), (2) verified the *LexA.G4H*-dependent tissue expression of a GFP reporter, and (3) distributed new lines to a *Drosophila* stock center. In summary, these interscholastic curricula and collaborations established new CRISPR/Cas9-based strategies to generate *LexA* fruit fly lines and provided “proof of concept” for the feasibility of applying a genome editing curriculum in a secondary school setting.

## Discussion

To expand the collection of *LexA* drivers, we and others have generated novel *LexA* lines using enhancer trap screens (Kockel *et al.* 2016, 2019; Kim *et al.* 2023) or by cloning enhancers to direct the *LexA* expression (Pfeiffer *et al.* 2010; Wendler *et al.* 2022). While these approaches are sound, the novel lines generated by random transposon insertion or putative genomic enhancer fragments require extensive characterization, including insertion site mapping or expression specificity. As an alternative, complementary approach, CRISPR/Cas9 “HACK” strategies to generate *LexA* lines that recapitulate the tissue expression patterns of existing *GAL4* lines were recently developed. We have modified these approaches (Lin and Potter 2016; Chang *et al.* 2022) to generate new *LexA* lines, substantially simplifying the screening of HACK events using visible body color phenotypes (HACKy). The *GAL4*>*LexA.G4H* gene conversion can be subsequently confirmed by detecting the eye/ocelli expression of a second RFP marker. In multiple cases, we observed identical tissue expression patterns of reporter genes induced by the original *GAL4* and the cognate-converted *LexA.G4H* line, demonstrating the high fidelity of HACKy-mediated conversion. To address the demands for experiential science instruction, we worked with secondary school partners to develop curricula that systematically generated new *LexA* lines with well-characterized gene expression patterns. The *GAL4* lines were prioritized based on the characterization of the desired expression and frequency of cited usage (http://flybase.org/GAL4/freq_used_drivers/). Our work with student scientists demonstrates how university-based research could be leveraged to achieve educational outreach that also generates useful tools for the community of science.

Using the second chromosome–based v2 donor, the gene conversion efficiencies of second chromosome–linked *GAL4* lines were higher on average than those observed with third chromosome–linked *GAL4* lines. This indicates that *cis*-chromosomal HACKy remains more efficient than *trans*-chromosomal HACKy. Thus, additional lines to achieve *cis*-chromosomal HACKy of third chromosome–linked *GAL4* lines could be useful.

Prior studies showed that most nonconverted F2 males contain small deletions at target *GAL4* sequences, indicating the prevalence of nonhomologous end-joining (NHEJ) repair during HACK (Lin and Potter 2016). Thus, we speculate that after CRISPR/Cas9 DNA targeting, biasing HDR over NHEJ at double-strand breaks could improve conversion efficiency. One possibility to achieve this would be to construct donor strains with impaired NHEJ (Beumer *et al.* 2013).

Recent exciting advances in biology, like CRISPR gene editing, provide opportunities for secondary school instructors to refresh and invigorate curricula targeting nascent student scientists. To leverage this progress, we developed an experimental curriculum that: (1) incorporated several vibrant areas of bioscience, including genetics, molecular biology, bioinformatics, developmental biology, and evolutionary biology, (2) centered around a powerful modern gene editing technology (CRISPR/Cas9 and HDR) widely known to the general population that captured the interest of students and their instructors, (3) was based in fruit flies, a cost-effective, safe experimental system with rapid generation times suited for secondary school laboratory classes, that can (4) foster links between school-based data and discoveries with a global community of professional researchers. These courses benefitted from accompanying web-based instruction (see below) and could be readily adapted to suit shorter or longer instructional timeframes. For example, after generating, then improving donor fly characteristics (Fig. 1), and streamlining curricula (Supplementary Fig. 3), we updated our course at 2 Stan-X partner schools. These modifications are perhaps better matched to shorter instructional timeframes like summer terms or the inclusion of fruit fly experiments as a part of an existing advanced biology class. Although we focused on the frequently used *GAL4* lines in this study, university-based research groups have begun to “nominate” their own *GAL*4 lines for students in Stan-X programs to convert, thus fostering direct communication and a feeling of “ownership” and purpose in student collaborators. The corresponding author will be pleased to receive nominations and relevant instructional reading in the future from interested research groups.

To train instructors with little to no experience with *Drosophila* or CRISPR, we developed a week-long, intensive teacher training academy, called *Discover Now*. This approach of “teaching the teachers” has fostered the autonomy of Stan-X instructors and their schools (Chang *et al.* 2022; Wendler *et al.* 2022; Kim *et al.* 2023). Currently, partnering teachers from 4 additional schools are training to adopt HACKy-based experiments and instruction (S.P. and N.L., unpublished results). To provide practical guides for prospective research scientists and instructors interested in adopting this curriculum in their laboratory classes, the course manual (Supplementary Text 1) is also posted on the Stan-X website (https://www.stan-x.org/publications) and will be periodically updated. In summary, we developed experiment-based courses to provide genuine science experiences to secondary school students while generating useful tools for the community of science. This experiential instruction has introduced the wonder, anxiety, and joy of scientific discovery to secondary school students and informed their choices to pursue additional science training.

## Supplementary Material

jkad277_Supplementary_Data

## Data Availability

Strains and plasmids are available upon request. The NCBI GenBank accession number for pHACKy-GAL4 > nlsLexA::GADfl is OR687150. The course-teaching materials and syllabuses are also posted on the Stan-X website (https://www.stan-x.org/publications) and periodically updated. The authors affirm that all data necessary for confirming the conclusions of the article are present within the article, figures, and table. Supplemental material available at G3 online.
