# Supplementary material for: Simplified homology-assisted CRISPR for gene editing in Drosophila
Source: G3 (Bethesda). 2023 Dec 7;14(2):jkad277. doi: 10.1093/g3journal/jkad277 (PMC10849607; doi:10.1093/g3journal/jkad277)
Supplement: jkad277_Supplementary_Data [file jkad277_supplementary_data.zip › Figure_S3_G3-2023-404519.pdf]

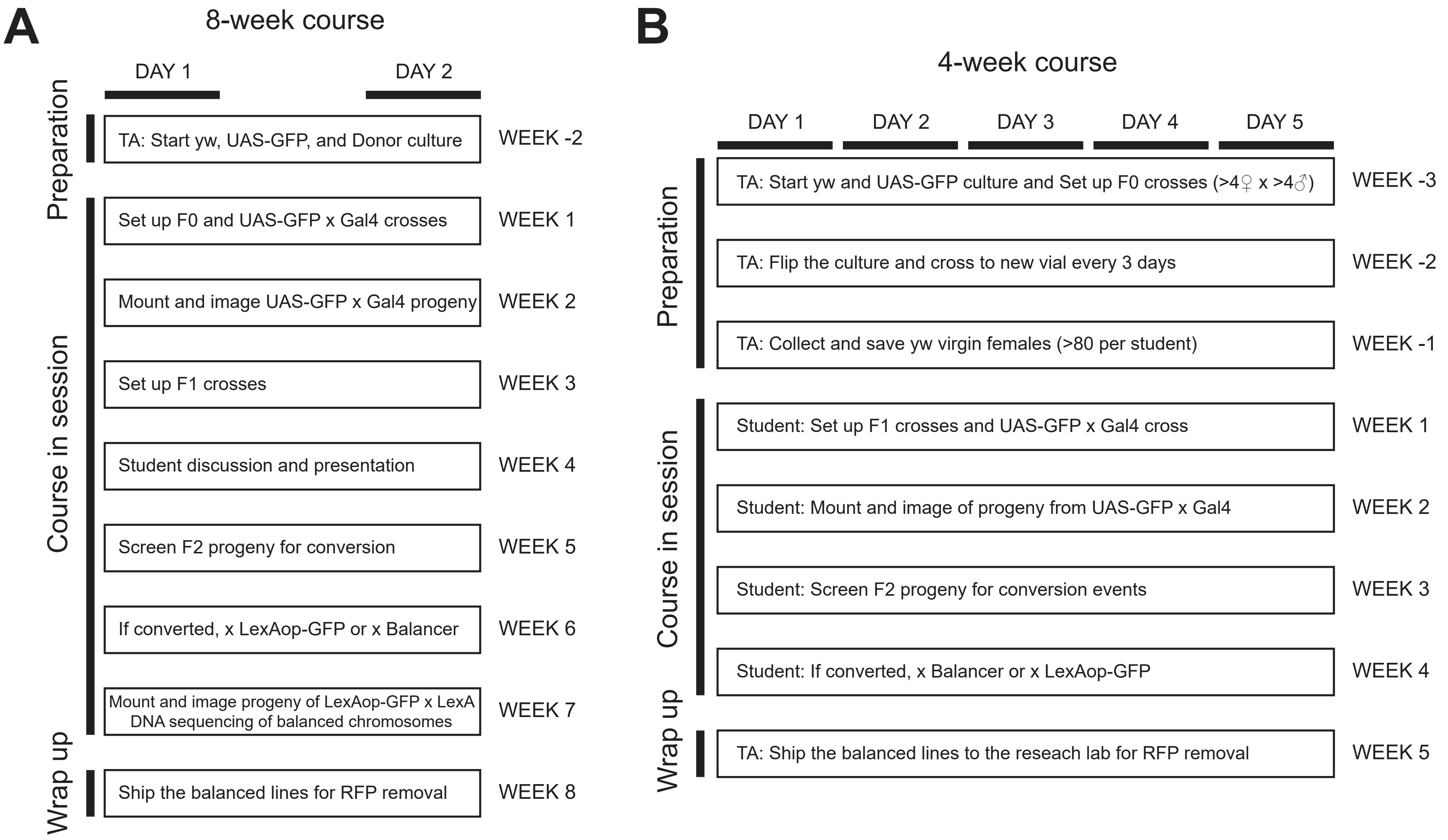
A
8-week course
DAY 1
DAY 2
Preparation
TA: Start yw, UAS-GFP, and Donor culture
WEEK -2
Course in session
Set up F0 and UAS-GFP x Gal4 crosses
WEEK 1
Mount and image UAS-GFP x Gal4 progeny
WEEK 2
Set up F1 crosses
WEEK 3
Student discussion and presentation
WEEK 4
Screen F2 progeny for conversion
WEEK 5
If converted, x LexAop-GFP or x Balancer
WEEK 6
Mount and image progeny of LexAop-GFP x LexA
DNA sequencing of balanced chromosomes
WEEK 7
Wrap up
Ship the balanced lines for RFP removal
WEEK 8
B
4-week course
DAY 1
DAY 2
DAY 3
DAY 4
DAY 5
Preparation
TA: Start yw and UAS-GFP culture and Set up F0 crosses (>4♀ x >4♂)
WEEK -3
TA: Flip the culture and cross to new vial every 3 days
WEEK -2
TA: Collect and save yw virgin females (>80 per student)
WEEK -1
Course in session
Student: Set up F1 crosses and UAS-GFP x Gal4 cross
WEEK 1
Student: Mount and image of progeny from UAS-GFP x Gal4
WEEK 2
Student: Screen F2 progeny for conversion events
WEEK 3
Student: If converted, x Balancer or x LexAop-GFP
WEEK 4
Wrap up
TA: Ship the balanced lines to the reseach lab for RFP removal
WEEK 5
