# Supplementary material for: Simplified homology-assisted CRISPR for gene editing in Drosophila
Source: G3 (Bethesda). 2023 Dec 7;14(2):jkad277. doi: 10.1093/g3journal/jkad277 (PMC10849607; doi:10.1093/g3journal/jkad277)
Supplement: jkad277_Supplementary_Data [file jkad277_supplementary_data.zip › Supplemental_Figure_and_Movie_Legends_G3-2023-404519.docx]

**SUPPLEMENTARL FIGURE AND MOVIE LEGENDS**

**Figure S1. Frequencies of conversion events from independent male germlines**

Phenotypic counting of F2 male progeny was plotted for each mating pair (Independent F1 Cross ID on X-axis). Black bars represent counts of F2 males with mini-white and non-curly phenotypes while red bars indicate F2 males with mini-white, non-curly, and RFP^+^, with frequencies written in red. For the second chromosome-linked GAL4 lines, each mating pair of a single F1 male and two *y*^1^ *w*^1118^ virgin females produced about 30 F2 males with desired phenotypes in a vial; the progeny size can be increased to 60 if F1 mating pairs are flipped once to a new vial after six days of initial mating. For X or third chromosome-linked GAL4 lines, these numbers decrease by a factor of one-half due to the independent segregation of donor and target chromosomes.

**Figure S2. Mating scheme for removing loxP-flanked transgene cassette and establishing stable LexA.G4H lines.**

A single F2 male carrying the converted LexA.G4H transgene was mated to Cre-expressing virgin females (BDSC 766). A single F3 male with two transgenes was mated with virgin females of *y*^1^ *w*^1118^ (BDSC 6598). All F4 males carrying the mini-white transgene were without RFP and yellow transgene expression, but a single F4 male with the mini-white transgene was selected to mate with virgin females carrying balancer chromosomes (e.g. BDSC 59967) to isolate the chromosome with LexA.G4H transgene without RFP and yellow transgenes. In the F5 generation, the chromosome carrying LexA.G4H was balanced to establish a “stable stock” in the *y*^1^ *w*^1118^ genetic background.

**Figure S3. Genetics laboratory class schedules deployed for 8 weeks and 4 weeks in secondary schools**

(A) A 90-minute-long class was held twice a week for 8 weeks. Sx students focused on experimental design, execution, and interpretation, and successfully converted assigned GAL4 lines (* in **Table 1**) over a 10.5-week schedule (Phillips Exeter Academy, NH). In week 1, students were introduced to *Drosophila* genetics including understanding genotypes, identifying associated markers, and setting up mating with follow-up maintenance. In week 2, students learned the anatomy of the third instar larva, micro-dissection, and imaging of slide-mounted tissues. In weeks 3 and 4, students generated a series of F1 intercrosses, while participating in discussions of prior characterizations of assigned GAL4 lines. In weeks 5 and 6, students started screening for conversion events in F2 progeny. In week 7, if a converted male was found, they set up a mating with LexAop-GFP reporter and documented GFP expression in the resulting progeny with RFP. The genomic DNA of the resulting progeny with RFP was sequenced to confirm the molecular conversion. In week 8, instructors and teaching assistants shipped the “converted (RFP^+^)” lines to research laboratories.

(B) A 6-hour-long daily class was held five days a week for 4 weeks to test the v2 donor and its use in a second course (The Lawrenceville School, NJ). To start week 1 with F1 mating, instructors and teaching assistants initiated F0 mating 3 weeks before the class started while collecting and maintaining virgin females of *y*^1^ *w*^1118^. Since the daily class schedule permitted students to master micro-dissection and imaging techniques more thoroughly, all students successfully documented GFP expression by assigned GAL4 lines by the end of week 2. Students had hour-long daily remote meetings with a research scientist to troubleshoot and discuss primary research articles.

**Movie S1. Live imaging of early pupa GFP expression in circulating hemocytes driven by either Hml-GAL4 (left) or Hml-LexA.G4H (right).**

**Text S1. Bio 670 Spring 2023 Manual v20230526**
