# Supplementary material for: Simplified homology-assisted CRISPR for gene editing in Drosophila
Source: G3 (Bethesda). 2023 Dec 7;14(2):jkad277. doi: 10.1093/g3journal/jkad277 (PMC10849607; doi:10.1093/g3journal/jkad277)
Supplement: jkad277_Supplementary_Data [file jkad277_supplementary_data.zip › Text_S1_G3-2023-404519.pdf]

A Manual for…

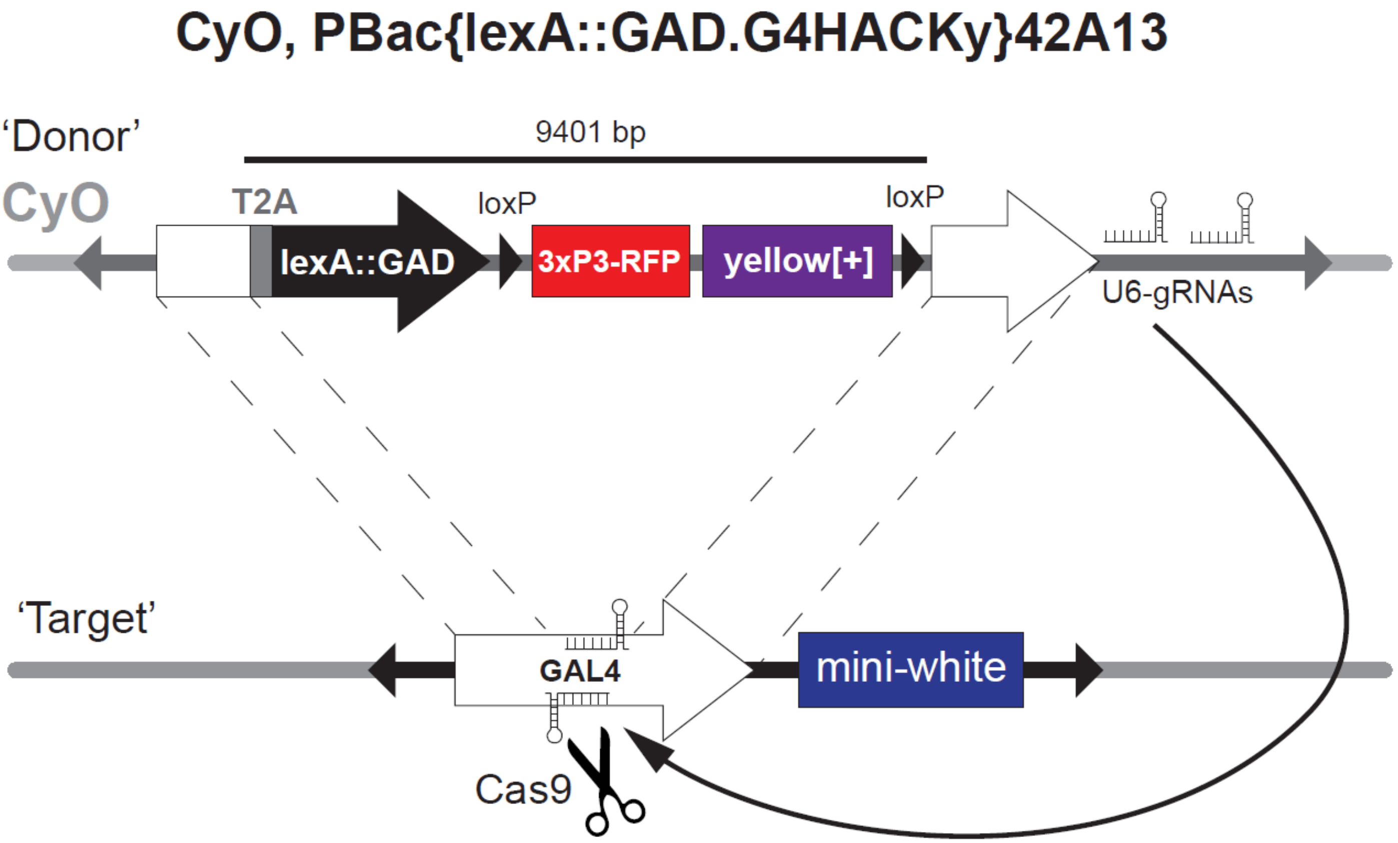

Many people contributed ideas and material to this manual - including…

Townley Chisholm, Anne Rankin, 2023 Bio670 students - Phillips Exeter Academy
Elizabeth Fox and Nicole Lantz - The Lawrenceville School
Seung Kim, Lutz Kockel and Sangbin Park - Stanford University School of Medicine

Table of Contents

Scope of this manual **4**
Dear Bio 670 Students, **5**
Why Drosophila? **6**
- Time to pick the fly's brain - Nature November 2007 6
- Small Flies - Big Discoveries: Nearly a Century of Drosophila Genetics and Development 7

What I need to know to read this manual & do the fly work **10**
- Genetic naming and writing conventions 11
- Genetic markers used in the crossing scheme 13
- Recognizing female and male flies, and recognize a virgin female 15
- Balancer chromosomes 17
- The meaning of a stable fly stock 20
- How to write transgene genotypes 21
- Some Hard Earned Suggestions for Fly Work 23
- Some Questions… 25

What is a binary expression system, which two are we discussing in this course and why is it helpful to have more than one? **28**
CRISPR **29**
- A Message from the Authors 29
- Section 1: The Origins 30
- Section 2: The Discovery 32
- Section 3: The CRISPR-Cas9 Mechanism 33
  - CRISPR in Bacteria 33
  - PAM Sequences 34
  - Acquisition 35
  - crRNA Biogenesis & tracrRNA 36
  - Interference 37
  - CRISPR in Gene Editing 38
- Section 4: Repair Methods after DSBs 40
  - Nonhomologous End Joining 40
  - Homology-Directed Repair 42
  - NHEJ vs. HDR 44
- CRISPR Glossary 45
- Use of CRISPR/HDR to Convert Gal4 Lines to LexA Lines and a Bit About the Gene Expression After the Conversion 47

Which Gal4 lines are we going to HACK and how to research a line? **50**
Crossing Program **59**
- Directions for Sorting the Offspring from the F1 Cross 60
- Problems to think about after collecting data from the F1 cross… 61

Problems to Understand the Crossing Program **62**
- Morgan Problem 62
- F0 Cross Problems 63
- F1 Cross Problems 65

F2 Cross Problems 69
The Drosophila Y Chromosome 70
Articles 71
Duffy Article, glossary and questions 71
Lin and Potter: Editing Transgenic DNA Components by Inducible Gene Replacement in Drosophila 73
Transgenic Drosophila lines for LexA-dependent gene and growth regulation 73
Red Queen Article 73
Imaging 74
The Imaging Crosses 74
Larval Dissection 76
Using PCR and Sequencing to Confirm HACK - Concept 79
Using PCR and Sequencing to Confirm HACK - Protocol 82
Fly Collection 82
Protocol for DNA Extraction 83
PCR 84
Gels 86
PCR Clean Up 87
Quantifying DNA Concentration 88
Stock Solutions 89
Sequencing Reactions 90
What happens to the stocks after they are stabilized but before they can be used by other researchers? 91
Remove Marker Transgenes from Stock 91
Enter stocks in Bloomington Stock Center 95

Scope of this manual

Genetic manipulation to perform functional experiments is the bread and butter of the fly biologist. There is a certain beauty in observing biological processes at work *in vivo.* To do so, the biologist needs tools as much as a carpenter does to get her/his work done.

The exercise delineated here is aimed at generating a widely applicable tool set and at making these tools available for the wider scientific community. This approach might be a departure from the classical and proven high school coursework, as everything might not work out as smoothly as envisioned beforehand. You will generate something new, and this novelty comes along with excitement but also frustration.

This is a manual guiding the conversion of Drosophila lines with GAL4 P elements inserted in different locations on chromosome 2 to LexA insertions; we will convert GAL4 P elements to LexA insertions by means of a crossing program that uses the CRISPR/Cas9 system. What this is and how to go about it is the content of this manual. We try to provide most, if not all the information you need, and nothing you don’t. Doing so makes the scope of this text somewhat limited, and further reading of other resources an absolute requirement. Please share the resources you use with us - your suggestions will make the manual better.

Dear Bio 670 Students,

We are very excited to work with you this spring in the “fly class” and think it might be helpful if we are very clear about our expectations at the outset. The expectations in this class are unique and we would like to know immediately if you think that you will be unable to meet them for any reason.

- Expect to be productive during class...if you finish your fly work before others we expect you to help another student or look for a task which needs doing. Please do not put us in the position of needing to ask you to refocus on the task at hand.
- Expect to attend every class meeting. There are obviously appropriate exceptions to this expectation and we hope you will talk to us if you need to miss class.
- Expect to be asked to work closely with a partner and be held accountable for the work of this partnership.
- Expect to come to the classroom to do fly work for evening and weekend homework at some points during the term.
- Expect to come in to work on Saturday and Sunday at some points in the term.
- Expect to spend time cleaning fly bottles, flipping flies to new food and doing other similar work. This maintenance work is required to make the course possible.
- Expect that the homework assignments may have to change based on what happens with our flies
- Expect that this course may not fit neatly into the homework guidelines. We will try very hard not to violate the rule broadly, but may ask more of you at certain points of the term than others.
- Expect to keep a lab notebook.

We recognize that the grading in this class may feel different than in most other classes. We will use canvas to record your grades. You will have nightly homework, quizzes, a test or perhaps two, take-home assignments, fly work checks and lab book checks.

You are not competing with each other. In an ideal world the flies cooperate fully, we successfully generate eleven new lines of flies, you all thrive in this class and you all earn A’s.

Please ask us if you have any questions about this information.

Best,
Mr. Chisholm and Ms. Rankin

Why Drosophila?

<u>Time to pick the fly's brain</u> - Nature November 2007

A wonderful perspective in 2007! I think this is still true in 2023. Discovering new neuronal circuits that govern unique animal behaviors are still done using fly genetics. In Seung Kim lab, we are currently betting on conserved genetics of aging and hormone-driven metabolism in human and fly. What students will do in the class is to generate additional fly lines that can control genetics of selected cells/tissues/organs (Sangbin Park)

Time to pick the fly's brain

Drosophila transformed developmental genetics and cell biology. Now the fruitfly is poised to help biologists decipher how the brain works.

Claude Desplan

One afternoon in 1997, a colleague called me into his office to announce that *Drosophila* research was all washed up. The fruitfly had amassed fantastic successes as a model system for developmental biology. In less than a decade, biologists had used it to map the genetic and molecular network that governs organism development. Now my colleague thought that, having served its purpose, *Drosophila* must be about to reclaim its status as the odd little creature obscure scientists used to manipulate genetic characters. How wrong he was.

In the early 1980s, basic research exploded. Working on *Drosophila* was valued as a way to understand phenomena believed to be unique to flies, such as weird body deformations. If the *Drosophila* pioneers believed that their work might have broader relevance, they could not have dreamed of its awesome implications.

A huge breakthrough came in 1984 with the discovery of the homeobox, the Rosetta Stone of developmental biology. This piece of DNA is shared by the genes that govern body pattern. Mutations in these 'homeotic' genes cause body parts to transform into one another — a leg grows where an antenna should be, four wings develop instead of two, and so on. This homeobox sequence was soon found to have similar functions in mice, humans and indeed most animals.

The discovery transformed vertebrate developmental biology. Biologists identified the molecules underlying what had been abstract concepts, such as morphogenetic gradients — rising or falling amounts of a single protein that direct the development of different body parts. To the surprise of most, it became clear that *Drosophila* use the same developmental genes as vertebrates. For more than ten years, there was formidable excitement as each new fly gene clarified how a mouse embryo develops, an organ forms or what causes a human mutation.

Things went so fast that by the late 1990s it seemed that the fruitfly had reached its full potential for answering big questions, and that developmental biology should be left to those working on vertebrates. By then, fly scientists had made great advances that relied more on clever tricks and a century of genetics than on expensive equipment. A few drosophilists defected to plants or zebrafish in an attempt to adapt these techniques, but the fly stalwarts kept going.

By the start of the new millennium, immensely powerful methods, such as the ability to generate a single mutant cell in an otherwise normal organism, helped solve the major challenge of the time: understanding how cells communicate.

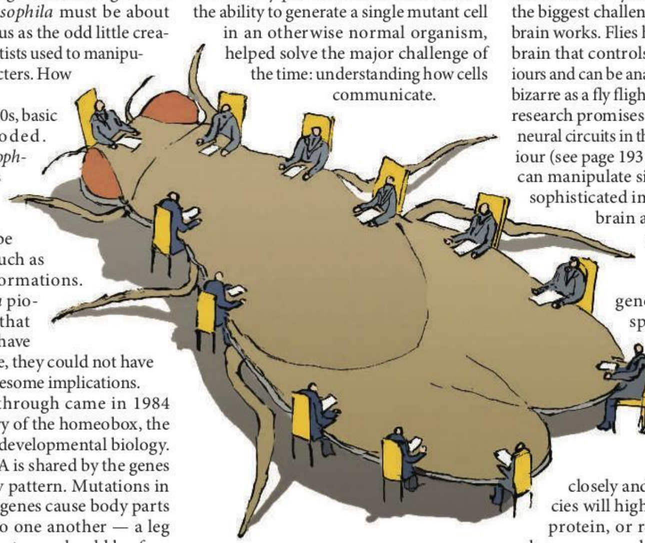

J. KAPUSTA

Most molecular cascades that bring a signal from the cell surface to the nucleus where it can change the fate of a cell were discovered in flies. Their function was then further analysed in cultured mammalian cells.

In fact, the entire field of cell biology benefited tremendously from work in *Drosophila*. Logic says processes that concern single cells should be probed in yeast or cultured mammalian cells. But the fly oocyte and embryo proved to be ideal test beds for studying events of the single cell. A purely mechanistic description of molecular interactions was superseded by an understanding of how coordinated events take place in the context of a whole organism.

Immunity is another area in which *Drosophila* had a surprisingly big role. Findings in flies uncovered the molecular pathways that recognize and then fight bacterial, fungal or viral infections without relying on previous encounters with the pathogen. The same molecules and pathways were later shown to have similar roles in humans, which led to the revival of innate immunity, a field with far-reaching applications in medicine.

What can *Drosophila* still deliver that will change biology again? The time is ripe to use these tiny creatures to understand the biggest challenge in biology: how the brain works. Flies have a relatively simple brain that controls sophisticated behaviours and can be analysed with machines as bizarre as a fly flight simulator. *Drosophila* research promises to solve how complex neural circuits in the brain mediate behaviour (see page 193), now that researchers can manipulate single neurons and use sophisticated imaging of the working brain and electrophysiological techniques.

This issue of *Nature* reports the whole-genome sequences of 12 species of *Drosophila*, which will allow the organism that can be manipulated so exquisitely to have the best annotated genome. Comparing the genomes of closely and distantly related species will highlight which parts of a protein, or regions of DNA, have been conserved during evolution, and so are likely to have a function. This will offer a way to decipher the grammar of regulatory DNA, which has so far proved elusive.

Powerful genetic tools, genomic advances and beautiful imaging of the tiny structure of a fly brain or embryonic tissue have kept fruitfly research booming for more than two decades. By enabling us to pose biological questions in an *in vivo* context, the fly is an ideal subject for integrating molecular or cellular processes in the biology of the whole organism. *Drosophila* research is thriving and should live up to our hopes for many decades to come. ■

Claude Desplan is in the Department of Biology at New York University, 1009 Silver Center, 100 Washington Square East, New York, New York 10003, USA.

For further reading see www.nature.com/nature.

ESSAY

©2007 Nature Publishing Group

DEVELOPMENTAL DYNAMICS 232:526–528, 2005

PERSPECTIVE

Small Flies—Big Discoveries: Nearly a Century of *Drosophila* Genetics and Development

Anthea Letsou*† and Dirk Bohmann†

It was almost 100 years ago, in 1909, that a classically trained embryologist, Thomas Hunt Morgan, chose the fruit fly *Drosophila melanogaster* as a model organism for an experimental study of evolution. Ever since Morgan's auspicious choice of the fruit fly as an experimental organism, scientists have been eyewitnesses to the "awesome power" of *Drosophila* genetics—from the transmission geneticists best exemplified by Thomas Hunt Morgan and his students to the developmental geneticists who have been led by Ed Lewis, Christiane Nüsslein-Volhard, and Eric Wieschaus. There is no doubt that, even now, in the post-genomic age of the 21st century, we find ourselves ever indebted to our genetics forebearers.

Building on the productivity of previous generations, present day *Drosophila* scientists continue to establish paradigms and achieve technological breakthroughs that help advance not only fly research but many different fields of the life sciences as well. Here, we give a short overview of the history of *Drosophila* genetics. We hope that an understanding of how we got where we are today and an appreciation of past discoveries will help to place the current excitement about genomes, molecules, and mechanisms within the context of a long-established scientific culture and history of *Drosophila* experimentation.

TRANSMISSION GENETICS IN *DROSOPHILA*

In the earliest years of the twentieth century, a founding generation of geneticists focused on problems of transmission. They exploited the fruit fly to answer classic questions such as, how are genes inherited? What is a chromosome and how does it recombine? Arguably the greatest breakthroughs in this arena were those of the first *Drosophila* geneticists: Thomas Hunt Morgan and his students, Calvin Bridges, Alfred Sturtevant, and Hermann Muller. It is perhaps somewhat surprising to realize that Morgan chose his organism as an anti-Mendelist, hoping to disprove the canons of genetics that we hold so dear today. After two "wasted" years of attempting to induce mutations in *Drosophila* by altering selective pressures, Morgan spotted a mutant white-eyed *Drosophila* male in his culture of wild-type red-eyed flies. The rest is history, as Morgan's analysis of *white* inheritance quickly led him to abandon his evolutionary studies and to embrace the "rediscovered" genetic theories of Mendel.

Morgan demonstrated that genes are on chromosomes. The studies for which he earned the Nobel Prize in Medicine (1933)—"discoveries concerning the role played by the chromosome in heredity"—are elegant in their simplicity. In *Drosophila* breeding experiments, Morgan showed us that transmission of *white* eye color is linked to inheritance of the X chromosome, and thus eye color and gender are linked traits. This seminal finding led Morgan to conclude that some genetic traits are not inherited independently (as Mendel had supposed) but rather must be linked (Morgan, 1910).

Morgan's students, Bridges, Muller, and Sturtevant, went on to no less illustrious careers of their own. Bridges used the fruit fly to prove that chromosomes are structures of inheritance; thus, we began to understand the cellular basis of heredity. As an aside, it is notable that Bridge's landmark study of nondisjunction was quite possibly a victim of capricious review. Indeed, Morgan was sufficiently irritated by the rejection of his student's manuscript from the *Journal of Heredity*—a premier journal of the day—that he and his colleagues founded a new journal, *Genetics*, that still exists. Bridge's manuscript launched the inaugural issue (Bridges, 1916).

Bridge's contemporary, Hermann Muller, used the fruit fly to identify and physically map chromosomal aberrations and perhaps even more importantly to decipher the chemical nature of mutation (Muller, 1927). As was his mentor before him, Muller too was the recipient of a Nobel Prize (1946)—"for the discovery of the production of mutations by means of X-

†The authors are Guest Editors of this Special Issue on Drosophila as a Model System.
Department of Biomedical Genetics, University of Rochester Medical Center, Rochester, New York
***Correspondence to: Anthea Letsou, Eccles Institute of Human Genetics, 15 North 2030 East, Room 2100, University of Utah, Salt Lake City, UT 84112. E-mail: aletsou@genetics.utah.edu**

DOI 10.1002/dvdy.20307
Published online 9 February 2005 in Wiley InterScience (www.interscience.wiley.com).

© 2005 Wiley-Liss, Inc.

ray irradiation". There can be no doubt as to Muller's intelligence and astuteness; well before the onset of the atomic age in which we now live, Muller anticipated and articulated the genetic risks we would face as a consequence of the irresponsible and weaponized use of ionizing radiation (Muller, 1946). Importantly, the implications of his irradiation studies were not overlooked when we did finally enter the atomic age. In response to Muller's experimental discoveries and musings, extensive genetic studies in *Drosophila* (and the mouse) were undertaken to assess the relative mutation rates in these model organisms as an indicator of the genetic risk posed to humans from the utilization of atomic energy.

The third of the Morgan student triumvirate was Alfred Sturtevant who used the fruit fly to demonstrate that chromosomes constitute a linear array of genes (Sturtevant, 1913). In addition to bolstering the chromosome theory of inheritance, Sturtevant's work is notable for its application of mathematics to biology. In fact, his study highlighted an emerging trend in biological method—this was a replacement (or at least supplementation) of descriptive science with an approach that was both more analytical and mathematical. Taken altogether, Morgan and his students' use of breeding experimentation in the fruit fly *Drosophila* led them to an enviable record of achievement. Their contributions, which are certainly far too extensive to enumerate here, have been fully discussed and especially nicely annotated with respect to the historical record by Sturtevant (1965).

Before leaving the contributions of the classical geneticists, two additional issues (one scientific, the other societal) warrant mention here. First the scientific: T.S. Painter, a student of Theodore Boveri who in 1903 along with Walter Sutton proposed that chromosomes contain genes, discovered the *Drosophila* giant salivary gland chromosomes. Importantly, Painter understood that this biological material offered an excellent opportunity to visualize chromosome structure (Painter, 1934). Second the societal: Morgan in addition to establishing the fruit fly as a model genetic organism endowed us with a new scientific culture: "The open, critical, yet fully democratic and egalitarian atmosphere that was evident in the Fly Room soon came to characterize the distinctively American atmosphere of university research—an especially significant development as American graduate education increasingly became the model for graduate education throughout the world" (Kandel, 1999). Given the rich intellectual atmosphere and the relatively free exchange of ideas in Morgan's fly room, it is perhaps not too surprising that, in addition to Muller, another of Morgan's trainees as well as two of his "academic grandchildren," also went on to win Nobel prizes: George Beadle (1958), Joshua Lederberg (1958), and Ed Lewis (1995).

FROM FLIES TO CELLS

In subsequent years (at the end of the 1930s and for most of the 40s), the rules of mitosis and meiosis as well as the nature of the gene were elucidated, but not in the fly. For this relatively short period in our modern scientific history, the modest fruit fly fell from experimental favor. It did, however, re-emerge as an experimental organism with the birth of molecular genetic analysis: Boris Ephrussi and George Beadle used the fruit fly to give biochemistry and molecular genetics an initial experimental push. Ephrussi and Beadle transplanted larval eye discs from genetically marked larvae into the abdomens of genetically dissimilar larvae. Here, a third eye could develop ectopically and experimenters distinguished between tissue autonomous and nonautonomous requirements for gene products (Beadle and Ephrussi, 1936). *Drosophila* mosaic studies, like these, set the stage for mosaic studies in a wide variety of experimental organisms. The ability to deliberately replace wild-type genes with gain- and loss-of-function alleles in almost any setting and time frame using the UAS-Gal4 (Brand and Perrimon, 1993) and FLP-FRT (Golic, 1994) binary gene regulatory systems (or any of their various imaginative permutations) has proven invaluable in deciphering the rules by which cells interact with one another to control cell growth and differentiation.

MUTATION ANALYSIS AND DEVELOPMENTAL GENETICS IN *DROSOPHILA*

When most of us think about genetics, we think of mutants. Although surely not synonymous terms, one most certainly often invokes the other. But how we used mutants as tools of learning differed dramatically in the early and late parts of the twentieth century. Until the 1970s, investigators had collected mutants, by and large as chromosomal markers that facilitated the study of chromosome mechanics. Although the first fly "monster"—one with two sets of wings—was discovered in 1916, it was not until the 1970s that the idea that single genes could lead to morphological "transformations" was considered experimentally. This intellectual leap was recognized by the 1995 Nobel committee in their tribute to three "modern" *Drosophila* geneticists: Ed Lewis, Christiane Nüsslein-Volhard, and Eric Wieschaus, "for their discoveries concerning the genetic control of early embryonic development."

Among Ed Lewis' most significant contributions was his demonstration that single genes, members of the homeotic gene family, could lead to developmental transformations (Lewis, 1978). Homeotic genes, now mostly referred to by their molecular name Hox genes, have been recognized since as principal regulators of pattern in flies, mice, and humans. The notion that Hox genes are endowed with transforming capacity revolutionized our understanding of development. This issue of *Developmental Dynamics* is dedicated to the memory of Ed Lewis, who passed away last year, and his life and scientific contributions are described in fuller detail in two commentaries (Lipshitz, 2005; Sakonju, 2005).

The contributions of Christiane Nüsslein-Volhard and Eric Wieschaus to developmental biology nicely complemented those of Lewis. This team's saturation mutation screening efforts led to our understanding that genes can be grouped together bases upon their shared loss-of-function phenotypes. Nüsslein-Volhard and Wieschaus suggested that (1) shared loss-of-function phenotypes define genes functioning in single biochemical pathways, and (2) related (but not

identical) phenotypes define genetic hierarchies (Nüsslein-Volhard and Wieschaus, 1980).

At approximately the same time that Nüsslein-Volhard and Wieschaus initiated their screens, molecular biology methods were being harnessed in labs world-wide. Coupling this technological boon with a concurrent emerging understanding of how transposons function in fruit flies (Spradling and Rubin, 1982) allowed a second generation of developmental geneticists to identify the gene products associated with the mutants. Thus, tremendous advances in our understanding of embryonic development in flies were the prizes associated with the Heidelberg screens. Happily, the conservation of regulatory mechanisms defined in the fruit fly over the organismal spectrum of evolution has made it possible to use the *Drosophila* to facilitate our understanding of development and disease in higher eukaryote—most importantly in humans.

FLIES 'R US

Within the context of a century of progress, we have finally entered the genomic and postgenomic ages of *Drosophila*-facilitated discovery. With Gerry Rubin at the helm of a collaborative undertaking by the Berkeley *Drosophila* Genome Project and Celera Genomics, Inc., the fruit fly genome sequence was completed in 2000 (Adams et al., 2000). Comprising approximately 14,000 genes, the *Drosophila* genome has provided us with a new wealth of information as well as the final validation of *Drosophila* as a first-class model organism. Developmental biologists have long accepted as dogma that what we learn in the fruit fly can be extended to higher eukaryotes. But now there is the code itself—the fly blueprint, which is remarkable in its likeness to our own. Indeed, when compared with mammalian proteins and expressed sequence tags, more than half of the fly proteins have similar mammalian counterparts at a statistical cutoff of $E < 10^{-10}$, compared with 36% and 38% for worm and yeast, respectively (Rubin et al., 2000).

CONCLUSIONS

For approximately 100 years, experimentalists have taken advantage of *Drosophila*'s small size, the low cost and ease with which it can be cultured, its high fecundity and short life cycle, its small chromosome complement, and its ability to withstand mutation and crossbreeding experiments. These past years of successful experimentation and productivity bode well for the next century. Indeed, *Drosophila* is not about to be retired as a model system for cutting edge research to address pressing questions in biology. In addition to the features that have made *Drosophila* so amenable to study for the past century, new and powerful resources and experimental possibilities make the research as exciting and attractive as ever in the past century for young and established scientists. We await the genome sequence of 10 or more *Drosophila* species in the near future, and these will provide a tremendous playing field for bioinformatics approaches to development and organism function. Targeted genome manipulations are possible and will become routine, sophisticated methods of imaging will permit a direct view into the connections between cell topology and function in intact tissues. The powerful and public resources—FlyBase, stock centers, genome projects—built up by the fly community, and by Bill Gelbart and Thom Kaufman in particular, will continue to provide valuable tools for the challenges that we will face and enjoy in the next century. Of course, of immense importance to our continued success will be the excitement and ingenuity of a new generation of open-minded and relentless scientists.

REFERENCES

Adams MD, Celniker SE, Holt RA, Evans CA, Gocayne JD, Amanatides PG, Scherer SE, Li PW, Hoskins RA, Galle RF, et al., 2000. Comparative genomics of the eukaryotes. Science 24:2204–2215.

Beadle GW, Ephrussi B. 1936. The differentiation of eye pigments in *Drosophila* as studied by transplantation. Genetics 21:225–247.

Brand A, Perrimon N. 1993. Targeted gene expression as a means of altering cell fates and generating dominant phenotypes. Development 118:401–415.

Bridges CB. 1916. Non-disjunction as proof of the chromosome theory of heredity. Genetics 1:1–52.

Golic KG. 1994. Local transposition of P elements in Drosophila melanogaster and recombination between duplicated elements using a site-specific recombinase. Genetics 137:551–563.

Kandel ER. 1999. Thomas Hunt Morgan at Columbia University: Genes, chromosomes, and the origins of modern biology. Columbia Magazine (fall).

Lewis EB. 1978. A gene complex controlling segmentation in *Drosophila*. Nature 276:565–570.

Lipshitz HD. 2005. From fruit flies to fallout: Ed Lewis and his science. Dev Dyn 232:529–546.

Morgan TH. 1910. Sex limited inheritance in *Drosophila*. Science 32:120–122.

Muller HJ. 1927. Artificial transmutation of the gene. Science 46:84–87.

Muller HJ. 1946. Nobel lecture. Available at: http://nobelprize.org/medicine/laureates/1946/muller-lecture.html.

Nüsslein-Volhard C. Wieschaus E. 1980. Mutations affecting segment number and polarity in *Drosophila*. Nature 287: 795–801.

Painter TS. 1934. A new method for the study of chromosome aberrations and the plotting of chromosome maps in *Drosophila melanogaster*. Genetics 19:175–188.

Rubin GM, Yandell MD, Wortman JR, Gabor Miklos GL, Nelson CR, Hariharan IK, Fortini ME, Li PW, Apweiler R, Fleischmann W, et al. 2000. The genome sequence of *Drosophila melanogaster*. Science 287:2185–2195.

Sakonju S. Remembering the year with E.B. Lewis. 2005. Dev Dyn 232:547–549.

Spradling AC, Rubin GM. 1982. Transposition of cloned P elements into Drosophila germ line chromosomes. Science 218: 341–347.

Sturtevant AH. 1913. The linear arrangement of six sex-linked factors in *Drosophila*, as shown by their mode of association. J Exp Zool 14:43–59.

Sturtevant AH. 1965. A history of genetics. Cold Spring Harbor, NY: Cold Spring Harbor Laboratory Press.

What I need to know to read this manual & do the fly work

The *Drosophila* Genome….

…is diploid and organized on four groups of chromosomes. A chromosome is a continuous strand of DNA with two telomeres (“end pieces”) and a centromere (a specialized region utilized in cell division/mitosis). The chromosomal groups are: the sex chromosomes, called X and Y, and 3 autosomal groups called chromosomes 2,3, and 4. So every cell (with the exception of germ cells that have gone through meiosis in the germ line) has a pair of sex chromosomes (either XX or XY), and a pair of each of the autosomes. From a practical point of view here, the Y and chromosome 4 can be ignored. The Y behaves funky and carries very few genes, and chromosome 4 is this little stump. Autosomes can be abbreviated with their roman numeral. If you read *brown* is on II, it means that this gene called *brown* is on the second chromosome.

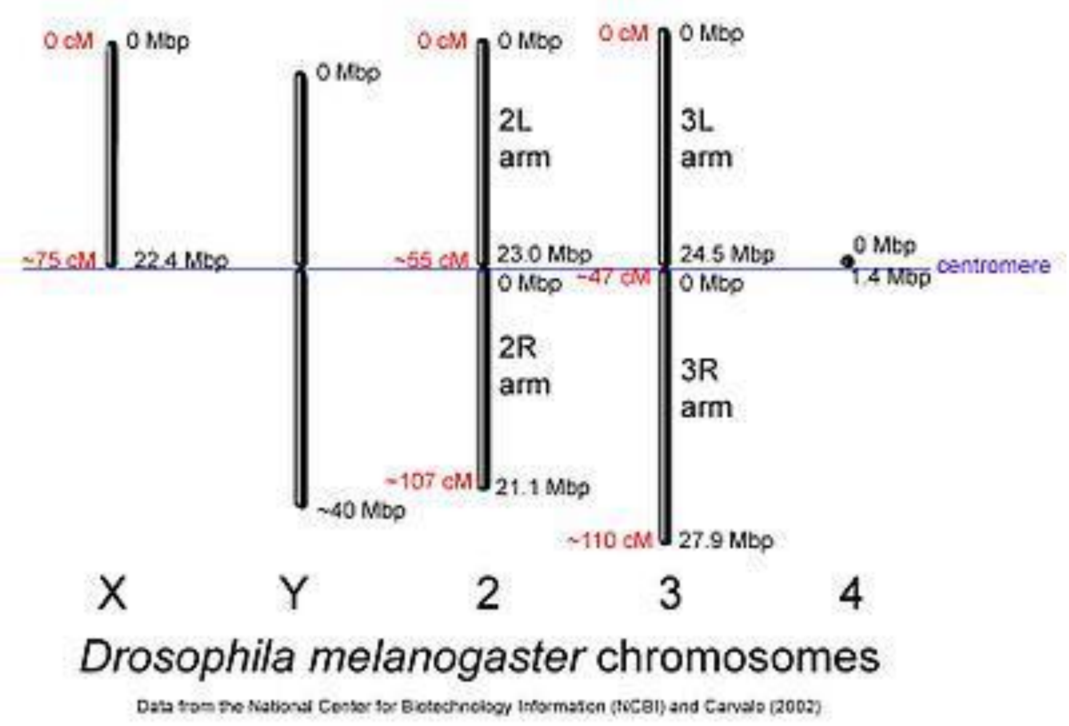

The *Drosophila* genome is 139 x 10^6 base pairs long, abbreviated 139 Mbp (Mega base pairs) and encodes approximately fifteen thousand genes. The number of genes is a ballpark number and prone to change in the future, although probably not as much as it has in the past.

The deciphering of the function of individual genes and functional genetic networks in tissues and developmental time is driving our understanding of biology.

Genetic naming and writing conventions

Drosophila genes used to be named after their loss-of-function phenotype. Probably the most famous example is the *white* gene. The White protein (the protein encoded by the *white* gene) encodes a protein required to make red eye pigment. However, a mutation rendering this gene non-functional results in failure to produce this red eye pigment. Hence, the eye is white. Hence the gene that makes red eye pigment is called white. This seems initially counter-intuitive and is a little tough to digest, but one can get used to it. Loss-of-function names like *cheapdate* and *I'm-not-dead-yet* indicate susceptibility to alcohol intoxication and longevity, respectively.

In more recent days, gene names emphasize more the functional group the gene belongs to. For example, the ortholog of human EGFR (Epidermal Growth Factor Receptor) is called DER (Drosophila Epidermal Growth Factor Receptor). An ortholog is a gene of similar function and activity in a different species. An orthologous gene pair frequently shares a decent degree of amino acid similarity.

Here is another convention: *genes* are written in small caps *italics*. Yes, even when they are at the beginning of a sentence. Proteins start with a capital letter. These are details right now, but sometimes it is important to distinguish between the *gene* and the encoded protein product. Then the difference comes in pretty handy.

Like all things, gene names have abbreviations or acronyms. In the case of the *white* gene it is *w*. The wild type (fully functional) *white gene* can be written down as w^{+}. As most geneticists are lazy slobs, genes in their wild type configuration are actually not written into the genotype at all. Again, this shortcut means that everything that is not in a particular genotype (meaning: mutated) is wild type. Conversely, a mutated form of *white* is put down as w^{-} or simply as *w*. In more specific cases, the specific allele (variant of a gene, frequently loss or gain-of-function) name is integrated in the superscript, e.g. w^{1118}, alternatively written as *w[1118]*. You now know that if there is no w^{-} in the genotype, the eyes should be red. Unless there are other eye color mutations in the genotype that make the eyes brown. Or cinnabar. Or vermillion. Or pink. And so on.

When the entire genotype of a specific fly strain across chromosomal groups is written out, the chromosomal groups are separated by semicolons, and alleles located on the same chromosome are separated by commas. To deal with the diploid nature, the two chromosomes of the same chromosomal group are separated by a division line. Please note that the two second chromosomes do not need to be identical, especially in the offspring of a cross of two genetically different parents. However, convention has it that if the two chromosomes of a homologous pair are identical, this division line thing falls flat and only the genotype of one chromosome is written out. And one more rule: if there are mutant alleles on one chromosome, but none on the other homolog, this wild type chromosome is put down as +.

<u>Example 1:</u> *white* mutant fly (side note: *w* is located on the X)

w-/w- or $\frac{w}{w}$ or simply *w* (if it is a female) or *w-/Y* (if it is a male)

Example 2: *white* heterozygote female:

w-/+ or $\frac{w}{+}$

So far, we dealt with recessive markers only. That means that both copies need to be mutated to show the phenotype. But there are also dominant alleles. They show their phenotype already when present on one chromosome only, while the allele on the other chromosome is still *wild type*. New rule: alleles that cause dominant phenotypes are written first letter in caps. We will need a couple of them in the course of this work. One is *Cy* (*Curly*, causes curly wings), the other one is *L*, (*Lobe*, causes an eye reduced in size). Both are located on the second chromosome.

Example 3: *white* mutant that carries *L* on one of its second chromosomes, and *Cy* on the other:

w-/w-; L/Cy or $\frac{w}{w}$; $\frac{L}{Cy}$ (if it is a female) *w-/Y; L/Cy* or $\frac{w}{Y}$; $\frac{L}{Cy}$ (if it is a male)

Genetic markers used in the crossing scheme

All the images here are culled from the marvelous poster "Learning to Fly".

First, the eye markers: *white* (*w*-), *Lobe* (*L*) compared to *wild type* (*wt*)

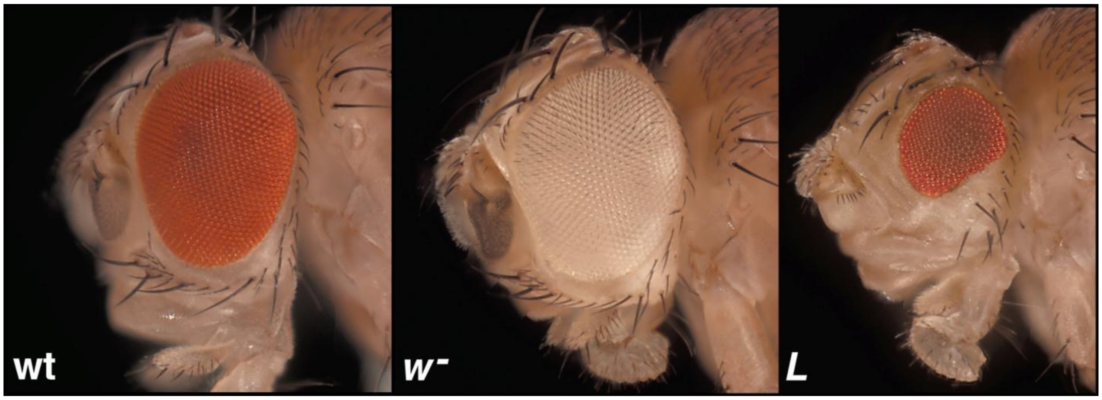

Next, the famous *Curly* (*Cy*), dominant on second chromosome compared to *wt*

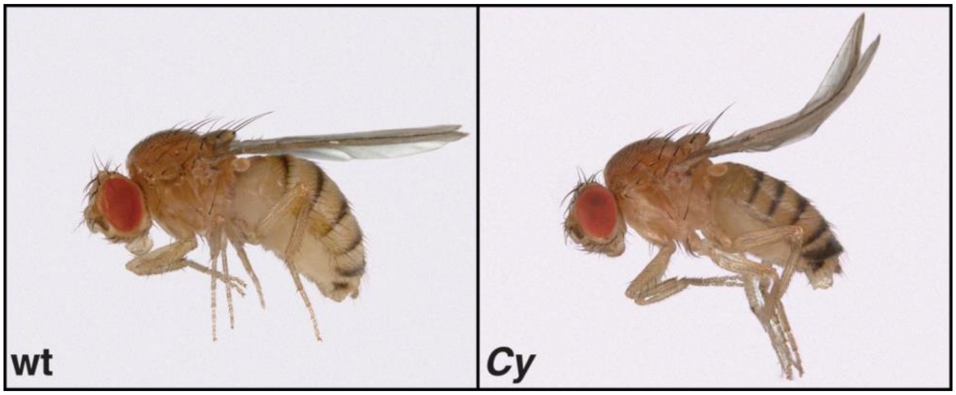

Third chromosomal dominant markers: *Humeral* (*Hu*). Extra humerals (a specific kind of bristle on thoracic segment number one). Some would say: Extra hairs on the shoulders. Or: "They wear epaulettes''. This is so wrong, but everybody understands immediately…. Sometimes it is difficult to see on one side of the fly, so turn the fly around and look at the other side when in doubt.

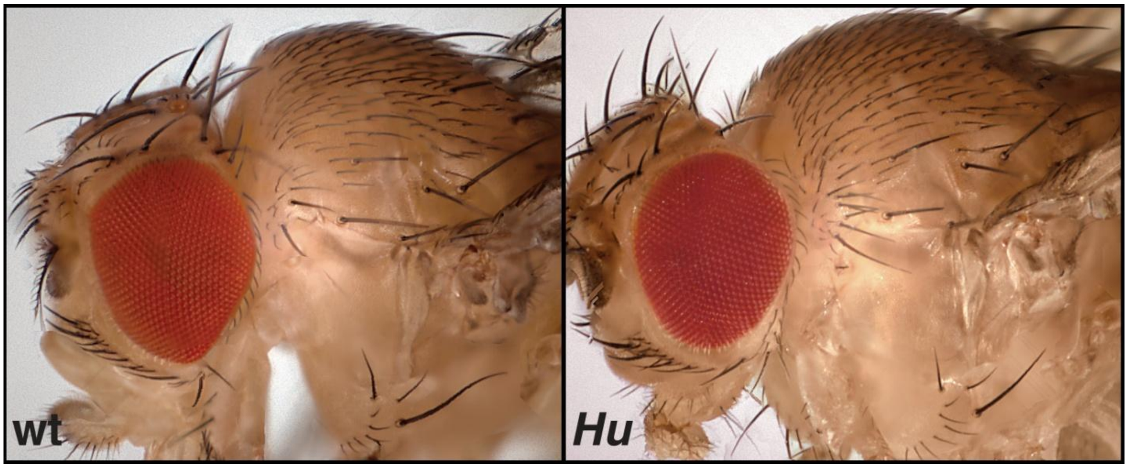

And finally, wild type body color compared to yellow body and ebony body.

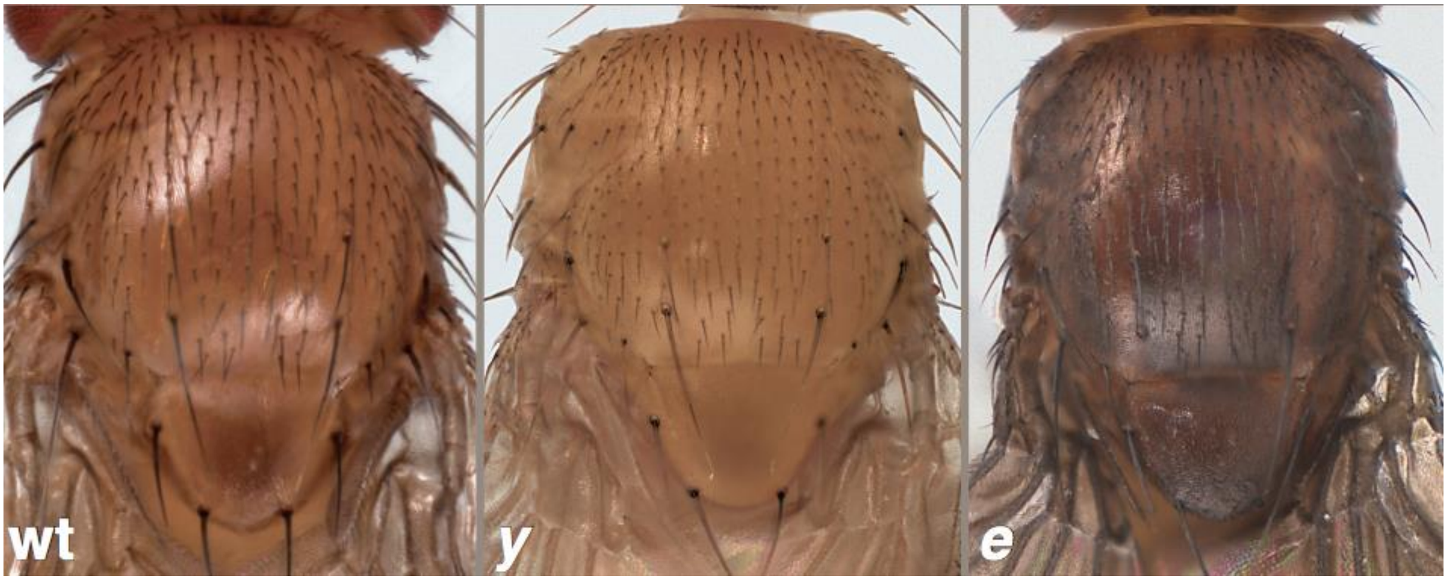

Here is a table to summarize some of this information…

Trait	Alleles	Location	Phenotype	Dominance Relationship
eye color	wild type (w+)	X	red	w+>w-
	White (w or w-)	X	white	
	Mini-white (w+)	in a p element	red	
	RFP+	in a p element	red under fluorescent light	
	GFP+	in a p element	Green under fluorescent light	

Eye Shape	wild (+)	II	wild	L>+<br>(L/L is lethal)
	Lobed (L)	II	Smaller eye	
Body Color	y+	X and can be in a p element	wild	$e>y^+>y^-$
	y-	X	yellow	
	e or e^-	TM6B balancer and III	ebony	
Shoulder Hairs	Humeral (Hu)	TM6B balancer and III	More than 2 hairs on shoulder	Hu>+<br>(Hu/Hu is lethal)
	wild	III	Two hairs on shoulder	
Wing Shape	Curly (Cy)	CyO balancer and II	2 Curly wings	Cy>+<br>(Cy/Cy is lethal)
	wild	II	2 straight	
Wing Number	Ultrabithorax (Ubx)	TM2 balancer and III	4 wings	Ubx>+<br>(Ubx/Ubx is lethal)
	Wild (+)	III	2 wings	
Bristles on back	Stubble (Sb)	III	Shorter, thick bristles on back	Sb>+<br>(Sb/Sb is lethal)
	Wild (+)	III	Longer, thinner	

Recognizing female and male flies, and recognize a virgin female

The flies shown here are a couple of days old. Males have a section on the distal end of the dorsal (“back”) side of their abdomen that is darker pigmented than the females. Males are also smaller than females, but this is difficult to detect in freshly hatched flies. Furthermore, in males the ventral (“belly”) side shows a couple of bristles the female does not have; the bristles mark their penis apparatus.

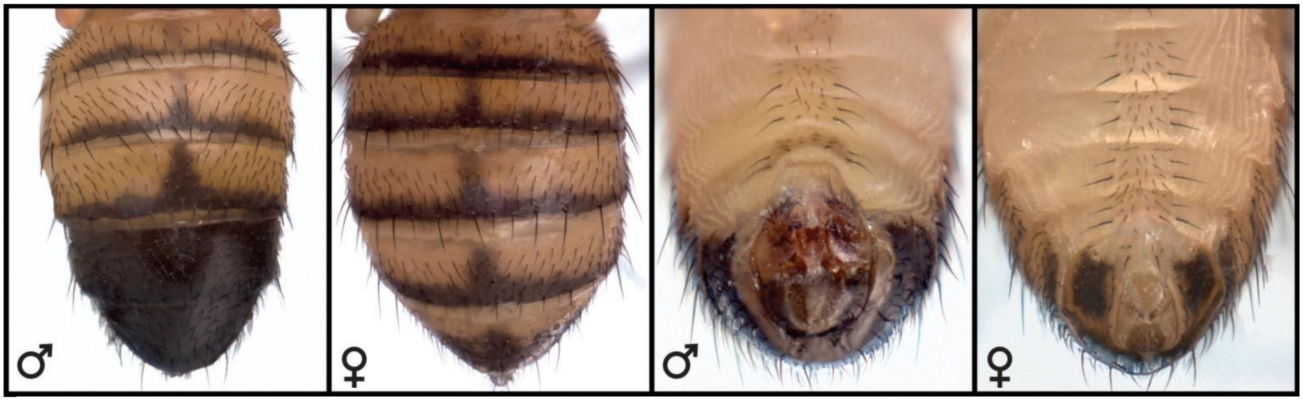

Male dorsal abdomen **Female dorsal abdomen** **Male ventral abdomen** **Female ventral abdomen**

Why is collecting virgin females important? All genetic crosses work towards the construction of a specific genotype. If the female has been mated already, it is often difficult to assess what the genotype of the offspring is. The easiest way to make sure that the geneticist knows offspring genotypes is using females that have not been mated. These are female virgins. The easiest way to make sure that females are virgins is collecting freshly hatched females.

Recognizing female virgins: as a rule of thumb, at 25° C all females stay virgins for 8 hrs after they hatch. So the easiest way is to dump out all flies in the morning, wait a couple of hours, and collect the females. All of them should be virgin. However, this dumping out thing is a little wasteful. If your cross produces only a couple of flies, and half of them get thrown out, you might have to give up the F_4 crossing scheme because of the lack of suitable flies in F_2. Luckily, there are anatomical tell-tale signs signaling that a fly is freshly hatched. They are: soft cuticle (the hind legs leave a “indentation” in the side of the fly), weak, light pigmentation (watch out, this makes males and females much more look alike), and a spot of meconium shimmering through the ventral side of the cuticle. In some instances, when flies have just crawled out of the pupal case, the wings are still folded up and look “wrinkled”. Later on, the wings can have an opaque shimmer. In most instances the abdomen looks kind of inflated because the cuticle has not hardened yet and the pressure of the internal organs is pushing outward and inflating the exoskeleton.

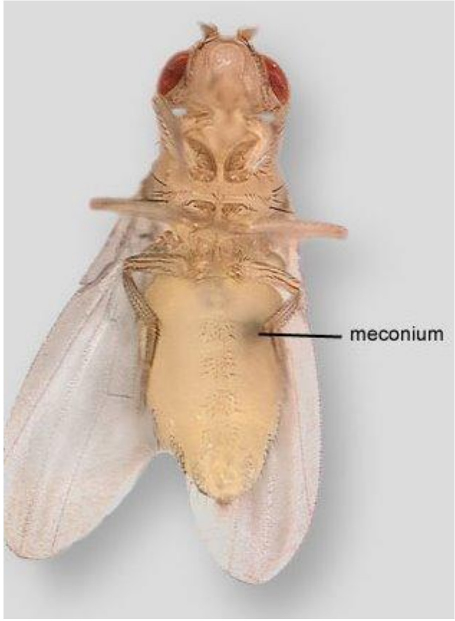

Female virgin fly - note the light pigmentation, the spot of meconium, the kind-of-inflated-looking abdomen and the opaqueness of the wings.

Imagefrom:http://theyorf.blogspot.com/2009/02/drosophila-virgins-balancers-and-jump.html

Balancer chromosomes

Balancer chromosomes have 3 major characteristics:

1.) They don't recombine with chromosomal homologues because they have multiple inversions that make synapsis (the pairing of homologous chromosomes during meiosis) and crossing over (a recombination event between homologous chromosomes) very unlikely, if not impossible.

2. They bear ***D**ominant* markers that show unambiguously if a fly carries the balancer or not. So you can genotype a fly by “looking at” it. No PCR and sequencing required. This visibility is what makes *Drosophila* genetics fast and cheap. Balancers also carry recessive alleles such as *e* for *ebony*, a coat color marker located on chromosome III that shows up when a fly has inherited both copies of *ebony* as non-functional alleles.

3. They are lethal when homozygous because, in most instances, dominant alleles are lethal when homozygous. There are exceptions to that rule; one of them is *Bar*, a dominant marker for an X-chromosome balancer.

It is the combination of these three properties that make balancer chromosomes very handy tools for genetics. If a desired mutation is generated (or, in your case, a novel P-element insertion), it will be “stored” over a balancer as a stable stock. The stock cannot take on a genotype homozygous for the balancer, so the newly generated chromosome won’t get lost. As recombination is excluded, the geneticist also always knows on which chromosomal homolog this mutation is, even if it is not marked by any other dominant or recessive marker. Lastly, if you set up a F_1 cross with a balanced stock, you know that due to the segregation pattern of chromosomal homologs, every fly in the F_1 that does *not* show the dominant markers of the balancer must carry the other (“your”) chromosomal ortholog. You can call that “genotyping by absence”.

Some Common Balancer Chromosomes (Bloomington Balancer Website) -

Name	Stock number	Balancer for chromosome	Dominant allele(s)	Lethal allele	Other alleles to know about…
CyO	3628	II	Cy - curly wings	Cy is lethal when homozygous	no
TM6B (TM6B, Tb[1])	7108	III	Hu - humeral<br>Tb - tubby	Hu and Tb are lethal when homozygous	e - ebony body color
TM2	7108	III	Ubx[130] - changes halteres to wings - halteres look larger with wing hairs on the surface	Ubx[130] is lethal when homozygous	no

Just a bit more on balancer chromosomes…balancers can align and cross over during meiosis with their non-inverted homolog in a variety of ways. Look at the picture below for two examples:

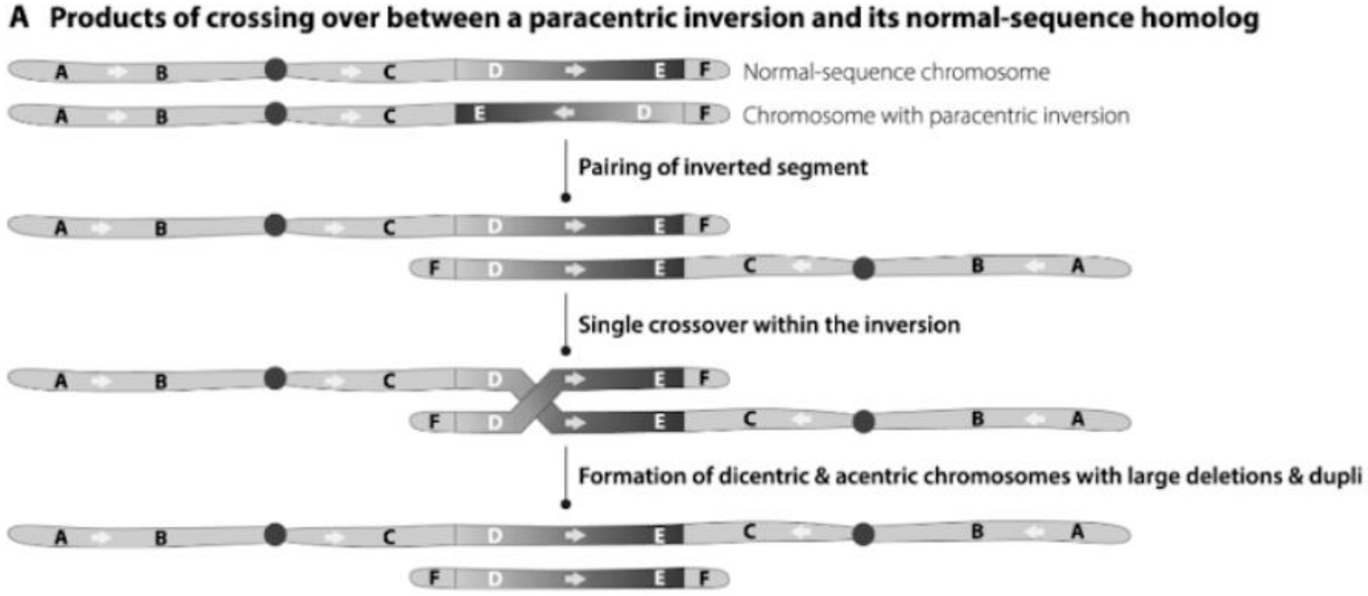

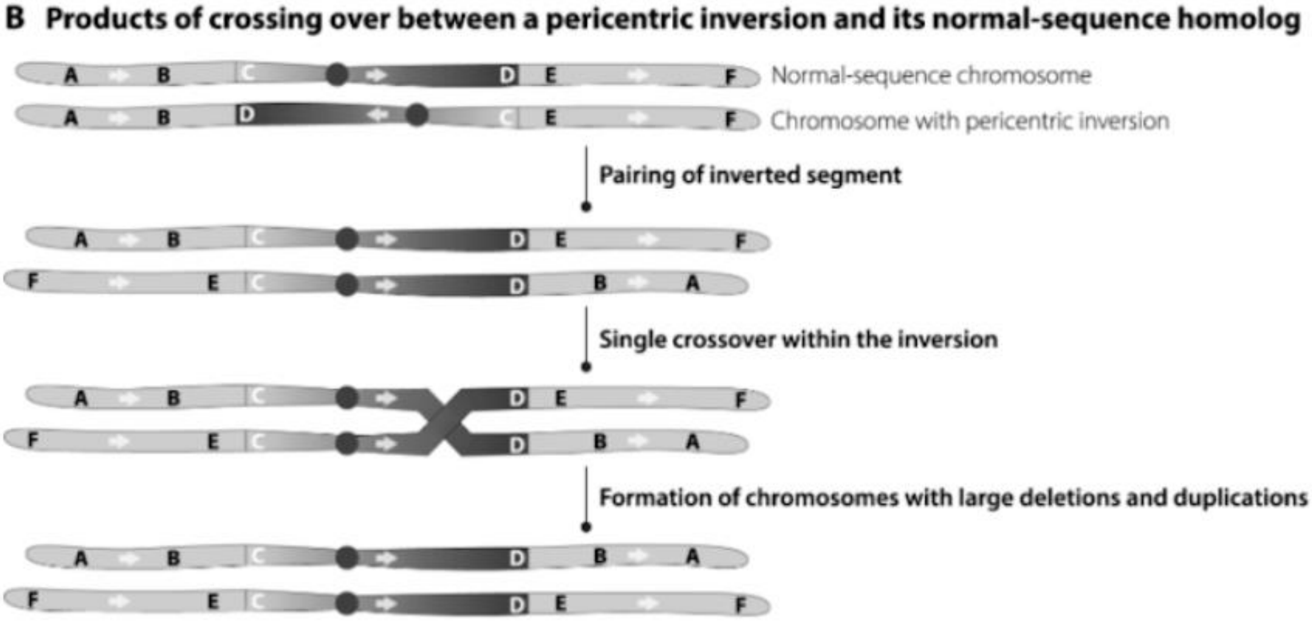

Gametes with chromosomes carrying major chromosomal insertions or deletions will not result in viable offspring. Below is a picture of the CyO balancer and a wild version of the second chromosome with three possible insertion locations for a P element. The right facing arrows on CyO are regions which align with the non-inverted homolog and the left facing arrows are inverted regions. The numbers shown on the CyO chromosome indicate the regions of the chromosome - the numbers on the wild type chromosome increase from left to right. For which option (1,2 or 3) is CyO the LEAST effective balancer chromosome and why?

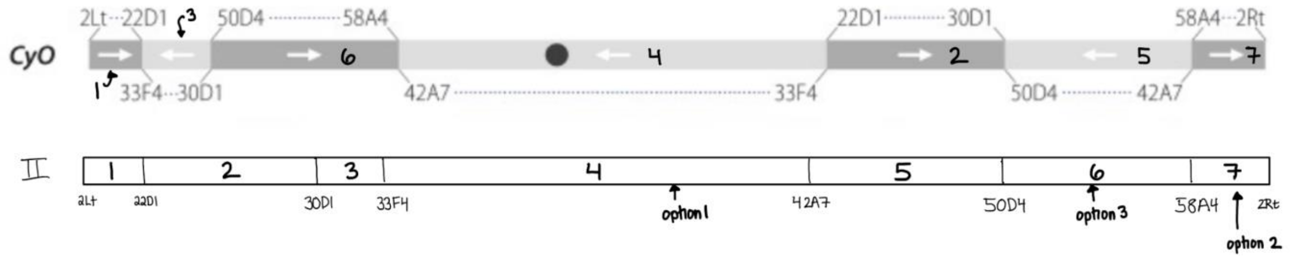
CyO
2Lt···22D1
50D4···58A4
22D1···30D1
58A4···2Rt
33F4···30D1
42A7···33F4
50D4···42A7
1
2
3
4
5
6
7
II
2Lt
22D1
30D1
33F4
42A7
50D4
58A4
2Rt
option 1
option 2
option 3

The meaning of a stable fly stock

So what does it mean “stable fly stock”? It means that males and females in one vial give rise to offspring of the same (or very similar, see below) genotype as the parental genotype. So that means after a stock is generated, you can put it on a shelf, and after several generations, the genotype has not changed and you know what you are dealing with.

Maybe this becomes clearer considering a non-stable fly strain. Let’s assume P{w^+,*StanEx1*} is just “floating” over +. The genotype would be w^-; P{w^+,*StanEx*}/+. There are plenty of people who keep their stocks like this, and as you can pick out the flies carrying the P-element by their red eyes, everything should be fine, right? Only partially so. For one thing, having an unmarked *wild-type* chromosome in your follow-up crosses can be a big drag. Furthermore, some insertions confer a disadvantage to their carriers. These flies have less offspring than their counterparts in the same vial that do not carry the insertion. Over the course of several generations, the P-element carrying flies will be simply out-competed, and you end up with a stock of w^- flies. The transposon “got lost”. Not good.

How to write transgene genotypes

When a synthetic DNA gene designed by molecular biologists is introduced to the genome, we call this foreign gene a transgene. There are many ways to introduce a transgene into the fly genome. One of the ways is to borrow certain DNA sequences of transposable elements (transposons) that can insert themselves into genomic locations.

Let's start with *P{GawB}* transgene (https://flybase.org/reports/FBtp0000352.html) that was made by Brand and Perrimon (1993). First, molecular construction of the transgene; a yeast gene called *GAL4* (our payload gene) and a synthetic version of the fly *white* gene called 'mW.hs' (a marker gene) were inserted in a plasmid DNA backbone called pBluescript and named this plasmid 'GawB'. They also attached a pair of small DNA sequences found in a transposable element called *P* so that your synthetic sequence flanked by two *P* element end sequences (now called P{GawB}) can be inserted into the fly genome. Second, generation of the transgenic line; they co-injected P{GawB} and *P* transposase into a *white* mutant fly embryos so if a transgene is inserted in the germline of injected animals, this special progeny from injected animal can be identified by red-eye expression of GawB in otherwise white-eyed progeny.

Since *P* transposase can insert P{GawB} in random locations in the genome, Brand and Perrimon had to come up with a naming system for the same transgene (FBtp) in different locations (Fbti). Here are examples of these names (https://flybase.org/hitlist/FBtp0000352/to/FBti#)
P{GawB}AB1
P{GawB}389
P{GawB}C23
…
If P{GawB} was inserted near a known gene, then they added the gene name with the allele name after P{GawB}. Here are examples
P{GawB}ptc[559.1] or P{GawB}$ptc^{559.1}$
P{GawB}tey[5053A] or P{GawB}tey^{5053A}
P{GawB}Bx[MS1096] or P{GawB}Bx^{MS1096}
…
But these insertions may come in different mutant background, and also need a marker method to identify the transgene. How do we denote if the 'transgene' that can be identified by 'marker' was introduced using '*P* element sequences' into a certain fly genome or genetic background? We use a genotype format of P{marker=transgene} in conjunction with mutant genes and their allele names in the genome. When Brand and Perrimon introduced the synthetic transgene 'GawB' that contains a *white* allele called w[+mW.hs] using P element sequences into w[1118] genetic mutant genome, we write the fly strain genotype as w[1118]; P{w[+mW.hs]=GawB}

Here are the genotypes of the first few strains that they started to share through Bloomington Drosophila Stock Center (BDSC; https://bdsc.indiana.edu/).

458 P{w[+mW.hs]=GawB}elav[C155]
1734 w[*]; P{w[+mW.hs]=GawB}h[1J3]
1747 y[1] w[*]; P{w[+mW.hs]=GawB}71B
1767 w[*]; P{w[+mW.hs]=GawB}how[24B]
1774 w[*]; P{w[+mW.hs]=GawB}69B
8860 w[1118] P{w[+mW.hs]=GawB}Bx[MS1096]
…

Some Hard Earned Suggestions for Fly Work

In no particular order, here are some things we have learned the hard way about doing fly work. Read, think, absorb so you do not make all the mistakes that we did!

1. Always use virgin females in crosses. If you are not absolutely sure that a female is a virgin, do not use her. Flies become sexually mature about 8 to 10 hours after eclosion (emerging from pupal case). You can distinguish females which are too young to be sexually mature by the presence of a meconium and a pale body. If these flies are separated from males while still sexually immature, then you know they are virgin flies even after they become sexually mature.

2. Label every vial. Things to include on the label might be - your initials, parents, date the cross was made, letter to indicate flip, date offspring first appeared, expiration date of vial.

3. Always think about the date on which you need to stop collecting offspring flies from a cross. This date is about 10 days after the first time a sexually mature female and male were present in the vial at the same time. Think of this as the expiration date of the vial. After the expiration date you no longer have certainty about the parentage of the offspring flies.

4. One trick to making lots of offspring from a single cross is to place the parents onto new food every few days. This is called flipping your flies. We usually use a letter to indicate the flip - A for the original vial, B for the second vial and so on. It is critical that you can identify the lineage of flipped vials. You might label a cross #1 and so the labels would be 1A, 1B, 1C as you flip this cross. The replicate cross would be 2A, 2B, 2C. Keeping the replicates independent and identifiable means that if you discover a problem with the cross as you are flipping from C to D, you can discard the entire lineage of vials for one replicate and keep the other replicate.

5. Flies do not like dry food, so be sure to keep the food moist. But wait, flies do not like wet food, so be sure not to over moisten!

6. Create habits so you know (with absolute certainty) which vial the flies you are looking at came from. We suggest ONLY removing one vial from your fly box at a time. Set this vial next to your microscope while you are observing the flies. It is very sad to discard a fabulous fly because you are not absolutely certain that you know from which vial it came.

7. Anesthetize your flies with a gentle hand. If you blast them into the fly food at a million miles an hour using the CO_2 nozzle, guess what, they are not going to survive.

8. Eclosion Date - This is (almost) exactly what it sounds like...the date of the first eclosing flies in the bottle or vial. The purpose of recording this date is to be sure we know the date on which new eggs from sibling (or parent sibling if all parents have not been removed) matings could

have been laid in this container. So...more specifically the eclosion date is really the date on which you had sexually mature male and female flies in the container at the same time. **It is critical that you check your vials regularly and record the eclosion date!** To ensure that we never accidentally use flies in subsequent crosses which resulted from a sibling sibling or parent sibling cross we will discard all containers ten days after the eclosion date. This is a conservative estimate of the time required to develop from a fertilized egg to an adult fly.

Some Questions…

1. Do you notice anything surprising about the *Drosophila* chromosomes in comparison to our own?

2. In the following statements, circle the words that refer to genes. ~~Cross out~~ the words that apply to proteins.

   a. A mutation in *cheapdate* leads to a malfunctioning Cheapdate, leading to an increased susceptibility to alcohol.

   b. Transposase activity is lower in certain tissues.

   c. Expression of *transposase* is decreased in certain cells.

3. Look at the genotypes listed below. Circle the genotypes of the male flies. Explain why you know the answer to this question.

   $w^-/w^-;L/Cy$ $w^-/Y;L/Cy$

4. Label the following genotypes as homozygous or heterozygous.

   a. w^+/w^+

   b. w^-/w^-

   c. w^-/ w^+

   d. e^-

5. List the phenotype (including sex!) of the following genotypes:

   a. +;Cy/+;+

b. w^-; Cy/L; Hu,e/e

c. w/Y; Cy/L; Sb/ Hu,Tb,e

d. +; L/+; Sb/+

6. List all of the possible genotypes associated with flies of the following phenotypes. Only include alleles mentioned in the prompt.

    a. Red eyed, Ebony colored, wild type wing female

    b. White eyed, wild type (honey) colored, curly winged male with humerals

    c. White eyed, lobe eyed, wild type (honey) colored, wild type wing female with stubble.

7. You observe that a fly with the genotype y, w/y, w; +/+; e/e is ebony. What interesting tidbit have you learned?

8. Write out a cross to demonstrate the meaning of these sentences to yourself…

> "If a desired mutation is generated (or, in your case, a novel P-element insertion), it will be "stored" over a balancer as a stable stock. The stock cannot take on a genotype homozygous for the balancer, so the newly generated chromosome won't get lost."

What is a binary expression system, which two are we discussing in this course and why is it helpful to have more than one?

Here are some resources to explore these questions…

- What is a binary expression system?
- What are the two binary expression systems we will discuss in this course?
- Why is it helpful to researchers to have more than one binary expression system?
    - GRASP (read just the abstract)
    - Another way researchers use split GFP (again just look at the abstract and figure 1)
    - And another explanation of this idea

In this course we are going to convert existing Gal4 driver lines into LexA driver lines. Explain in your own words what this actually means and why the product of our work will be useful for researchers (what will it allow them to do that they cannot do now)?

CRISPR

A Message from the Authors

Dear Future Curious Biology Scholars,

Be warned: the contents of this section will blow your mind. In this manual, we discuss the origins of CRISPR, how it works in bacteria, how it works for researchers, and the specifics of each stage of the process. The manual is dotted with questions to probe your understanding and to spark fruitful and meaningful discussions about CRISPR. In the very back of this section you will find a glossary that explains all of the abbreviations and names that biologists use on the day-to-day.

When you finish reading, you will be a walking CRISPR encyclopedia. You won’t be able to resist sharing how homology directed repair is key to creating our dream sperm to friends and family.

Best,
Spring 2023 Exeter Bio670 Students

Section 1: The Origins

Life for *Escherichia coli* (*E. coli*) is fairly mundane – that is, until the cell comes under fierce attack. Bacteriophages, or viruses that infect bacteria such as the *E. coli*, land on the surface of the bacterial cells with armies of invaders stored within their bulbous heads. These viral soldiers are composed of DNA (or RNA) that is injected through the cell wall (1). Hijacking the production systems within the cell, the viral DNA begins to get transcribed and translated by the bacterial RNA polymerase and ribosomes (2 & 3). When everything goes according to plan, the virus multiplies within the cell until a command is issued to lyse the cell, exploding the viral particles back out into the environment on the prowl for new victims (4). Unfortunately for the bacteriophage and fortunately for the *E. coli*, things do not always go to plan.

These figures show the successful attack of a bacteriophage on a bacterial cell (read left to right, top to bottom - numbered 1, 2, 3, 4).

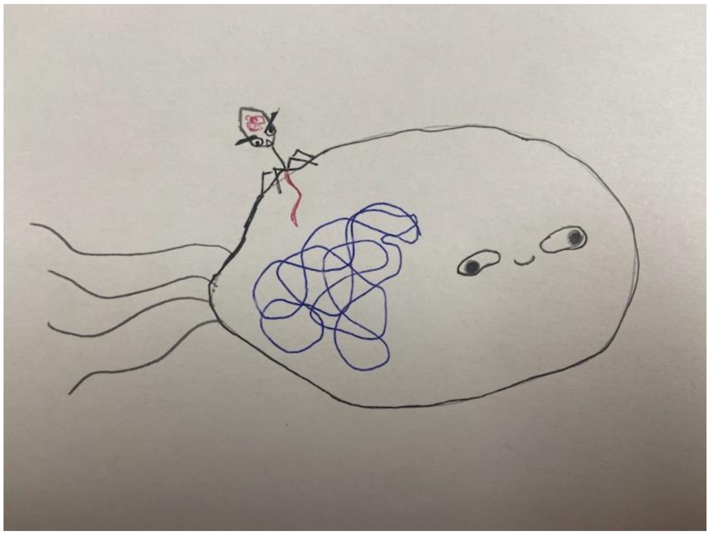

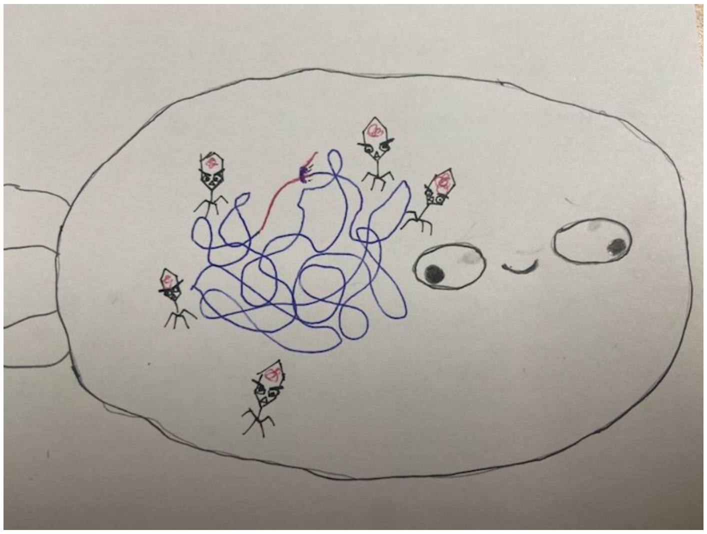

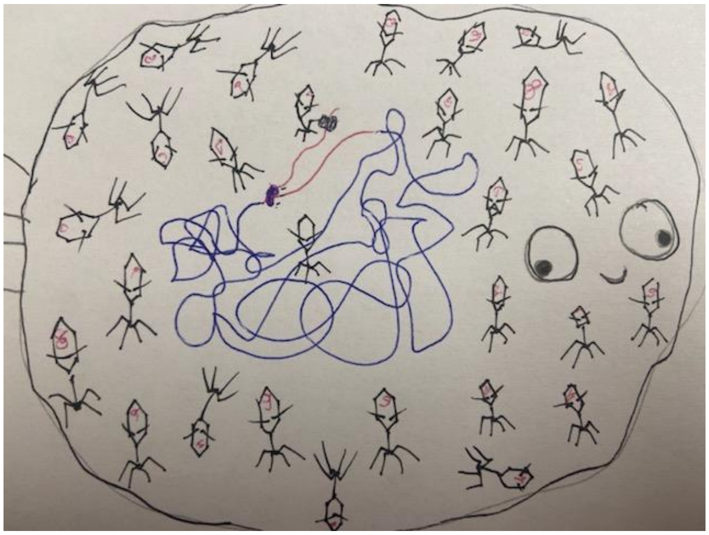

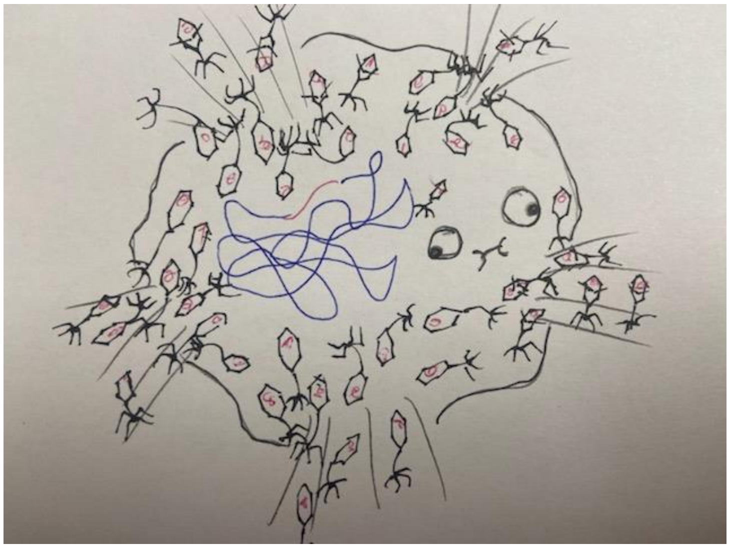

Within the *E. coli* cell lies a defense system perfectly designed to target the viral DNA before the virus multiples too much. When the bacteriophage injects its DNA into *E. coli*, various protein complexes go to work. Bacteria store a memory of past viral infections, which uses bits of chopped up viral DNA taken from each victory against the bacteriophage. The *E. coli* uses these chunks of stored viral DNA to identify the viral DNA in future attacks. Once the complexes have identified the foreign sequence, they cut the genome, forming a double stranded break in the DNA. Once the viral DNA has been chopped up, DNases, enzymes which break apart DNA molecules, destroy the pieces of viral DNA. With that, the *E. coli* has successfully defeated its enemy.[1]

These figures show a basic visual narrative of a defeated attack of a bacteriophage on a bacterial cell. Labels

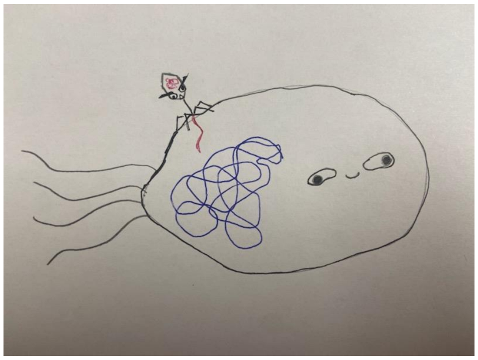

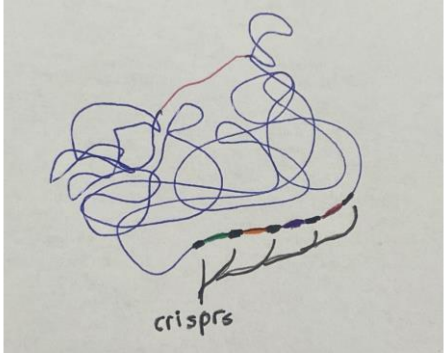

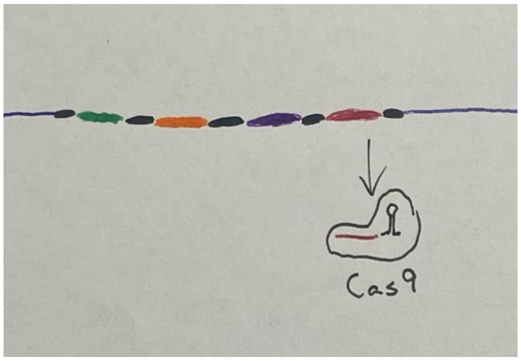

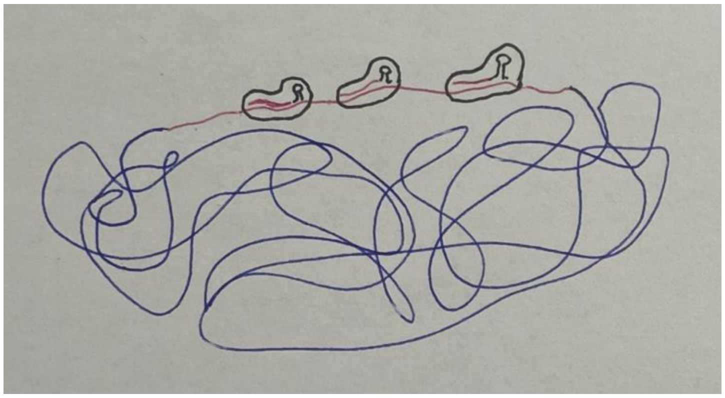

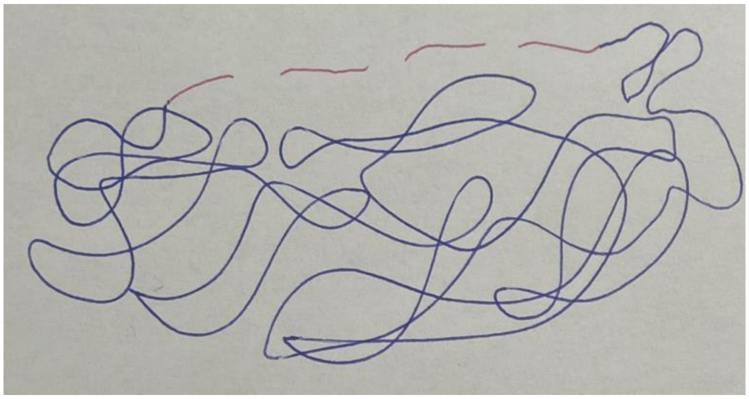

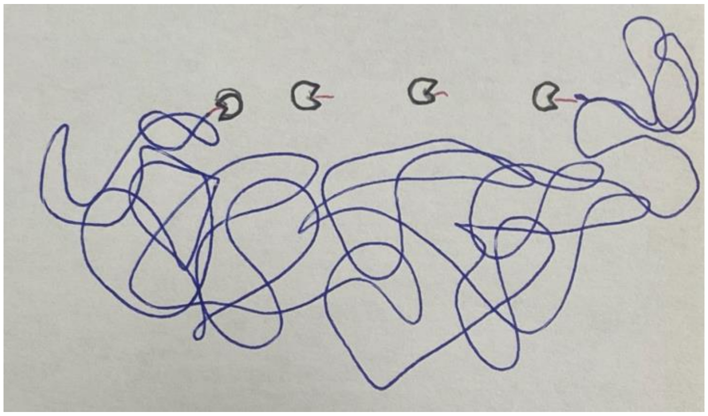

and descriptions will become more apparent and understood as you follow along this chapter.

Questions: Why do you think we chose to tell you this story? How do you think this story plays a role in CRISPR and its uses in the modern day?

---

[1] This video was referenced in the writing of this section: How CRISPR Works as a Bacterial Immune System

Section 2: The Discovery

Before the battle between bacteria and bacteriophages was known to scientists, researchers had been paying close attention to CRISPRs, or Clustered Regularly Interspaced Short Palindromic Repeats. A palindrome is a sequence that appears the same when read forwards as it does backwards. These repeats were found within bacterial and archaeal genomes. CRISPRs were noticed in *E. coli* for the first time in the 1990s, and later were found in other species of bacteria and archaea. However, at that time, knowledge of the genome, especially of mobile genetic elements, was very limited and remained this way until the early 2000s.

Francisco Mojica noticed that the DNA sequences between the palindromic repeats contained copies of fragments of DNA from bacteriophages, archaeal viruses, and plasmids. This gave him and other scientists a clue that these CRISPRs may play a role in the immune defense system of a bacterial cell. Researchers also noted that bacteriophages did not successfully infect organisms who contained their homologous sequence within their bacterial genome. By the time of Mojica's research, the entire genomes of organisms were beginning to be sequenced – and many bacteria and archaea had CRISPRs. Furthermore, scientists had begun to notice a highly conserved region near the CRISPRs that contained the coding sequence for CRISPR-associated proteins (Cas proteins). Roughly, they knew that there were several Cas genes. The genes *cas1* and *cas2* encode proteins that integrate viral DNA into the genome. Cas3, Cas9 and Cas10 are proteins made from Cas-genes that code for different molecular scissors. Cas9 is the protein that will be used in this system.[2]

Question: Using ideas from the story above, how do you suspect the proteins and the homologous viral DNA within the bacterial genome work together?

[2] Section 1 until footnote adapted from "History of CRISPR-Cas from Encounter with a Mysterious Repeated Sequence to Genome Editing Technology - PMC".

Section 3: The CRISPR-Cas9 Mechanism

Our procedure aims to convert a particular *D. melanogaster* driver line from the GAL4/UAS to LexA/LexAOp binary expression system. To do so, we must use CRISPR-Cas9 to induce a cut in the GAL4 insertion site in the original driver line. How exactly does CRISPR work in bacteria? And how is the process complicated when transferred into fruit flies? This section will first give a brief overview of the CRISPR mechanism, and before going into each of its three steps in more depth, will discuss an important motif known as the PAM sequence. It will finish with a discussion of how CRISPR is adapted for gene editing procedures.

CRISPR in Bacteria

Watch this video from the 2:30 to 5:30 minute mark for a basic overview of the CRISPR mechanism in bacteria. Then answer the following questions:

1. Based on the diagram below – and the video you just watched – can you come up with a basic three-step process summarizing the CRISPR mechanism?

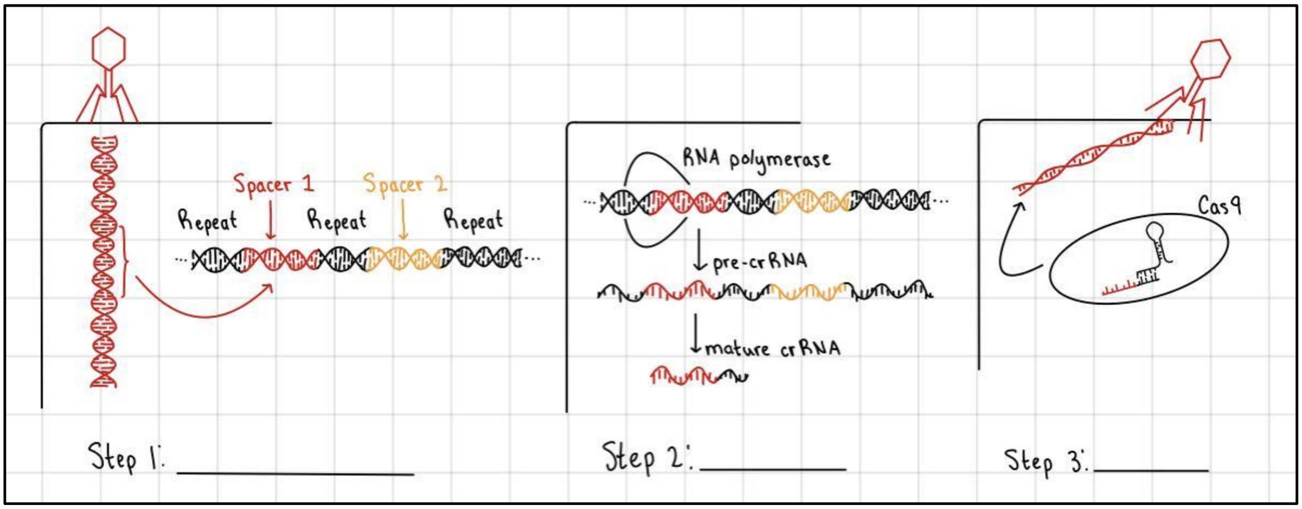

2. CRISPR stands for Clustered Regularly Interspaced Short Palindromic Repeats. Explain why each word of this acronym makes sense.

3. The following sequence was given in the video as an example of a repeat sequence found in CRISPR arrays: **5’CAATAACTTTTCAAAAGTTATTG3’**. It is not entirely obvious, at first, that this sequence is a palindrome. Explain why, and then demonstrate that it is.

4. A bacterium has developed immunity to a certain bacteriophage by incorporating a small portion of the viral genome into its CRISPR array. A week later, the bacterium is again infected by that same viral strain, but unexpectedly, dies shortly thereafter. Come up with at least 2 explanations for why this might have happened.

PAM Sequences

Watch this video on a particular sequence – the protospacer adjacent motif (PAM) – involved in CRISPR-Cas9. Then answer the following questions:

1. In your own words, explain why a gRNA-Cas9 complex doesn't cut the CRISPR array that created it.

2. A bacterium uses its CRISPR-Cas9 defense system to induce a DSB between the two highlighted nucleotides in the following sequences: **5'ATCGGAAATCCAGTGG 3'**. Based on the table below, from which bacterial strain did this example come from? Assume for the purposes of this question that the cut site is always 4 nucleotides upstream (to the left) of the PAM sequence.

Bacterial species	PAM sequence	References
Streptococcus pyogens (Sp)	NGG	(Jinek et al., 2012)
S. solfataricus I-A1	CCN	(Manica et al., 2011)
S. solfataricus I-A2	TCN	(Gudbergsdottir et al., 2011; Lillestøl et al., 2009)
Neisseria meningitidis (Nm)	NNNNGATT	(Hou et al., 2013)
Streptococcus thermophiles (St) Type II	NGGNG<br>NNAGAAW	(Deveau et al., 2008; Horvath and Barrangou, 2010)

3. A bacteriophage with the following DNA sequence infects a *Streptococcus pyogenes* cell: **5'AGCTGTCGAAACGTTGAGGC3'**. The cell incorporates the highlighted region as a new spacer into its CRISPR array. A week later, that same bacterium is infected by a mutated form of that viral strain (see highlight for point mutation): **5'AGCTGTCGAAACGTTGACGC3'**. Explain the implications of this mutation for the *S. pyogenes* cell. Will CRISPR succeed? Explain your reasoning.

4. *(Continuation)* Imagine instead that the bacterium was infected by a virus with a different mutation: **5'AGCTGTCGAAACGTTGTGG3'**. What would the implications of this mutation be for the *S. pyogenes* cell? Explain your reasoning.

5. In the context of evolution, why might it make sense that PAM sequences are shorter (2-6 nucleotides) rather than longer (say, 15 or 30 nucleotides)?

Acquisition

Bacteria must be able to "acquire" a segment of DNA for their CRISPR array when infected by a novel bacteriophage. This process is carried out by two Cas proteins: *cas1* and *cas2*. These are endonucleases – they create breaks in a DNA strand. When a novel viral strain invades a bacterium, the *cas1* and *cas2* proteins first look for a PAM sequence in the viral DNA. After that, they induce double-stranded breaks a few nucleotides upstream of the PAM sequence around a DNA segment known as the protospacer – the protospacer is cut out of the viral DNA. "Proto-" is a prefix that means "the earliest form of," so the protospacer is the segment of viral DNA before it is inserted into the CRISPR array as a spacer. The spacer is joined to the front of the CRISPR array, and acquisition is complete.

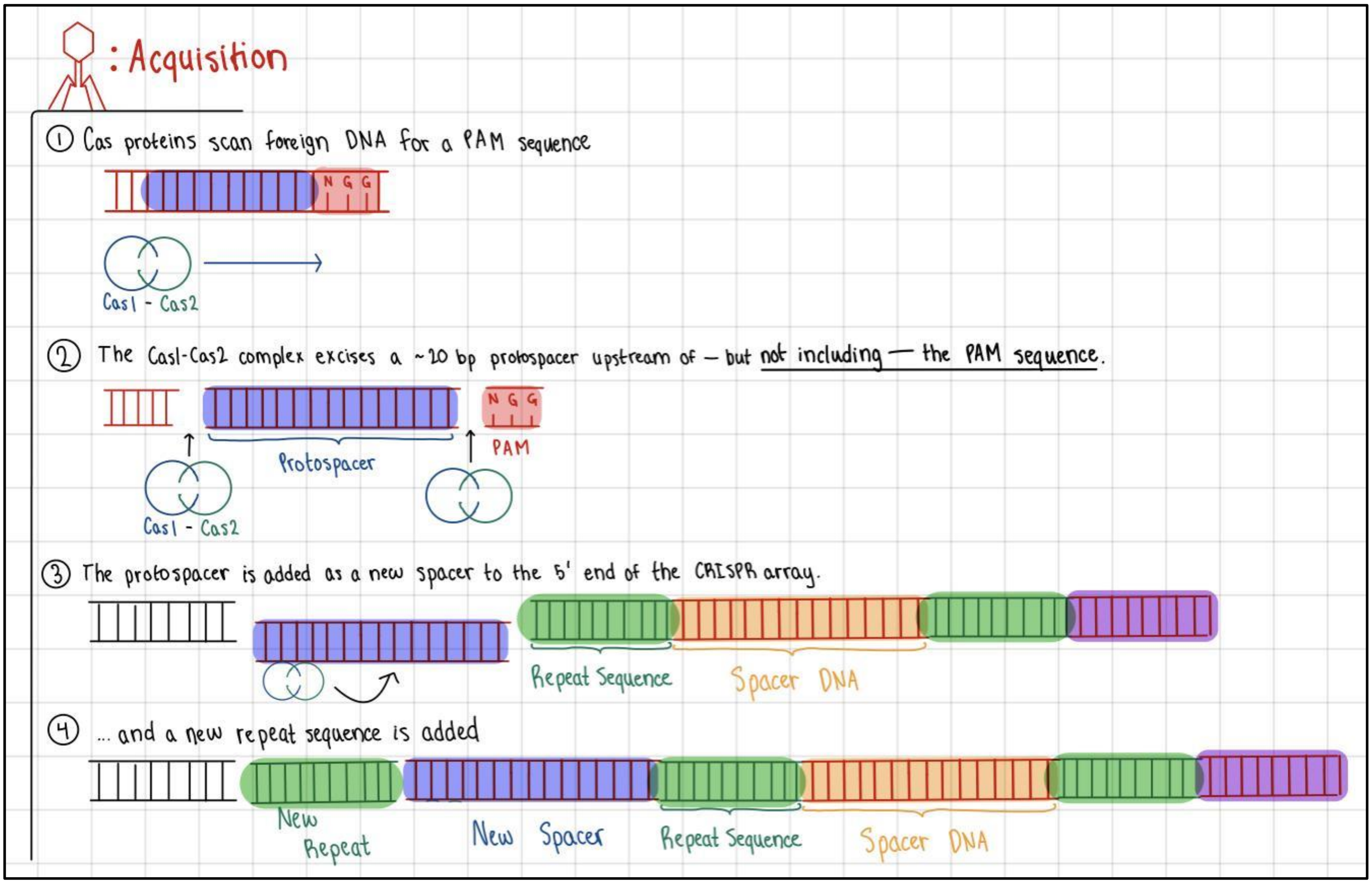

1. As seen in the diagram to the right, the PAM sequence isn't included in the protospacer. Why is it *crucial* that the PAM sequence is not included in the CRISPR array?

2. If bacteriophages are deadly, how do bacteria ever gain immunity and survive the first infection of a novel virus?

3. Why is it that a PAM sequence is always downstream of a target sequence of DNA in a virus?

crRNA Biogenesis & tracrRNA

Watch this video from the 4:46 to 6:43 minute mark for an overview of crRNA biogenesis – pay special attention to the discussion of a molecule called tracrRNA. Then answer the following questions:

1. As seen in the diagram to the right, there is a *single* tracrRNA coding sequence in the bacterial genome. Based on that information, why does it make sense that tracrRNA binds to palindromic repeats rather than spacer DNA?

2. tracrRNA is palindromic and therefore forms a stable hairpin loop when transcribed. That loop serves as a “handle” for the Cas9 protein to latch onto.[3] Based on that information, explain why it is *crucial* to the CRISPR mechanism that tracrRNA can bind to the repeat sequence (see diagram).

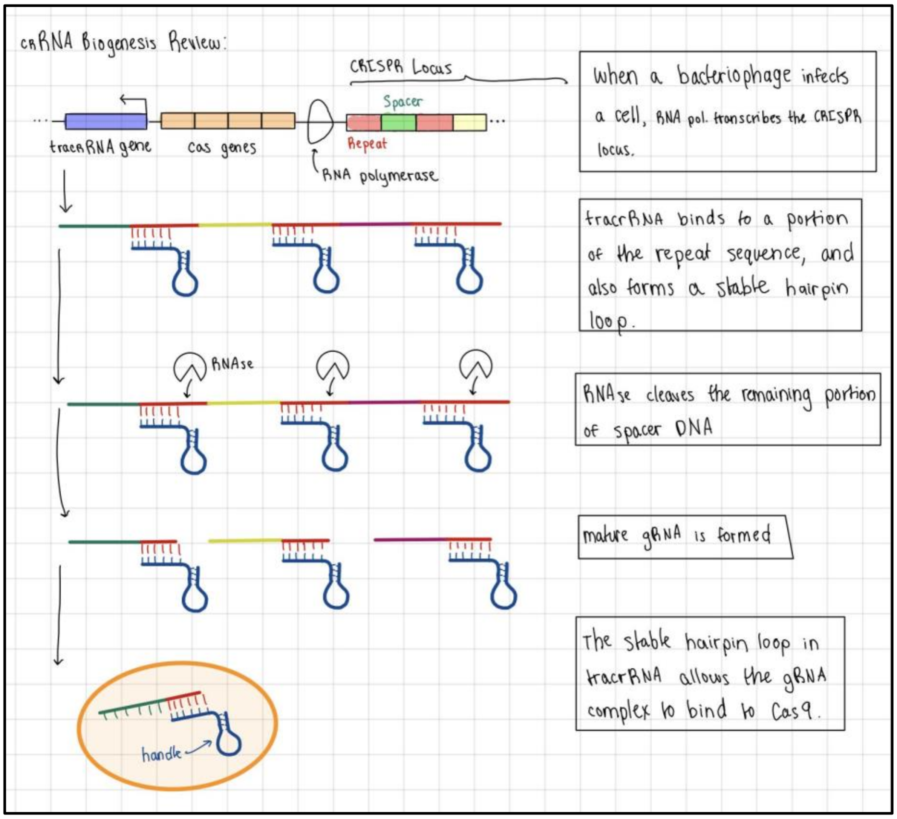

[3] https://innovativegenomics.org/glossary/tracrrna/

Interference

If a bacteria is infected by the same viral strain more than once, the bacteria can use its CRISPR-Cas9 defense system to interfere with viral DNA – essentially stopping the bacteriophage infection.

1. PAM sequences are not included in the CRISPR locus and therefore prevent a gRNA-Cas9 complex from cutting the sequence that created it. Based on the diagram below, what is a *second* reason PAM sequences are helpful to the CRISPR/Cas9 mechanism? (Remember that the Cas9 protein *itself*, and not some complementary RNA molecule, recognizes the PAM sequence.)

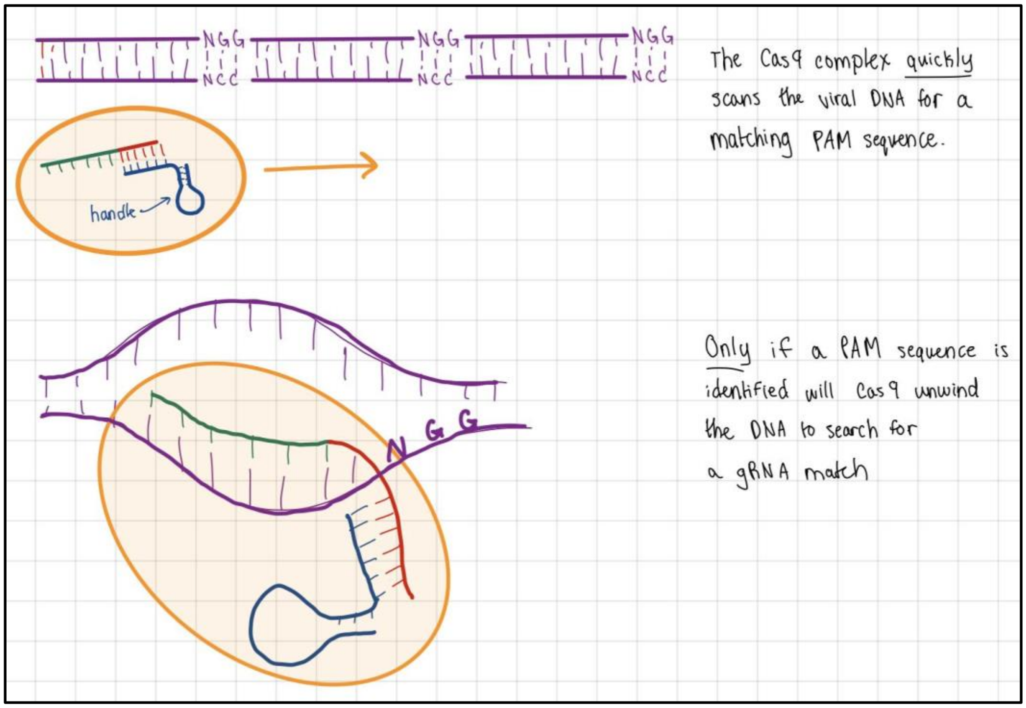

Once the Cas9 complex identifies the correct sequence in the foreign DNA, the Cas9 protein unravels the DNA at that spot, creating an R loop. The Cas9 protein then creates a break in each of the two DNA strands. The viral DNA is cut up, and the virus dies.

CRISPR in Gene Editing

You now understand how CRISPR functions as an adaptive immune system in bacteria – how it cuts DNA at a specified sequence. This tool has been leveraged in research labs as a tool for gene editing, which involves cutting DNA at a precise location (see why in section 4); that includes our HACK procedure. There are a number of challenges implicated in using the mechanism as a tool – here we will outline only a few.

Watch this video from the 2:12 to 3:12 minute mark. Then answer the following questions to understand more about the use of CRISPR in gene editing:

1. A research scientist wants to make a cut *anywhere* within the following target DNA sequence: **5'ATTACAAATATTATAAGAATTAATATTACGGCGCGGAATGCATAGTTGCGAAAATCGGTTTTAGATCAGTATAT3'**. They will be using a Cas9 protein found in *S. pyogenes.* To gauge potential cut sites, highlight all possible PAM sequences in green.

2. (*Continuation)* The geneticist decides to make a cut upstream of the highlighted PAM site, and therefore engineers a gRNA complementary to the sequence highlighted in orange. **5'ATTACAAATATTATAAGAATTAATATTACGGCGCGGAATGCATAGTTGCGAAAATCGGTTTTAGATCAGTATAT3'**. Why are their CRISPR efforts unsuccessful?[4]

3. [5]The researcher creates a suitable crRNA-tracrRNA pair to make this cut. The two molecules are present, **separately**, in the genome (as well as Cas9). Unfortunately, CRISPR has a low success rate. Explain why.

4. *(Continuation)* The scientist then hybridizes the crRNA and tracrRNA with a short segment of DNA called a linker loop – see the diagram to the right.[6] The CRISPR success rate increases. Why did this solve the problem?

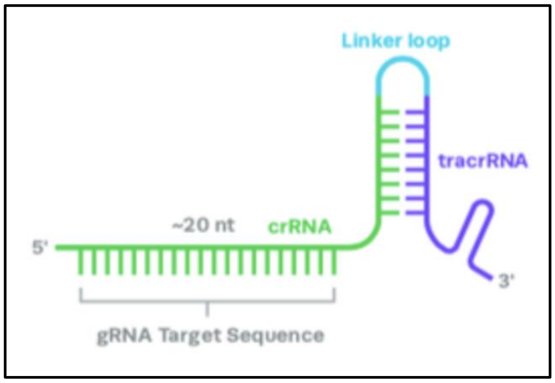

5. In our HACK procedure – and many gene editing procedures – the DNA template for gRNA is inserted after the "U6" promoter. Take a look at the following excerpt from a paper on the optimization of CRISPR/Cas9 for gene editing: "The *U6* promoter is a class III RNA polymerase III promoter that has been frequently used to drive high expression levels of small RNAs in plants and animals and has been the preferred choice to drive sgRNA expression in CRISPR/Cas9 vectors. In addition, the *U6* promoter has a highly conserved transcription start site starting with a guanine nucleotide, which helps to improve the homogeneity of the transcribed sgRNA molecule and reduce off-

[4] Low GC content → binds
[5] Our goal here was to explain the purpose of combining tracrRNA + crRNA into a single sgRNA (also linker loops)
[6] What's the difference between crRNA, tracrRNA, and gRNA in CRISPR/Cas9? - Quora

target effects."[7] In your own words, explain why we regulate the transcription of sgRNA under the U6 promoter.

[7] https://www.ncbi.nlm.nih.gov/pmc/articles/PMC6169012/

Section 4: Repair Methods after DSBs

In order for gene editing to take place, the broken DNA must be repaired in a way so that new DNA can be incorporated into the genome at the same time that the broken ends are joined. There are two major repair mechanisms that the cell uses to complete the DNA after the Cas9-induced double-stranded break: homology-directed repair (HDR) and nonhomologous end joining (NHEJ). While each repair mechanism has its own advantages and disadvantages, HDR is the only mechanism that allows for gene knock-ins, or copying of specific DNA sequences through the repair of double-stranded breaks.

Nonhomologous End Joining

Nonhomologous end joining, or NHEJ, is the process by which the broken ends of the genome that have been cut by the Cas9 protein are repaired and joined together. NHEJ occurs in three main steps: recognition, recruitment, and ligation (and detachment).[8]

Watch this video on the NHEJ process in full to understand the following key steps.

<u>Deep Dive Questions:</u>

Recognition: What molecules *recognize* the broken ends of the double-stranded DNA? Why is this recognition necessary?
The first step of NHEJ occurs when nucleases help to break down around 10 nucleotides from the broken ends of the DNA.[9] Then, the molecule which recognizes the broken ends of the DNA is known as Ku70/80, a heterodimer protein, meaning that it contains two polypeptide subunits. The Ku70/80 has a high affinity for the broken DNA ends and a high abundance within the nucleus.[10] Therefore, the protein recognizes the broken ends of the two DNA strands and serves as protection against any further nuclease activity, in which enzymes break down the chains of nucleotides as they are recognized as foreign or "problem" DNA.

Recruitment: What proteins are *recruited* to the broken ends and how do these molecules aid in the later ligation step?
The Ku70/80 protein then serves as the scaffolding for various other molecules, and recruits these accessory proteins that will eventually aid in modifying the broken ends so that ligation can be completed. First, Ku 70/80 serves as the binding site for the DNA-dependent protein kinase catalytic subunit (DNA-PKcs), which together with the Ku form the DNA-PK holoenzyme, which controls interactions between broken DNA ends. The heterodimer then recruits nucleases (such as Artemis), polymerases, and the DNA ligase 4 complex. These accessory factors also bind to the complex, hold and modify the pair of DNA ends together, and form what is known as the "paired end" complex.[11]

---

[8] https://blog.addgene.org/crispr-101-non-homologous-end-joining
[9] https://www.youtube.com/watch?v=31stiofJjYw via Oxford University Press
[10] https://www.ncbi.nlm.nih.gov/pmc/articles/PMC8073936/
[11] https://www.ncbi.nlm.nih.gov/pmc/articles/PMC8073936/

Ligation: How are the two strands ultimately *joined* together?
Finally, once all accessory proteins have been recruited and the holoenzyme complex has dissociated, ligation can occur. This occurs via another protein complex, the XRCC4/Lig4 complex.[12] Ultimately, scaffolding proteins and proteins which perform enzymatic functions all contribute to the ligation, or connection, of the two broken DNA. After ligation, these protein complexes dissociate, and repair of the DSB is complete. However, due to the nuclease and other enzymatic activity at the ends of the broken DNA, the new strand is not repaired to its original sequences, and there is also greater possibility for insertion and deletion events.[13]

The following diagram showcase the three main stages of NHEJ and its potential outcomes:

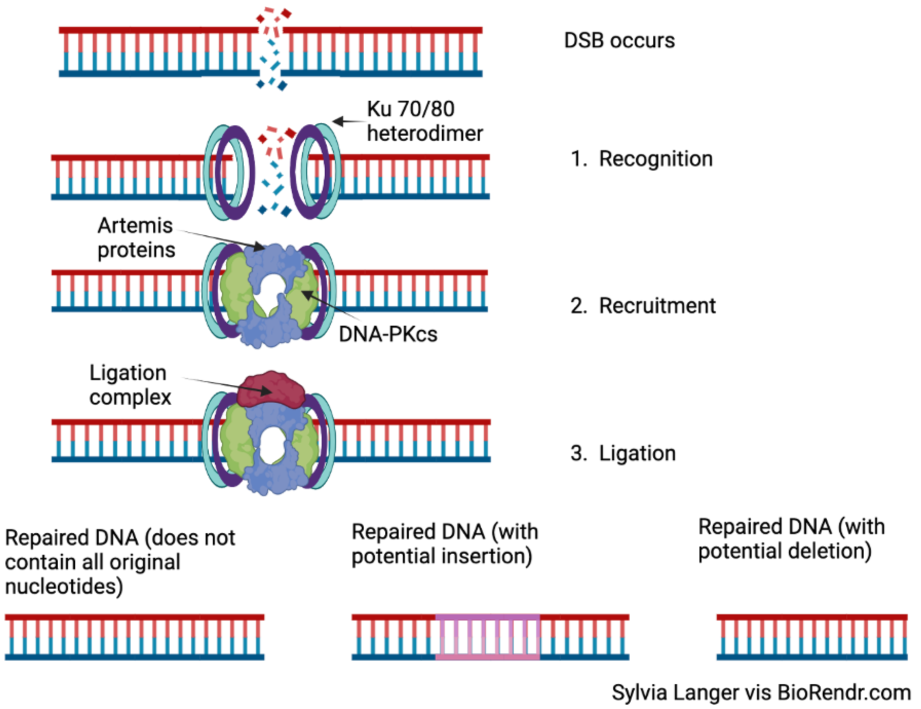

[14]

---

[12] https://www.ncbi.nlm.nih.gov/pmc/articles/PMC8073936/
[13] https://www.sciencedirect.com/science/article/pii/S1097276519308664#:~:text=NHEJ%20has%20the%20potential%20to,ends%20to%20allow%20their%20ligation.
[14] Sylvia Langer via app.biorendr.com

Homology-Directed Repair

HDR, or homology-directed repair, is a mechanism that cells use to repair double-stranded breaks. It uses a template strand that contains a sequence homologous to the broken strand in order to repair the DNA more accurately.

Once the double-stranded break occurs, it is recognized by protein complexes. These proteins then recruit nucleases that prepare the ends of the DSB for replication. Then, another protein searches for a sequence homologous to the broken DNA on an undamaged molecule.[15] Once this sequence is found, DNA replication can begin. The cell's replication enzymes will replicate the entire region between the sequences homologous to each end of the double-stranded break. The resulting strand will therefore be identical to the original strand. Watch this 3-minute video by Oxford University Press for an animated version of the process.

How is HDR used with CRISPR-Cas9?

While the template strand usually originates from inside the cell, scientists have used homology-directed repair to introduce new DNA sequences into an organism's genome. This is called a **gene knock-in**, and requires a donor template strand. The donor strand contains both the sequence of the gene to be knocked in and sequences homologous to the regions on either side of the cut, called **homology arms**.[16] The sequence where the new gene will be inserted is called the **target sequence**.

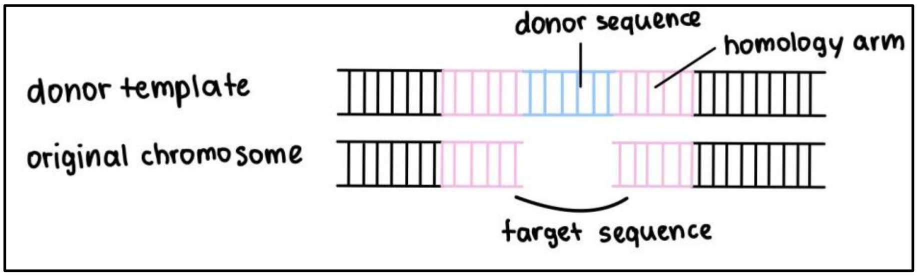

HDR Step-By-Step

In order for HDR to occur, the donor template and the original strand have to be in the same cell, on a pair of homologous chromosomes. (Note that the Cas-9 gene and the gene coding for the guide RNA also have to be present). One way to accomplish that is by crossing a fly containing a chromosome with the donor sequence with another fly which has a chromosome with the target sequence.

Scientists often insert the donor sequence and target sequence as p-elements. This enables them to insert marker genes along with it, which lets us know which flies contain the p-elements. For example, if the y+ (honey-colored) gene is inserted into the p-element with the donor sequence, we know that the flies that have honey-colored bodies all carry the element. Likewise, if we insert the w+ (red-eyed) gene into the p-element with the target sequence, we will know that all red-eyed flies carry the target sequence. In this example, we would look for flies which are red-eyed and honey-colored because we know that they carry both of the necessary p-elements.

[15] https://pubmed.ncbi.nlm.nih.gov/9697414/
[16] https://www.synthego.com/learn/crispr

Below is a diagram showing how HDR can be used for genome editing:

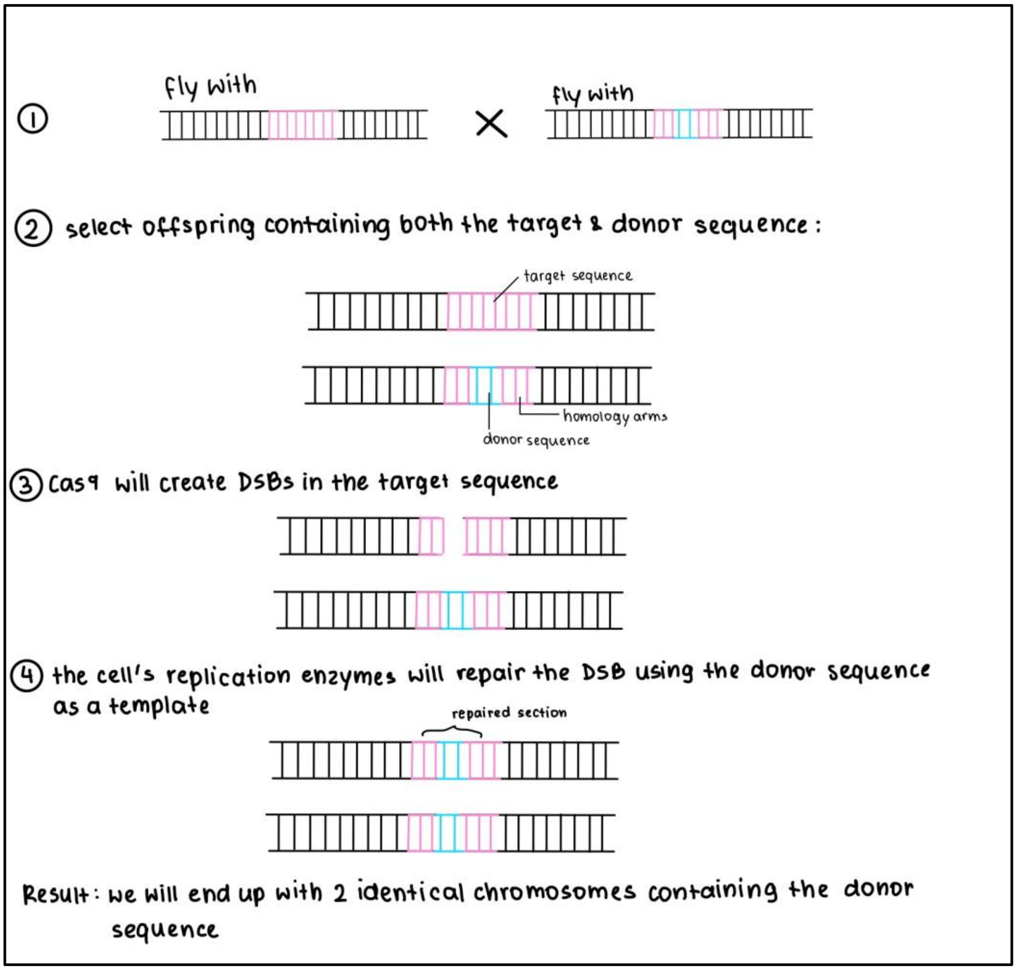

What are the common misconceptions associated with HDR?

1. A common misconception is that the target sequence is "replaced" by the donor sequence. This is false! The target sequence never gets removed– it is just broken, and the new gene is inserted in between the broken ends. Therefore, we will end up with 2 identical chromosomes, which both contain parts of the target sequence (recall the homology arms) and the gene insertion.
2. Another misconception is that the donor sequence is placed into the target sequence and removed from the original chromosome in the process. This is also false! The donor sequence remains on its original chromosome, and is simply replicated into the chromosome with the DSB.

How can we ensure that the desired repair mechanism is used?

Imagine you wanted to use CRISPR-Cas9 to insert a gene. This requires the use of HDR, so if the cell instead used NHEJ to repair the double-stranded break, the gene insertion would fail. This is complicated by the fact that NHEJ is usually the preferred repair method because it is more efficient. There are a few factors that determine which repair mechanism will be used:[17]

- The structure of the DSB: breaks with long overhangs are more frequently repaired by HDR, while "blunt" breaks are usually repaired by NHEJ.

[17] https://www.sciencedirect.com/science/article/pii/S0021925820338084

- The age of the cell: HDR is often used in stem cells, while NHEJ is more common in already differentiated cells.
- Location of the DSB: DSBs in transcriptionally active regions are mainly repaired by HDR. Also, HDR efficiency varies between regions.[18]

Scientists have also modified the CRISPR-Cas9 system in a way that increases the likelihood of HDR: using two guide RNAs instead of one. Each guide RNA will create a double-stranded break, and having two double-stranded breaks increases the chances for the cell to use HDR over NHEJ.

Questions

- In this class, we will be converting Gal4 driver lines to LexA driver lines. LexA and Gal4 are both located in p-elements on the second chromosome. Do you think that HDR or NHEJ will better serve our purpose? Why?

NHEJ vs. HDR

What are the advantages of NHEJ in comparison to HDR?

1. Unlike homology-directed repair, there is no requirement for a repair template, that being a sister chromatid or homolog for the DNA polymerase to build off of during repair.
2. There is not a large amount of new DNA being synthesized as in homology-directed repair, where completely new nucleotides are being added. Due to this “fast-tracked” method, the repair usually occurs in tens of minutes and is the main method within mammalian cells for repairing the double-stranded breaks caused by the CRISPR/Cas9 system as well as random “endogenous or exogenous stresses” that can occur.

What are the disadvantages of NHEJ in comparison to HDR? Why can’t we use NHEJ for gene knock-in and why is HDR necessary?

1. NHEJ is not a fine-tuned process. There is a high possibility of mutations occuring due to the error-prone nature of the enzymes involved in the cleaning, modification, and ligation of the two previously incompatible strands. Therefore, HDR is often used in experimental designs such as Drosophila gene replacement to provide for more accurate results.
2. NHEJ also involves significant nuclease activity in which the broken ends of the DNA are further destroyed before ligation occurs. Therefore, the repaired DNA is not a copy of the original DNA. Significant portions are missing and, subsequently, the repaired DNA does not code for the same proteins and does not have the same functionality as previously.
3. Homology-directed repair is necessary for gene knock-in as it uses an introduced homologous sequence to copy new genetic information into the section of the genome where the double-stranded break occurred. Therefore, new genetic information is acquired simultaneously as the broken ends are repaired.

[18] https://www.dropbox.com/s/4vzh1HDRv2l18prg/Lin%20%26%20Potter%202016.pdf?dl=0

CRISPR Glossary

The terminology behind CRISPR contains many abbreviations and acronyms to help geneticists save time and space. Use this section as a reference as you read through.

- CRISPR: Clustered Regularly Interspaced Palindromic Repeats, sometimes abbreviated with "cr"
- CRISPR Array: The section of the genome that contains the series of palindromic repeats (CRISPRS) and the alien DNA between them.
- Cas: CRISPR associated
- Cas protein: CRISPR associated proteins, molecular "scissors"
  - Cas9: the typical molecular "scissors", uses a gRNA to create a double-stranded break in the DNA strand at the target site.
  - Cas1 and Cas4: other forms of the cas protein, used in acquisition of alien DNA
  - Cas3, and Cas10: other molecular "scissors" like Cas9, are less commonly used.
- DSBs: Double-stranded Breaks, clean break of both DNA strands
- PAM sequences: Protospacer adjacent motifs, the recognition sequence on DNA for CRISPR
- HDR: homology-directed repair, precise DNA repair method used to repair double stranded breaks
- NHEJ: nonhomologous end joining, imprecise DNA repair method
- *E. coli* (*Escherichia coli*): widely studied bacteria that in which CRISPR was found in 1987
- HACK procedure: homology assisted CRISPR knock-in, basically our whole procedure and goal (using CRISPR to make a gene dysfunctional and copy our functional desired allele into the location)
- 3xP3 promoter: A promoter for genes that are expressed in the eye cells of *Drosophila*. A gene with this promoter will only be expressed in the eye cells of *Drosophila*.
- Binary expression system: A useful tool geneticists use to manipulate transgene expression in tissue-specific patterns.
- NLS(unk): An "unknown" nuclear localization sign; in this procedure, a peptide sequence appended to the Cas9 gene on the X-chromosome in HACK females. It directs the translated Cas9 protein from ribosomes back into the nucleus.
- vas(a): A protein that is expressed during the early embryonic development of *Drosophila*. It is critical in the formation of the primordial germ cells, which give rise to the germ cells.
- U6: U6 is a promoter found in Drosophila involved in spliceosome formation.

RNA Types

- crRNA: CRISPR RNA, the RNA which contains the target cutting site
- gRNA: guide RNA, a catch-all term for the RNA that binds with the Cas-protein.
- sgRNA: single-guide RNA, a combination tracrRNA and crRNA hybrid used for CRISPR/Cas9.

- tracrRNA: Trans-activating CRISPR RNA, a naturally occurring, small RNA that binds crRNA to the cas9 protein. The sequence is the complement to the palindromic repeats of the CRISPR array

Use of CRISPR/HDR to Convert Gal4 Lines to LexA Lines and a Bit About the Gene Expression After the Conversion

Let's look at this diagram in a bit more detail…

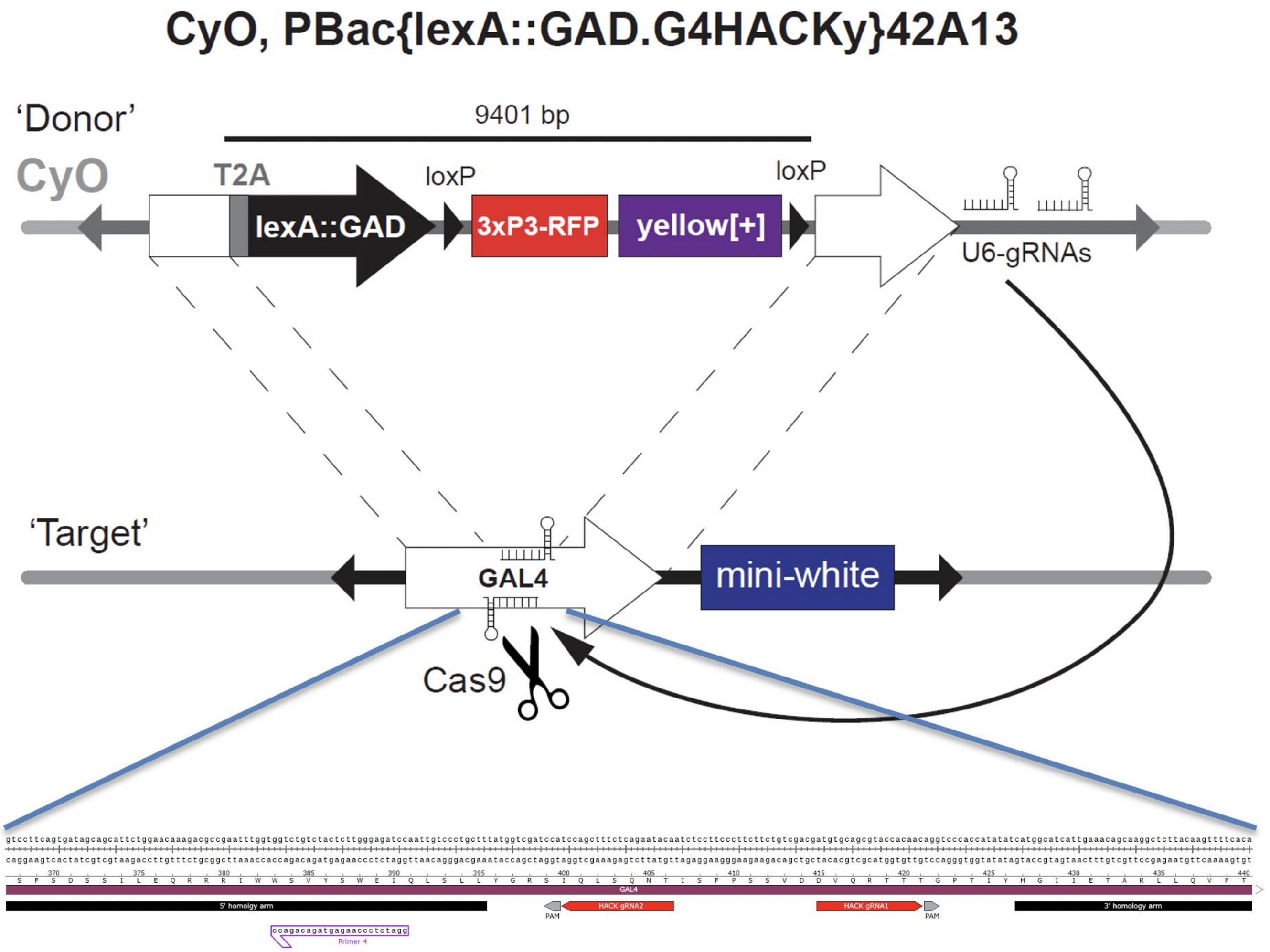

The middle of the Gal4 sequence will be recognized by two guide RNAs (red in the bottom portion of the picture). The guide RNAs will recruit Cas9 to make two double strand breaks. We are hoping these two double strand breaks will induce the broken DNA to undergo HDR rather than NHEJ repair process.

When the broken DNA finds its homology sequences in the donor transgene, then the broken DNA will be repaired using the donor sequence as a template. The resulting sequence (the converted LexA) will look like this.

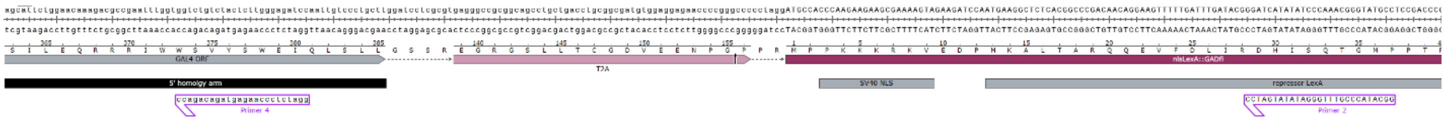

An important thing to remember is that the repair template does NOT contain the sequence which was recognized by the original guide RNA.

Why is this important?

After the conversion…
GAL4-hsp70_3'UTR is changed to GAL4(truncated)-T2A-nlsLexA::GAD-hsp70_3'UTR.

Below are explanations of some of the important things to notice about this construct and how it will behave in a eukaryotic cell.

- The 3'UTR from hsp70 gene is to ensure the RNA polymerase II will fall off after making mRNA from the transgene.

- Nearby enhancers and TATA box (https://en.wikipedia.org/wiki/TATA_box) will recruit transcriptional activators and TATA-binding protein (TBP), forming a protein complex to bring RNA polymerase II to TATA box, and the transcription starts around 30 bp after TATA box by RNA polymerase II.

- The transcribed mRNAs will be recognized by ribosomes because the 5' end of mRNA was further modified by adding extra methylated GTPs. Ribosomes start to 'read' the sequence when they see the first 'AUG' - the start codon.

- Ribosomes will translate the subsequent 'codons' until they see a '2A cleavage sequence'. The ribosome does not make the peptide bond between Glycine and Proline peptides in the amino acid sequence EGRGSLLTCGDVEENPGP (these letters are the single letter abbreviations for the amino acids). In the picture below the top image shows a fusion protein comprised of the products of two genes while the bottom image shows the effect of inserting the 2A ribosome skipping sequence between the two genes.

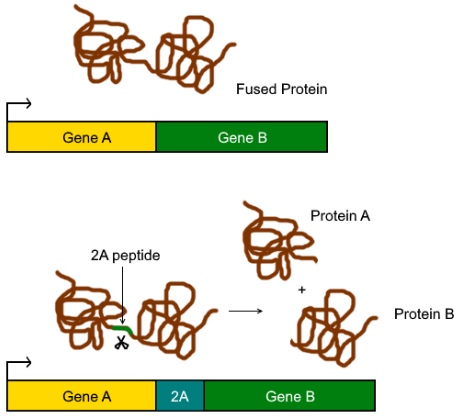

The ribosome skipping results in a truncated GAL4 protein which should have no function (theoretically…this is an icky part…see below) and a separate protein called nlsLexA::GAD. (https://en.wikipedia.org/wiki/2A_self-cleaving_peptides)

- nlsLexA::GAD is a fusion protein of
  - nuclear localization protein (nls)
  - lexA binding protein
  - transcription activator protein GAD

  This nlsLexA::GAD protein will bind to the LexAop2-transgene if there is one in the genome. Taken together, the nearby enhancers of the original GAL4 line will now drive the production of nlsLexA::GAD protein in the same cells instead of GAL4 protein.

Two questions about this fusion protein…

1. Why do you think it requires nls?

2. Why do you think it requires GAD - particularly since we never mentioned GAD as part of the Gal4 gene?

Now the icky part…if the T2A efficiency for the ribosome skip is low, then you will get mostly conjoined, weird proteins. The truncated GAL4 protein (unfortunately) has a part of the sequence required for UAS binding. If the non-cleaved truncated GAL4-nlsLexA::GAD protein is made, it may still be able to bind to UAS DNA sequence and recruit RNA polymerase to TATA box via GAD, resulting in a possibly faint expression of UAS-mCD8::EGFP.

Which Gal4 lines are we going to HACK and how to research a line?

Each of you will fill out this table over several nights of homework to collect information on the lines we are going to convert in this class.

Here is an example of how to research a line…

For the line ID 2017, the following data was shown in BDSC (Bloomington Drosophila Stock Center).

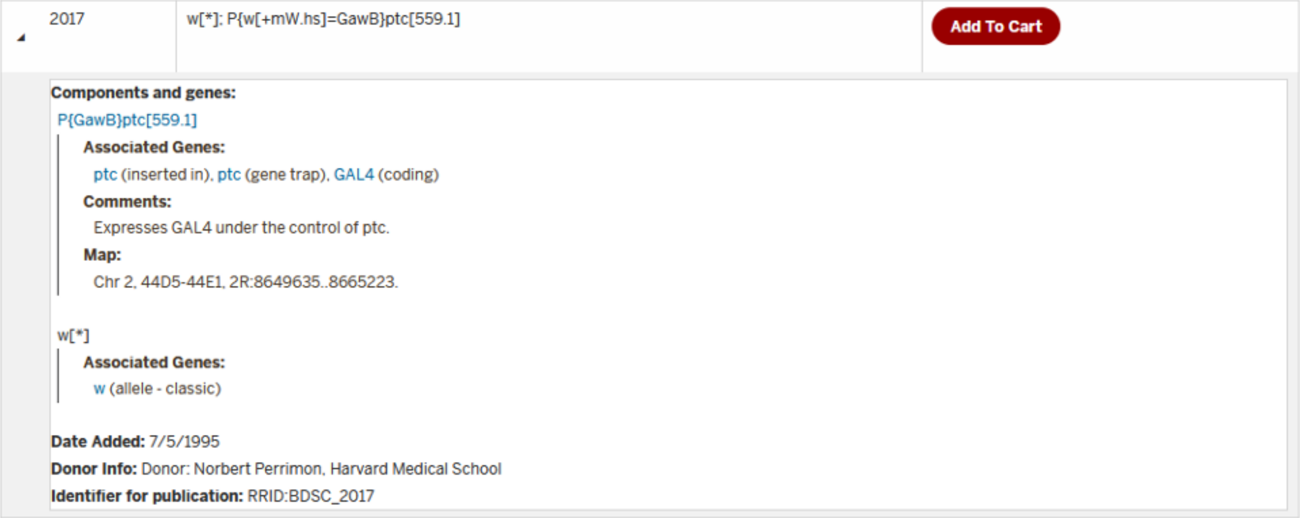
2017 w[*]; P{w[+mW.hs]=GawB}ptc[559.1] Add To Cart

Components and genes:

P{GawB}ptc[559.1]

Associated Genes:

ptc (inserted in), ptc (gene trap), GAL4 (coding)

Comments:

Expresses GAL4 under the control of ptc.

Map:

Chr 2, 44D5-44E1, 2R:8649635..8665223.

w[*]

Associated Genes:

w (allele - classic)

Date Added: 7/5/1995

Donor Info: Donor: Norbert Perrimon, Harvard Medical School

Identifier for publication: RRID:BDSC_2017

The genotype for line ID 2017 is: w[*]; P{w[+mW.hs]=GawB}ptc[559.1].

Hmm…which tissue is expressing the yeast GAL4 gene in these flies? To find the answer, follow the top link (‘P{GawB}ptc[559.1]), and you will get this page…

FlyBase

FB2023_02, released April 18, 2023

Insertion: Dmel\P{GawB}ptc$^{559.1}$

CanFly 2023

Home | Tools | Downloads | Links | Community | Species | About | Help | Archives | Search | Search FlyBase | Go

General Information

**Symbol**	Dmel\P{GawB}ptc$^{559.1}$	**Species**	*D. melanogaster*
**Name**		**FlyBase ID**	FBti0002124
**Feature type**	transposable_element_insertion_site		
**Also Known As**	ptc-GAL4, ptcGal4, patched-gal4, ptc.Gal4, ptc$^{559.1}$-Gal4, ptc^{Gal4}, ptc::Gal4, P{GawB}559.1, 559.1, GAL4-ptc		

Description

**Inserted Element**	P{GawB}	**Uses**	GAL4 reporter/driver
**Affected gene(s)**	ptc, Scer\GAL4	**Viability / fertility**	fertile, viable
**Associated allele(s)**	ptc$^{559.1}$, Scer\GAL4$^{ptc\text{-}559.1}$	**Stock availability**	4 publicly available
**LINE ID**			

Genomic Location

**Chromosomal Location**		**Sequence Location**	
**Target / Docking site**			

Member of Large Scale Dataset(s)

**Dataset**	
**Description**	

Detailed Mapping Data

**Chromosome (arm)**	
**Sequence Location**	
**Orientation**	
**Cytological location (computed by FlyBase)**	
**Cytological location (reported)**	
**Insertion into Natural transposon**	
**Comments concerning location**	

Sequence Data

**Flanking sequence**	

Inserted Element

**Construct**	P{GawB}

Report Sections

Open | Close

- General Information
- Detailed Mapping Data
- Sequence Data
- Inserted Element
- Affected Gene(s)
- Alleles and Phenotypes
- Expression Data
- Data on Genetic Line
- Progenitor(s)
- Related Aberration or Balancer
- Stocks
- Crossreferences
- Synonyms and Secondary IDs
- References

Then go to 'Expression Data'

Expression Data

Reporter Expression

No Assay Recorded

Stage	Tissue/Position (including subcellular localization)	Reference
third instar larval stage	wing disc | restricted	(*Feger et al., 1995*)

distribution deduced from reporter

Stage	Tissue/Position (including subcellular localization)	Reference
third instar larval stage	wing disc | restricted	(*Hinz et al., 1994*)
oogenesis	escort cell	(*Rojas-Ríos et al., 2012*)

distribution deduced from reporter (Gal4 UAS)

Stage	Tissue/Position (including subcellular localization)	Reference
embryonic stage	leading edge mixer cell | subset	(*Gettings et al., 2010*)
embryonic stage 10 -- 17	embryonic head | restricted	(*Marquez et al., 2001*)
	organism | segmentally repeated	(*Marquez et al., 2001*)
embryonic stage 13	visceral mesoderm of foregut	(*Page, 2002*)
	embryonic brain | segmentally repeated	(*Page, 2002*)
third instar larval stage	wing disc | restricted	(*Sun and Artavanis-Tsakonas, 1997*)
		(*Marquez et al., 2001*)
	anterior-posterior compartment boundary of the wing disc	(*Usha and Shashidhara, 2010, Speicher et al., 1994*)
	embryonic/larval wrapping glial cell of the eye disc	(*Murakami et al., 2007*)
	epithelium of eye disc | restricted	(*Murakami et al., 2007*)
	larval hindgut imaginal ring | restricted	(*González-Morales et al., 2015*)
wandering third instar larval stage	anterior-posterior compartment boundary of the wing disc	(*Apidianakis et al., 1999*)
adult stage	adult ventral nerve cord | posterior	(*Monastirioti, 2003*)
	adult mushroom body	(*Monastirioti, 2003*)
adult stage | male	cyst cell of testis	(*Wang et al., 2011*)

immunolocalization

Stage	Tissue/Position (including subcellular localization)	Reference
adult stage	adult ventral nerve cord | restricted	(*Saleem et al., 2012*)

Additional Information

Statement	Reference
Drives embryonic expresion in a segment polarity expression pattern, and larval expression anterior to the A/P compartment boundary of the wing disc.	(*FlyBase Curators, 2017*)
Scer\GAL4$^{ptc\text{-}559.1}$ drives expression at the boundary of the anterior, or 'H1', and posterior, or 'H2', regions of the hindgut imaginal ring.	(*González-Morales et al., 2015*)
Scer\GAL4$^{ptc\text{-}559.1}$ drives expression in octopaminergic neurons in the abdominal ganglion.	(*Saleem et al., 2012*)
Scer\GAL4$^{ptc\text{-}559.1}$ drives expression in anterior cells immediately adjacent to the A/P compartment bounary of the wing disc.	(*Usha and Shashidhara, 2010*)
Adult expression is observed in the mushroom body and in the posterior tip of the thoracic ganglion.	(*Monastirioti, 2003*)

Report Sections

Open | Close

- General Information
- Detailed Mapping Data
- Sequence Data
- Inserted Element
- Affected Gene(s)
- Alleles and Phenotypes
- Expression Data
- Data on Genetic Line
- Progenitor(s)
- Related Aberration or Balancer
- Stocks
- Crossreferences
- Synonyms and Secondary IDs
- References

Since you are going to dissect larvae, see ‘third instar larval stage’ under ‘distribution deduced from reporter’. Hmm…’anterior-posterior compartment boundary of the wing disc’, so I need to dissect wing discs not brains for this GAL4 line. How should the wing discs look? The earliest report of this line was from ‘Speicher et al, 1994’. Let’s follow this reference…

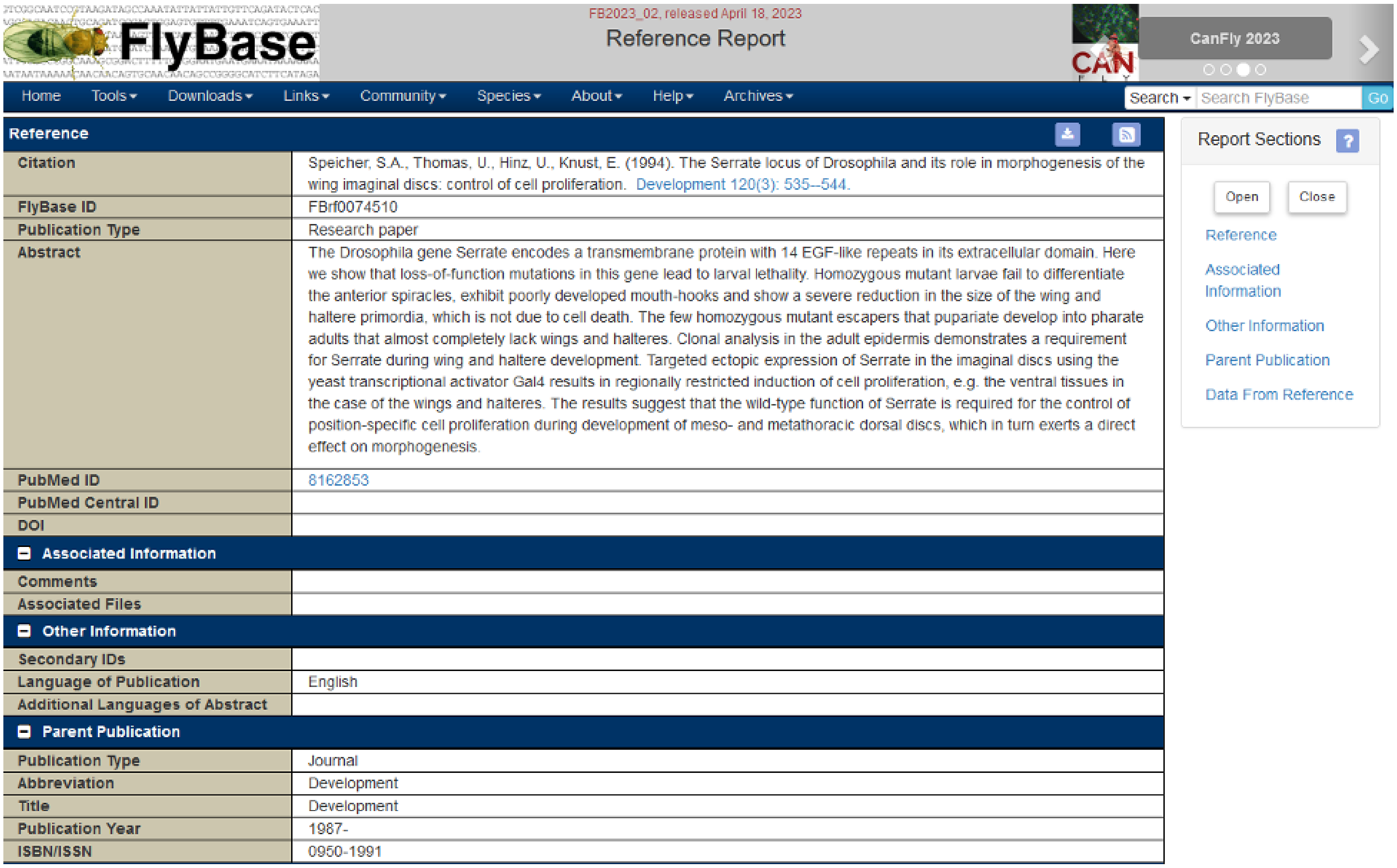
FlyBase

FB2023_02, released April 18, 2023

Reference Report

CanFly 2023

Home | Tools | Downloads | Links | Community | Species | About | Help | Archives | Search | Search FlyBase | Go

Reference

Citation	Speicher, S.A., Thomas, U., Hinz, U., Knust, E. (1994). The Serrate locus of Drosophila and its role in morphogenesis of the wing imaginal discs: control of cell proliferation. Development 120(3): 535--544.
FlyBase ID	FBrf0074510
Publication Type	Research paper
Abstract	The Drosophila gene Serrate encodes a transmembrane protein with 14 EGF-like repeats in its extracellular domain. Here we show that loss-of-function mutations in this gene lead to larval lethality. Homozygous mutant larvae fail to differentiate the anterior spiracles, exhibit poorly developed mouth-hooks and show a severe reduction in the size of the wing and haltere primordia, which is not due to cell death. The few homozygous mutant escapers that pupariate develop into pharate adults that almost completely lack wings and halteres. Clonal analysis in the adult epidermis demonstrates a requirement for Serrate during wing and haltere development. Targeted ectopic expression of Serrate in the imaginal discs using the yeast transcriptional activator Gal4 results in regionally restricted induction of cell proliferation, e.g. the ventral tissues in the case of the wings and halteres. The results suggest that the wild-type function of Serrate is required for the control of position-specific cell proliferation during development of meso- and metathoracic dorsal discs, which in turn exerts a direct effect on morphogenesis.
PubMed ID	8162853
PubMed Central ID	
DOI	

Associated Information

Comments	
Associated Files	

Other Information

Secondary IDs	
Language of Publication	English
Additional Languages of Abstract	

Parent Publication

Publication Type	Journal
Abbreviation	Development
Title	Development
Publication Year	1987-
ISBN/ISSN	0950-1991

Report Sections

Open | Close

Reference

Associated Information

Other Information

Parent Publication

Data From Reference

Unfortunately, the paper is behind a paywall. If this is the case, just send me (Sangbin Park) PubMed ID (8162853), then I’ll send you a copy of the paper which shows…”(**E**) Fate map of the wing imaginal disc according to Bryant (1975). (**F**) The Gal4$^{559.1}$ activator line drives *lacZ* expression (this is the role that GFP plays for us in our crossing program) in a narrow stripe along the anterior-posterior compartment boundary in the entire disc.”

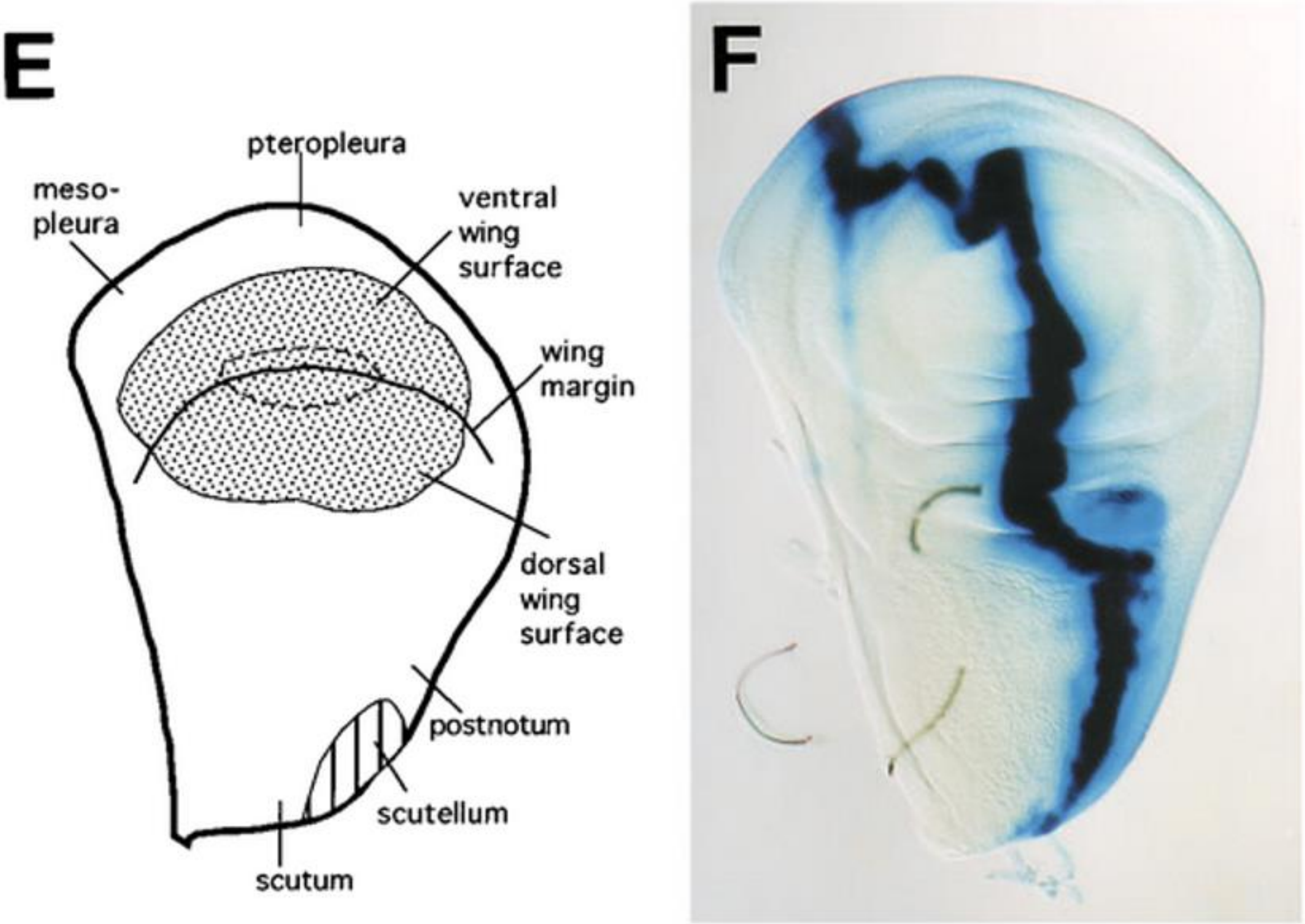

Alternatively, you can also find your GAL4 expression data in here as well - Frequently Used GAL4 Drivers- and find your allele name ‘559.1’. You may need to scroll to the right to see “Description: Drives embryonic expression in a segment polarity expression pattern, **and larval expression anterior to the A/P compartment boundary of the wing disc**”

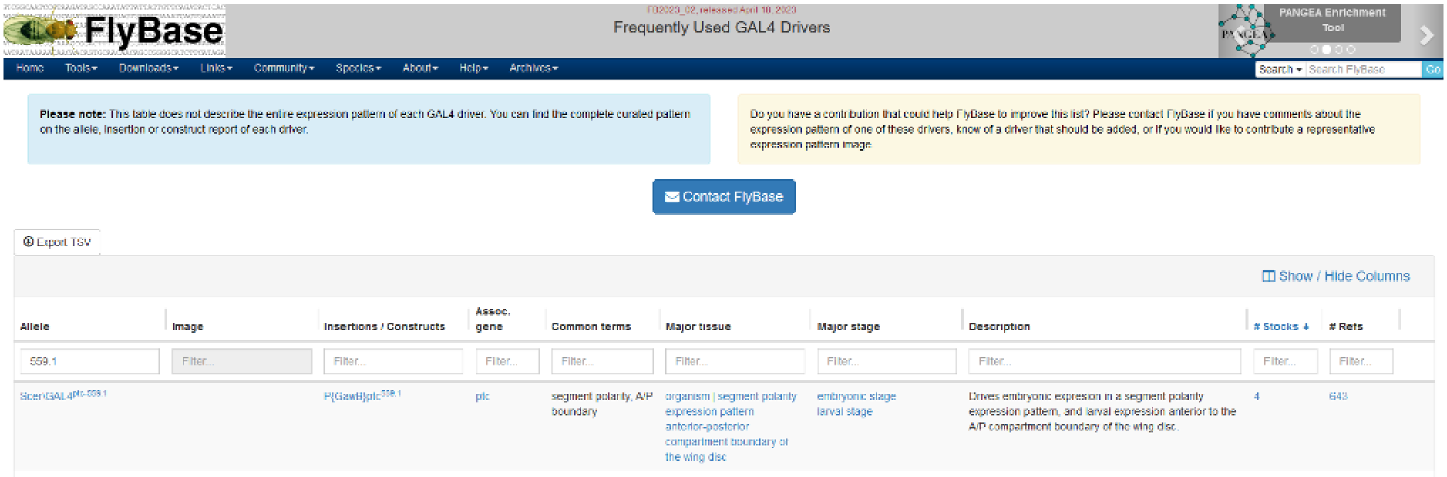
FlyBase
FB2023_02, released April 10, 2023
Frequently Used GAL4 Drivers
PANGEA Enrichment Tool
Home Tools Downloads Links Community Species About Help Archives
Search Search FlyBase Go

Please note: This table does not describe the entire expression pattern of each GAL4 driver. You can find the complete curated pattern on the allele, Insertion or construct report of each driver.

Do you have a contribution that could help FlyBase to improve this list? Please contact FlyBase if you have comments about the expression pattern of one of these drivers, know of a driver that should be added, or if you would like to contribute a representative expression pattern image.

Contact FlyBase

Export TSV

Show / Hide Columns

Allele	Image	Insertions / Constructs	Assoc. gene	Common terms	Major tissue	Major stage	Description	# Stocks	# Refs
559.1	Filter...	Filter...	Filter...	Filter...	Filter...	Filter...	Filter...	Filter...	Filter...
Scer\GAL4$^{plc-559.1}$		P{GawB}plc$^{559.1}$	plc	segment polarity, A/P boundary	organism | segment polarity expression pattern anterior-posterior compartment boundary of the wing disc	embryonic stage larval stage	Drives embryonic expresion in a segment polarity expression pattern, and larval expression anterior to the A/P compartment boundary of the wing disc.	4	643

Find out which tissues your GAL4 line expresses in, and check images in prior publications.

While you are on this web page, check out how many references have cited your GAL4 line.

If you follow the #Refs link, you will see the list of papers that used this GAL4 line. When was the last time your GAL4 was cited? One of the latest papers was published in the journal ‘Science’ by de Vreede et al (2022). Let’s see how they used this GAL4 line in their study. Most of the recently published papers are now available on the NIH website. For de Vreede et al (2022), find the paper at [Epithelial monitoring via ligand-receptor segregation ensures malignant cell elimination - PMC]
and the Figures 1G and H show ‘ptc^{ts}>’ to express shRNA of dlg genes (dlg knockdown).

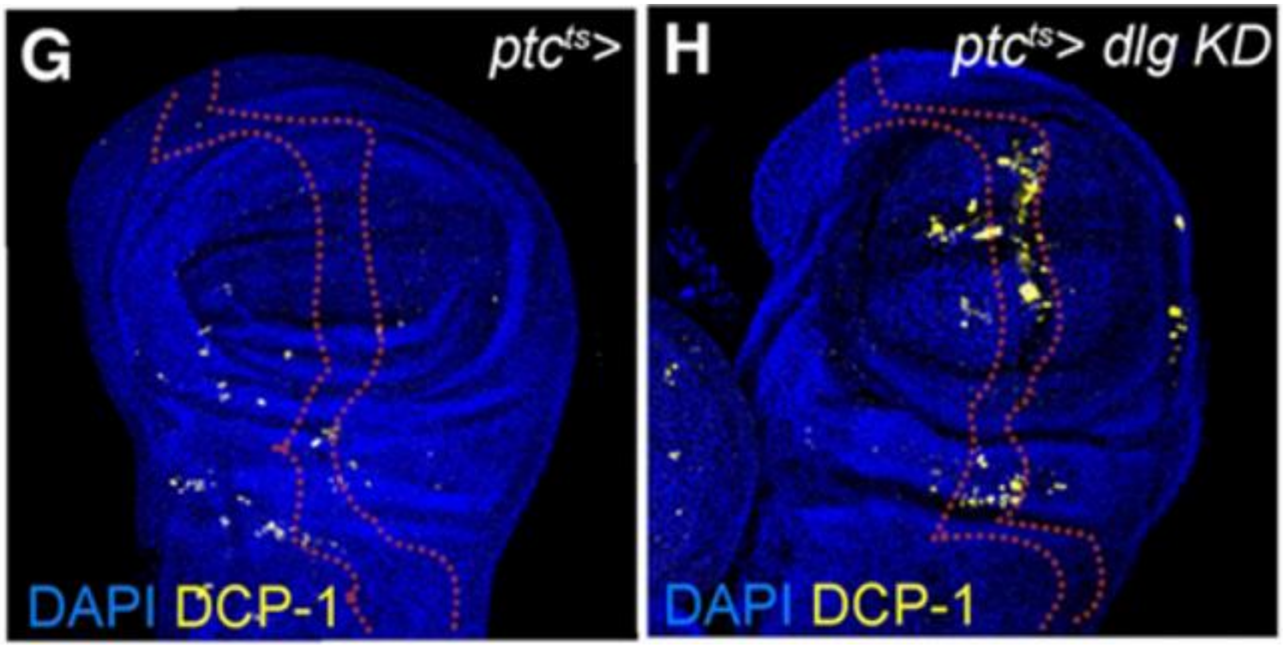

So, since the first characterization of P{GawB}ptc[559.1] in 1994, people still use the same line again and again. Why? One of the reasons is the extensive expression characterization history of this GAL4 line. Because of the prior history, scientists feel comfortable and safe using this line over other less characterized GAL4 lines which might raise some questions from peers. Also, since you are using the same GAL4 lines as the previous studies, you can compare your results to historic data as well. Therefore, it is very important to document the “identical” expression patterns of the original GAL4 and converted LexA lines. Once you document them, scientists WILL use your converted LexA lines to express LexAop-fused transgene in the same population of the cells as the original GAL4 expressed in.

To learn more about the gene which was trapped, click on the gene name (ptc in this case) from this window in FlyBase.

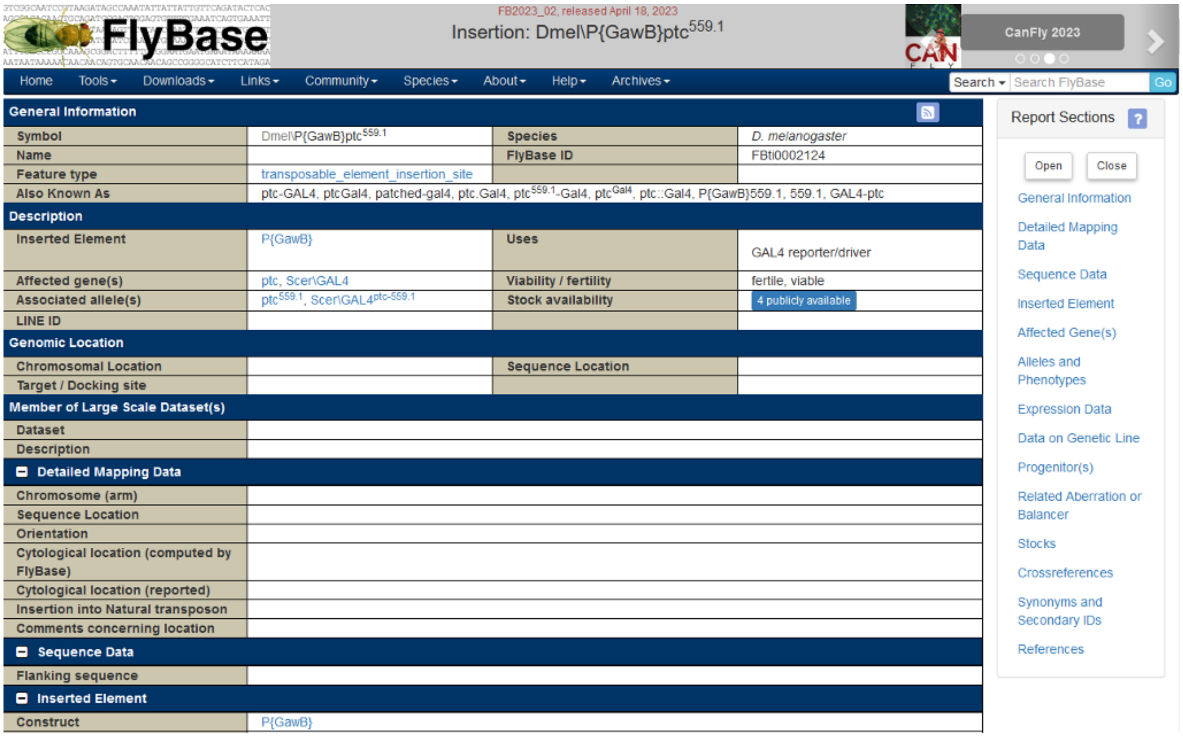

This will bring you to this window…

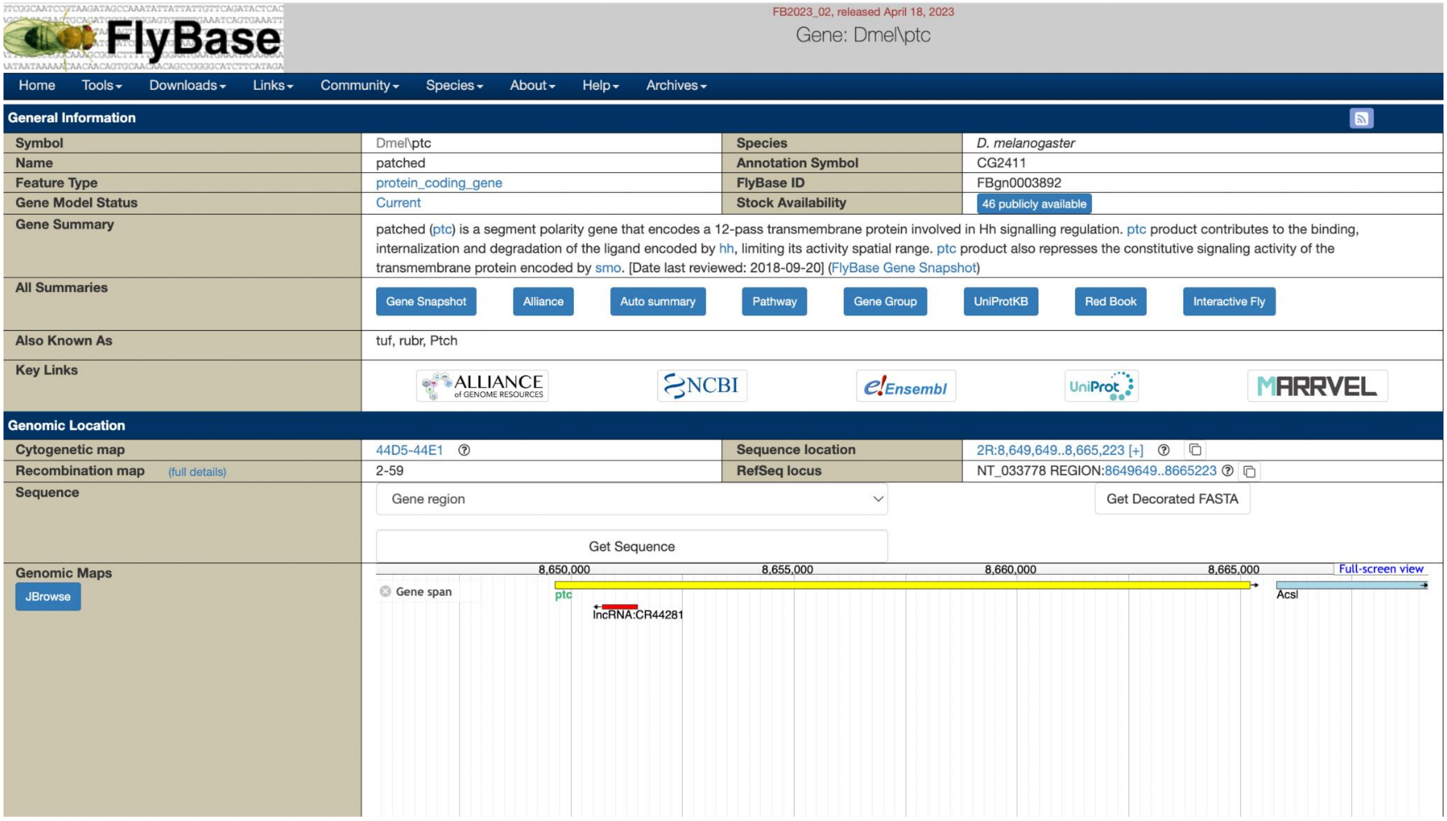

Click on the JBrowse button on the left and that will bring you to this window…

This window contains a lot of information! For example…you can see the number of exons and introns in the gene (6 and 5 in this case). You can determine whether there are alternative splice versions of this gene (no in this case - but take a peek at the gene to the right and you can see there are 4 alternative splice forms shown) . You can determine which strand of DNA is read by RNA polymerase.

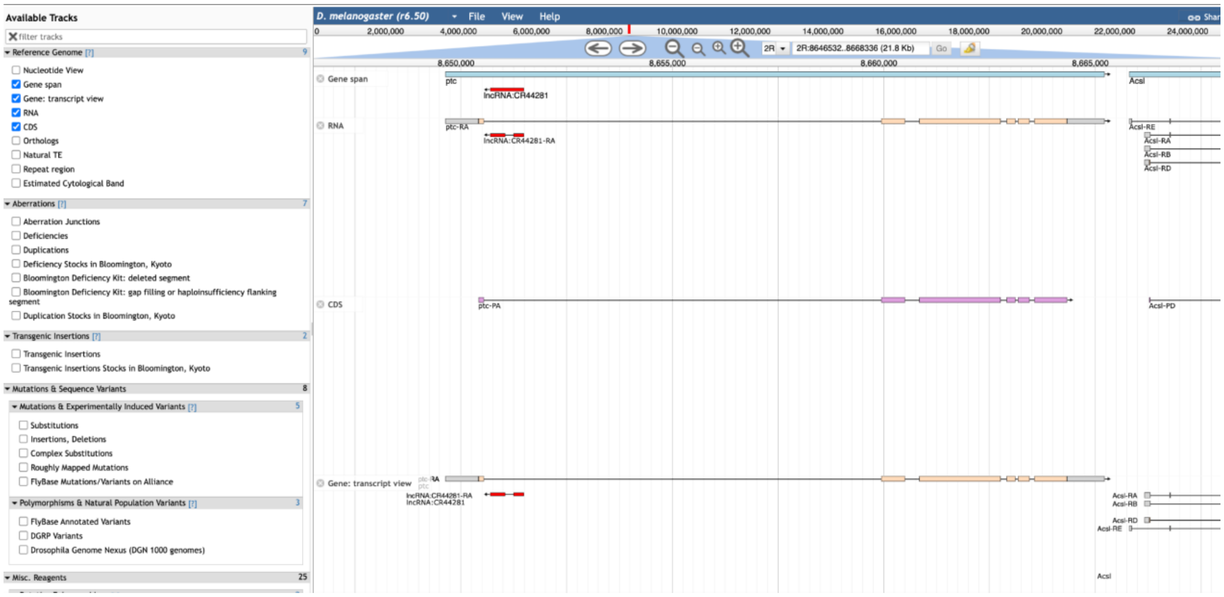

Click the ortholog (pause to look up the word ortholog if you do not know what it means…) option on the left menu and then click the yellow bar representing the ortholog. Up will pop a little window which allows you to see the orthologs of this gene in different species, including humans.

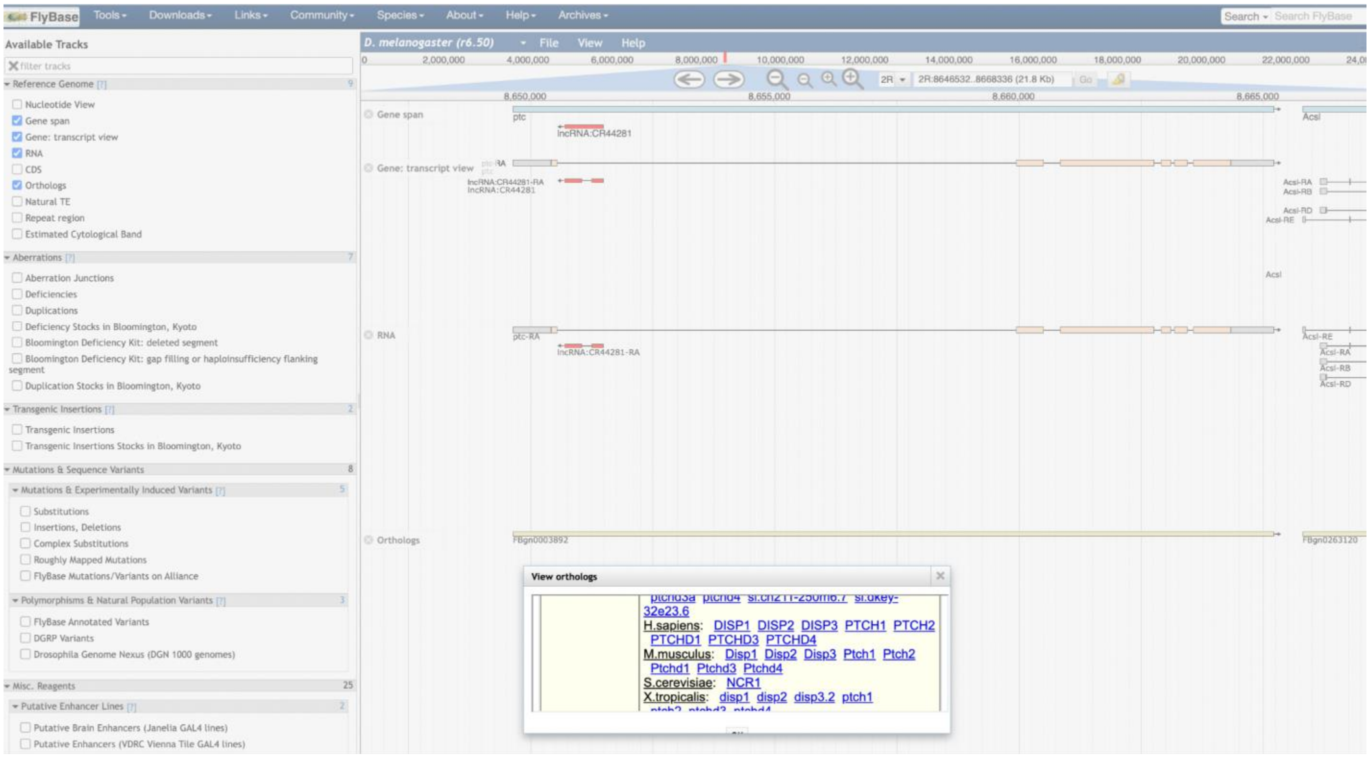

We are going to attempt to convert eleven lines total. There are more than eleven stocks listed on this table.

Which of these do you think we did NOT include in this class and why?

Each student will convert two lines. There are twelve students total and so we will attempt to convert each line two or three times.

We have randomly assigned each stock a new, temporary number for this term. When we look at images of our converted lines we will try to determine which temporary number corresponds to each stock. We are doing this (assigning random numbers rather than telling you what stocks you are converting) to avoid confirmation bias.

What is confirmation bias?

Crossing Program

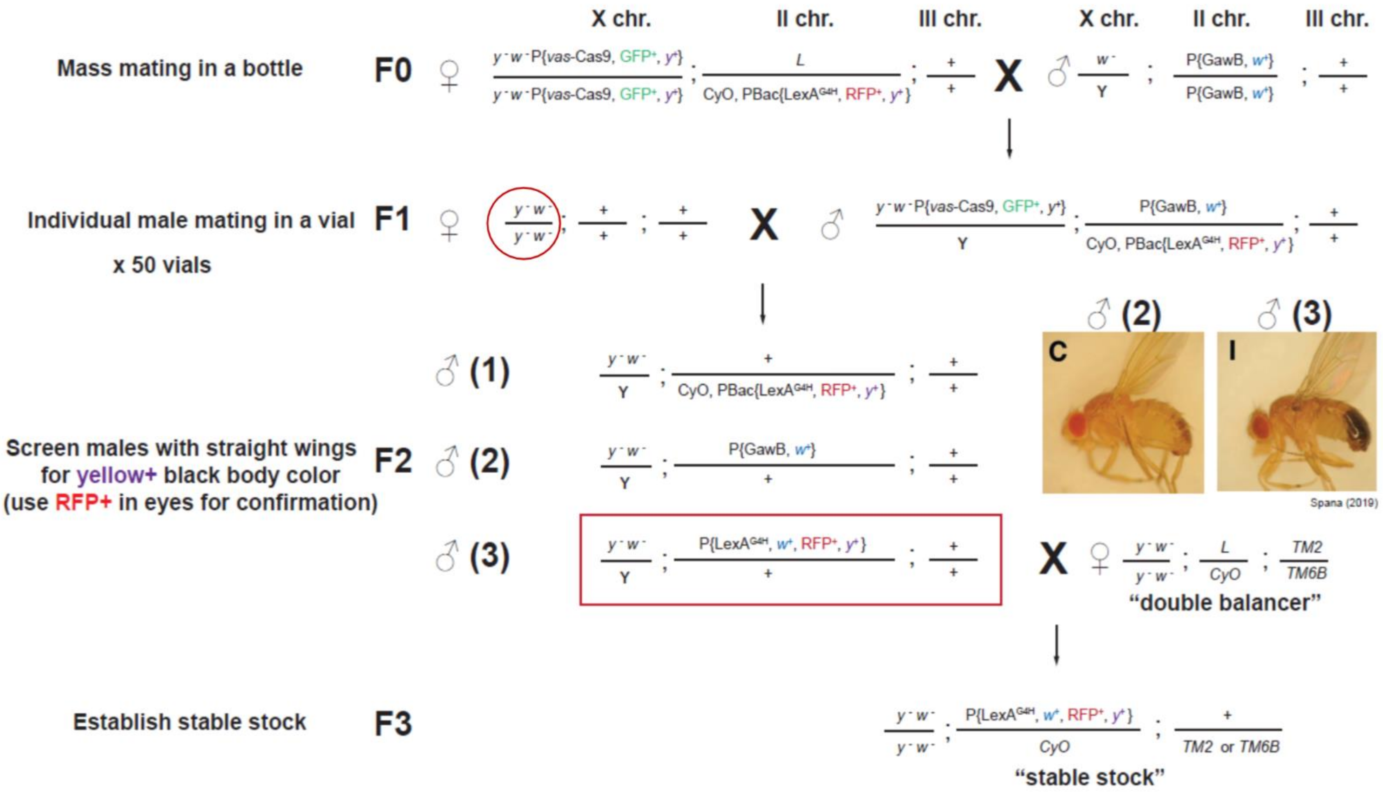

Generation	Bottle or vial	# males needed per bottle or vial	# virgin females needed per bottle or vial	# of bottle/vials needed per GAL4 lines per student	Flip or no?	Goal...
F_0<br><br>Hack female X Gal4 donor line male	bottle	5-10	50	1	Yes! (once)	Produce at least 50 "target males" for F_1
F_1<br><br>Yellow white female X target male	vial	1	3	40	no!	Produce as many males as possible to screen for "converted males"
F_2<br><br>Converted male X double balancer female	vial	1	4	Collect 2 independent (collected from different fathers) converted males per Gal4 line	YES! (twice)	Produce flies to create "stable stock"

Directions for Sorting the Offspring from the F_1 Cross

CRISPR is going to take place in the male parent of the F_1 cross.

We want to tally offspring from this cross to calculate the conversation rate - the rate at which CRISPR is taking place in these males. This is interesting because it can be impacted by the size of the DNA we are trying to insert using HACK (homology assisted CRISPR knock-in) and the relative locations in the genome of the region of homology and Gal4, the target of CRISPR.

We will calculate another value from our data and then figure out what this ratio reveals about the process of CRISPR.

Here are the steps you will take for **every vial**, **every time** you sort offspring…

1. Separate males from females
2. Discard females
3. Separate curly (white eyed) from non-curly (red eyed) males
4. Count the curly (white eyed) males, record this number and then discard these flies
5. Count the non-curly (red eyed) males and record this number
6. Search for honey males among the non-curly (red eyed) males
7. Discard all yellow males
8. These non-curly (red eyed), honey males are the CONVERTED males. Double check by confirming that they also express RFP and then record the number of these flies.

Here is the link to the data table where you should record this information.

A few additional directions…

- Continue checking until you have 5 converted males (from at least 2 different vials) per line.
- Use 2 of the males to establish the F_2 cross. Select males from 2 different vials to ensure that each was generated from a unique CRISPR/HDR event.

Problems to think about after collecting data from the F_1 cross…

1. We calculated the ratio of curly white males to non-curly red males (both yellow and honey). Considering meiosis only - what would you expect this ratio to be and why?

2. What would it suggest if the ratio of curly white males to non-curly red males (both yellow and honey) was consistently greater than one for all (or the vast majority of) lines?

3. What would it suggest if the ratio of curly white males to non-curly red males (both yellow and honey) was consistently greater than one for all (or the vast majority of) lines BUT statistically significantly variable between lines (and consistent among replicates of the same line)?

4. What would it suggest if the ratio of curly white males to non-curly red males (both yellow and honey) was consistently greater than one for all (or the vast majority of) lines AND there was no statistically significant variability between lines?

5. Hm…lots of interesting ideas about this ratio…before we can think more deeply about it we should probably do some controlled crosses to better understand what is happening. What controls would you suggest and what would each allow you to demonstrate?

Problems to Understand the Crossing Program

Morgan Problem

Thomas Hunt Morgan was the first researcher to use fruit flies for genetics research. Ever. In April 1914 he published a paper to share some unusual results he uncovered when experimenting with two traits – body color and wing length. In flies, gray body is dominant to black and long wings are dominant to vestigial. Morgan first crossed two flies heterozygous for both traits and found the following data:

Gray Long.	Black Long.	Gray Vestigial.	Black Vestigial.
4569	2151	1626	0

Intrigued he went on to cross true-breeding black, long winged flies with true breeding gray, vestigial winged flies to yield F_1 flies which were all gray with long wings. He crossed his male F_1 flies with black, vestigial females and recorded the following phenotypes in his F_2.

Gray Long.	Black Long.	Gray Vestigial.	Black Vestigial.
0	19	19	0
0	30	33	0
0	34	20	0
0	193	115	0
0	174	118	0
0	542	416	0
0	992	721	0

He then did the reciprocal cross: female F_1 flies with black, vestigial males and recorded the following results:

Gray Long.	Black Long.	Gray Vestigial	Black Vestigial.
66	283	201	58
65	243	224	50
55	218	169	42
20	113	112	29
71	311	210	47
17	153	181	20
44	231	218	48
338	1,552	1,315	294

Based on this experimental design, what was the question Morgan addressed?

What conclusion did this data set allow Morgan to draw?

Do you find his result surprising or not? Why?

(all data in this problem taken from original paper by Morgan publish April 1914 by Woods Hole Marine Biological Laboratory)

F_0 Cross Problems

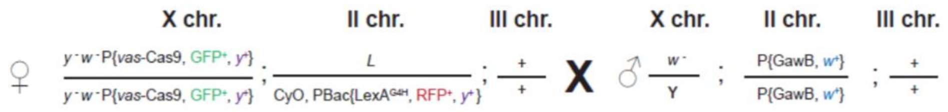

1. What is the purpose of this cross?

2. Describe the appearance of the female flies under natural lighting. Think about body color, eye shape and color and wing appearance.

3. Here is another little detail… a 3xP3 promoter was used in front of both the GFP+ and RFP+ genes. Look this up and then take a guess about how these flies might appear under different lighting conditions.

4. Describe the appearance of the male donor line flies. Think about body color, eye shape and color and wing appearance.

5. The donor line male came from a vial of donor line stable stock. In some cases the Gal4 P element cannot be homozygous. Why might this be true?

6. What homologous chromosome would be best to use as the homolog to the chromosome carrying the P element in this situation - L, CyO or wild type?

7. Determine all the genotypes and phenotypes for the offspring produced by this cross and the percent you expect of each.

F_1 Cross Problems

$$\text{♀}\ \frac{y^- w^-}{y^- w^-};\ \frac{+}{+};\ \frac{+}{+}\quad \mathbf{X}\quad \text{♂}\ \frac{y^- w^- P\{vas\text{-}Cas9,\ GFP^+,\ y^+\}}{Y};\ \frac{P\{GawB,\ w^+\}}{CyO,\ PBac\{LexA^{G4H},\ RFP^+,\ y^+\}};\ \frac{+}{+}$$

1. Describe the features of the dream sperm that we are hoping our target male produces. Think about meiosis and CRISPR in answering this question.

2. The creation of the dream sperm involves two distinct processes - CRISPR and MEIOSIS. Assume that CRISPR and HDR has occurred correctly in every cell in the animal's body. Considering JUST meiosis, what fraction of sperm created by this animal will be the dream sperm?

3. Why is the white eye trait from the female line very handy (though not absolutely critical) for the F1 cross?

4. What is RFP+ and why is it handy, though not critical, in this cross?

5. What is the purpose of the y+ allele on chromosome II in the male parent?

6. Dr. Sangbin Park casually mentioned this one day…"I have seen some GAL4 stocks carry undocumented Dp(1;Y)y+ instead of a regular Y chromosome." If this happened in one of the Gal4 stocks we are trying to convert would this be an annoying, but manageable problem or an experimentally fatal problem? Explain. (might need to go back to the F0 to think about the origin of the Y chromosome in the F1 cross)

7. You will notice that the F1 male carries both *Drosophila* P element transposons and something called "PBac". The job being done by "PBac" could have been done just as easily by a *Drosophila* P element. It is worth pausing for just a moment to understand what "PBac" is and where it came from. Here is the website from the person who identified and named the PBac element - what is this thing, where did it come from and how is it used?

8. Look again at the cas9 on chromosome I in the F1 male. The vas in front of cas9 notes the promoter being used to control the expression of the cas9 protein. Below are the "expression ribbons" for the vas promoter (taken from flybase). These show where and when the vas promoter is active. As a way to probe your understanding of the importance of this information - give some examples of what the "expression ribbons" would look like in situations where cas9 was successfully expressed in the F1 male fly but there would be NO CHANCE that the fly could create the dream sperm.

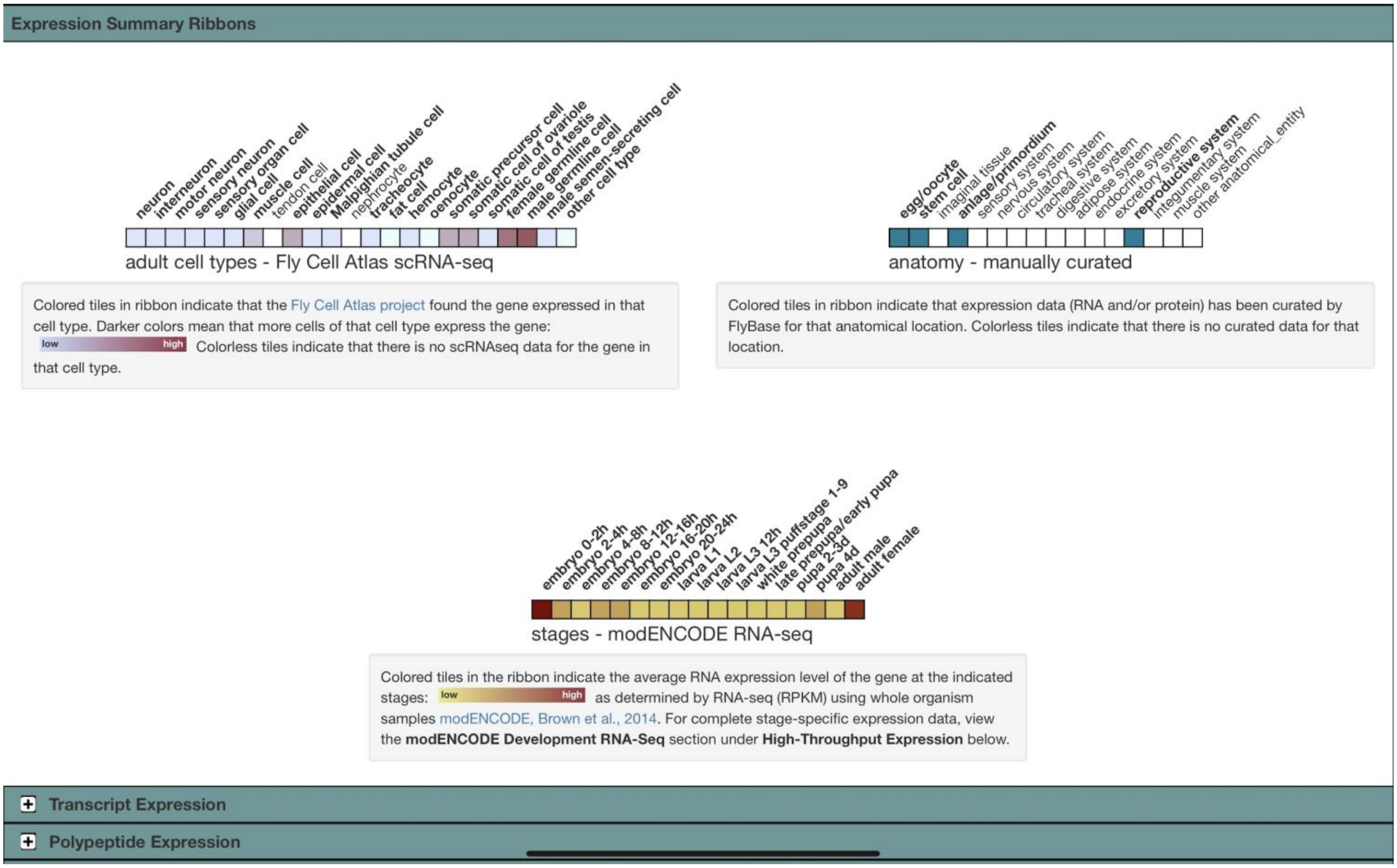

9. In theory CRISPR is pretty efficient. If cas9 and the guide RNA are produced then CRISPR should take place. If this assumption is true and we observe that the dream sperm is actually produced quite infrequently (as seen through a very low conversion rate) - then what does this suggest about when cas9 is actually turned on…very early in development or relatively later?

10. Our vas-cas9 element also has NLS(unk). Look that up in flybase...what is it and why does it make sense that it is a necessary part of the construct?

11. The other element needed for CRISPR besides cas9 is the guide RNA. Would you guess that the sequence to produce the guide RNA is on the p element on the X chromosome (with cas9) or the p element on the second chromosome (with the sequence to be used in HDR in this specific protocol) ? (a hint here…think back to our conversations about why binary systems are advantageous as compared to one p element which traps the expression of a specific enhancer and a different one which has a specific reporter gene…)

12. If you combined all transgenes expressing Cas9, gRNA, and HACK donor all together on the CyO balancer chromosome, would CRISPR and HDR still work? If so, why did we not design it that way? (hint…think about the risk of an accidental cross involving a fly with this genotype in a lab using Gal4 drivers….)

F_2 Cross Problems

$$\frac{y^- w^-}{Y} ; \frac{P\{LexA^{G4H}, w^+, RFP^+, y^+\}}{+} ; \frac{+}{+} \quad \mathbf{X} \quad ♀ \frac{y^- w^-}{y^- w^-} ; \frac{L}{CyO} ; \frac{TM2}{TM6B}$$

“double balancer”

1. What is the purpose of this cross?

2. What was the phenotype of the female parent and which of her chromosomes are balancer chromosomes? (look at the balancer section of the manual for help with this question)

3. The decision to use a “converted male” rather than a “converted female” was intentional. There are at least four reasons for this choice. Can you identify (and explain) any of the reasons?

4. What color eyes will the ideal offspring have (under natural light) and why is this critical?

5. The ideal offspring from this cross will carry the CyO chromosome. Why is this important?

6. Draw the decision tree you will use to sort through the offspring created from this cross. Sort flies into three categories:
    - flies to use to create the stable stock
    - flies which cannot be used for the stable stock but can be used for the molecular work
    - flies which should be discarded

The Drosophila Y Chromosome

Origin and Evolution of Y Chromosomes: Drosophila Tales

The Modern View of B Chromosomes

Articles

Duffy Article, glossary and questions

Duffy et al.

Metazoan - multicellular animal with true tissues

GAL4 - yeast transcription factor with 881 AA's that activates galactose-induced genes

UAS - upstream activating sequences - 4 (or more) 17-base pair DNA sequences that act as enhancers (binding sites) for the GAL4 transcription factors

1. Look at Figure 1 in Duffy. What is the "responder" in this diagram?

2. What is the "driver"?

3. The pattern of alternating stripes in the embryo is caused by the **normal** expression of the *even-skipped* gene. How was the GAL4 driver inserted next to the regulatory elements (enhancers) for the *even-skipped* gene?

4. List the advantages of "this bipartite approach".

5. What is "an enhancer-trap GAL4 construct, pGAWB, Fig. 2)"?

6. What was the problem with having thousands of different Drosophila lines each with a different enhancer driving the expression of inserted GAL4 genes?

a. How are we trying to avoid this same problem with our LexAop lines?

7. Other than misexpression studies, how is the GAL4/UAS bipartite system being used?

Lin and Potter: Editing Transgenic DNA Components by Inducible Gene Replacement in Drosophila

Lin and Potter Article

Transgenic *Drosophila* lines for LexA-dependent gene and growth regulation

G3 (Bethesda). 2022 Mar; 12(3): jkac018. Published online 2022 Jan 19. doi: 10.1093/g3journal/jkac018

Red Queen Article

Plucking Rubies from the Rubbish

Imaging

The Imaging Crosses

In this section, the crosses needed to explore the expression pattern of each novel LexA conversion are explained.

Remember that, with our substitution, LexA is in an enhancer trap P-element with a weak P-element promoter that is designed to trap expression patterns of enhancer(s) “nearby”. We have some information on Flybase about where these formerly GAL4 (now LexA) insertions are located and the documented expression patterns. We want to take images of the expression patterns of our LexA lines to compare those images with images of the GAL4 lines to see if they are the same.

To do so we must cross our LexA driver flies with LexAop responder flies to produce offspring flies that will make GFP but ONLY in the cells and at the times dictated by the trapped enhancer. Only where the LexA transcription factor is expressed can it bind to the LexAop site and turn on transcription of the GFP protein.

Below are explanations of three crosses to do after successfully establishing a stable stock for a converted line.

Cross to Observe the Expression Pattern of LexA

MALE: w/Y; P{w+, RFP+ LexA}/CyO; TM6B/ftz,e **X** **FEMALE:** w-; P{w+, LexAop2-mCD8::EGFP}

Offspring: w-; P{w+, RFP+ LexA}/+; P{w+,LexAop2-mCD8::EGFP}/+

The male is heterozygous for the converted LexA transgene in the above cross and so not all the progeny from the following cross will have the LexA transgene.

The female is homozygous for the GFP transgene and so all the progeny will have LexAop2-mCD8::EGFP.

How can you select the larval progeny (sorry no curly wing yet) with both transgenes from mom and dad?

If you have access to a fluorescence stereo microscope, then you can tell by looking for RFP+ expression in larval eyes and brains. This was initially a big selling point for the 3xP3-RFP transgene because you can genotype larvae!

So…from the above cross, select only RFP+ larval progeny to image.

Cross to Confirm that Gal4 is no Longer Produced

If you can afford it, you may also make a few ‘control’ crosses as well. The first control is to verify there is no longer functional GAL4 produced after you converted the line by cutting the Gal4 gene and inserting the LexA gene.

MALE: w/Y; P{w+, RFP+ LexA}/CyO; TM6B/ftz,e **X** **FEMALE:** w-; P{w+, UAS-mCD8::EGFP}

Offspring: w-; P{w+, RFP+ LexA}/+; P{w+,UAS-mCD8::EGFP}/+

For the reasons explained above, you should only dissect larva with RFP expression in brains and eyes.

The expectation is that you do not see any GFP signal in the tissues of these RFP+ progeny.

If you DO see some faint GFP, please record this information! This might be happening because of incomplete cleavage of 2A self-cleaving peptide (see the section titled - Use of CRISPR/HDR to Convert Gal4 Lines to LexA Lines and a Bit About the Gene Expression After the Conversion for more information on this issue).

Cross to Compare the Expression Pattern of Converted LexA to the Original Gal4

The second control is to compare the expression patterns of the original GAL4 and converted LexA.

MALE: w/Y; P{w+, GawB} **X** **FEMALE:** w-; P{w+, UAS-mCD8::EGFP}

Offspring: w-; P{w+, GawB}/+; P{w+,UAS-mCD8::EGFP}/+

The expectation is that you will see the same GFP expression patterns as you saw in the converted LexA with LexAop2-mCD8::EGFP.

If you see anything different, please document this carefully.

This might be a result of possible enhancer activities of 3xP3-RFP and y[+] transgenes in the converted LexA line, and the difference may go away after these marker transgenes are removed (see below).

If you mixed up the vials of this GAL4/UAS cross with the LexA/LexAop cross, don’t worry because RFP+ will tell you if this happened.

Larval Dissection

Larval Inversion. Your mantra is "Protect forcep tips!! Protect forcep tips!!" <u>NEVER remove the sheaths from forceps unless you are seated and your hands are resting on either side of the microscope. Move slowly. Once ruined your forcep tips will never be the same.</u> Ask me how I know….

Videos:

1. https://youtu.be/sTjLXYuvGkg
2. https://youtu.be/Y8py_dPMtao

<u>You'll need:</u> indentation plate(clean)

PBS

your forceps (two pairs)

Kimwipes left and right of the stereoscope.

Third instar larvae

Fixation solution in labeled tube

1. Use blunt forceps (aka "holding forceps") to choose stage 3 larvae (the largest ones) that have NOT started to pupate. You will need to collect 10-12 larvae per line (more if your dissection skills are not yet wonderful). **Use tape to label the well plate with the line number for your larvae. Also use a sharpie to label your vials with fix in them. Essential!!**
2. Place the larvae in a depression plate with PBS solution in the first 9 wells; put the larvae in the middle well and you should dissect in the corner wells.
3. Pull one larva at a time from the central well into a corner well with your blunt forceps. Now and only now should you remove the sheath from the sharp forceps tip.
4. Pinch the larva about 60% down the body toward the tail with the sharp forceps.
5. Place the blunt forceps beside the sharp forceps and pinch the body just in front of the sharp forceps - and then tear the larva in half but **do not release the torn body with your blunt forceps.**
6. Grab the mouth hooks with the sharp forceps (in line with the body).
7. Release your hold on the back end of the body with the blunt forceps and use them to push the body (inside out) up the sharp forceps as far as the body wall reaches. Do not tear the body wall. This process is a lot like turning a sock inside out… with the larval body wall acting as the sock.

8. Relax pressure on the fine forceps and re-compress a few times to loosen the inverted body on the forcep tips, then slide the inverted larva off the fine tips into a clean pool of PBS in the second row of wells.
9. When finished dissecting, place the inverted larvae in a vial with 4% formalin as specified below. (Start of antibody staining protocol)

Dissection of tissues post staining:

<u>You'll need:</u> indentation plate(clean)

a plain glass microscopy slide and 3 frosted slides (clean with alcohol)

a coverslip (yes, clean with alcohol)

PBS

Mounting media (DAPI)

your forceps (two pairs)

nail polish (Chanel Rouge Fatale or similar, clear nail polish works well, too) vial(s) with third instar larvae crawling up the wall

Kimwipes left and right of stereoscope.

Stained & inverted larval carcasses

Forceps are very sharp with sensitive tips. Handle with care. When not in use, place sleeve back on. Never store them or pass them to a person with tips pointing up.

1) Transfer using a pipet one inverted larva into an indentation slide filled with 1mL of PBS
2) If not done during inversion step, remove trachea from body wall
3) Dissect off fat bodies and place into droplet of mounting media on your plain slide or discard if they are non-fluorescent
4) Find the esophagus (follow the gut up to the proventriculus. Pull slightly. The esophagus links the ball of the proventriculus with the brain.). Grab esophagus & pull *without pulling on the heart which is parallel to the esophagus*. This is the fore-mid & hindgut. Place into mounting media if it is fluorescent
5) Find the wing/leg haltere discs. Dissect into mounting media if they glowed.
6) To dissect the brain/ring gland complex, cut the optical nerve connecting eye disc and optical lobe using a single prong of sharp forceps. Pluck off the extra discs under the VNC (Ventral

Nerve Cord). Slide a single prong under the VNC to cut extruding axons. Forklift CNS/VNC out & into mounting medium. Collect eye and antenna disks and place in mounting medium.

7) Move tissue samples from the plain glass slide to the final slide and place them in the DAPI, arranging them so that they are fully extended for max. visibility.

8) Place a piece of clean cuticle (cuticle with all disks removed and no mouthhooks) at the corners where your coverslip will go or use doubled tape squares to keep the coverslip from crushing the tissues.

9) Place coverslip on top of mounting media by placing one side flat on the slide and slowly lowering it down to avoid bubbles. Add DAPI as needed to exclude bubbles.

10) Seal with nail polish at the 4 corners and, once dry, all along the edges.

11) Store in a dark & cool place horizontally until the nail polish dries completely.

12) Record expression pattern at microscopes downstairs and complete your notes on what tissues express GFP. VERY IMPORTANT!!!

D. look carefully under highest power (150x) on the stereomicroscope for fluorescence in

a. the central nervous system (CNS) and ventral nerve cord (VNC)

b. the fat bodies (large whitish cell masses with cells with visible nuclei)

c. the digestive system

d. the disks and muscles along the body wall

e. take images if you see anything interesting

E. if you see fluorescence then switch to the Axiophot and photograph the fluorescent area(s) under 400x.

Using PCR and Sequencing to Confirm HACK - Concept

The first image is an example of a portion of the genome from the original Gal4 lines - these are the flies ordered from the Bloomington Stock Center. The second image is a region of the genome from the second chromosome from our F_0 female - our HACK flies. The last image is this same region of the genome in a converted fly.

There are four primers in these images - all primers do not anneal in all three genomes. The primer annealing locations are marked in parentheses. The vertical line which labels the primer marks the 5’ end of the primer.

We will use four combinations of primers (1 and 4, 3 and 4, 1 and 2, 3 and 2) on each original Gal4 line, the HACK line and each converted stock. In each case the forward or left primer is listed first and the reverse or right primer is listed second.

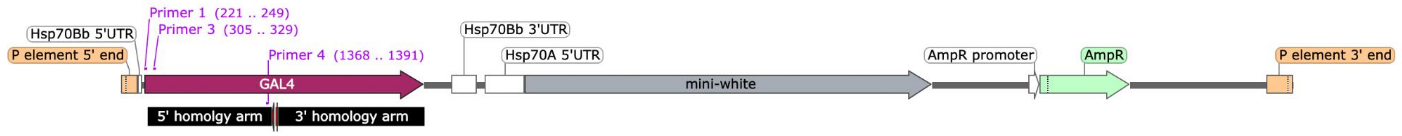

P{GawB}
11,067 bp

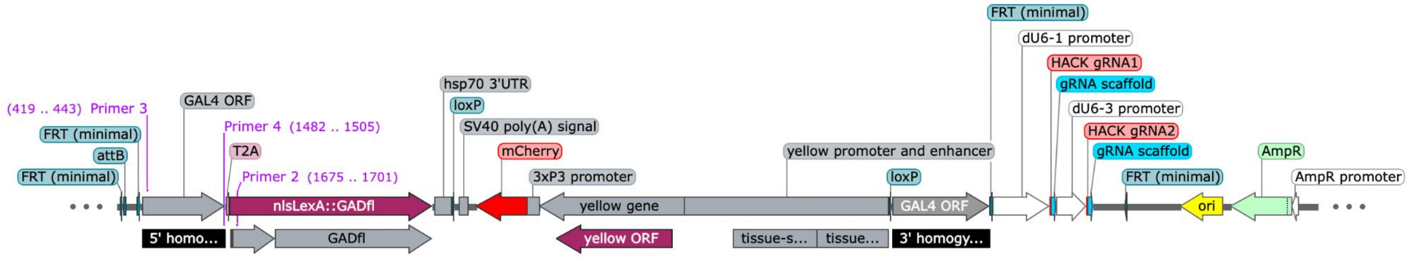

pHACKy-GAL4-nlsLexA::GADfl
16,891 bp

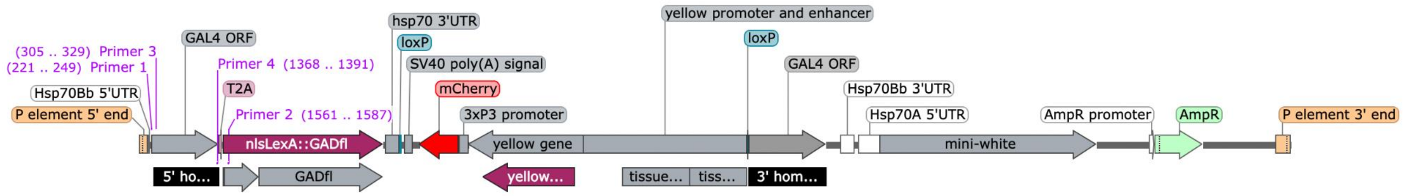

P{ET-lexA::GAD.GB}
20,375 bp

Here are two additional pictures - these show sequence level detail for the primer annealing sites. Both of these were taken from the converted fly sequence.

Picture 1 -

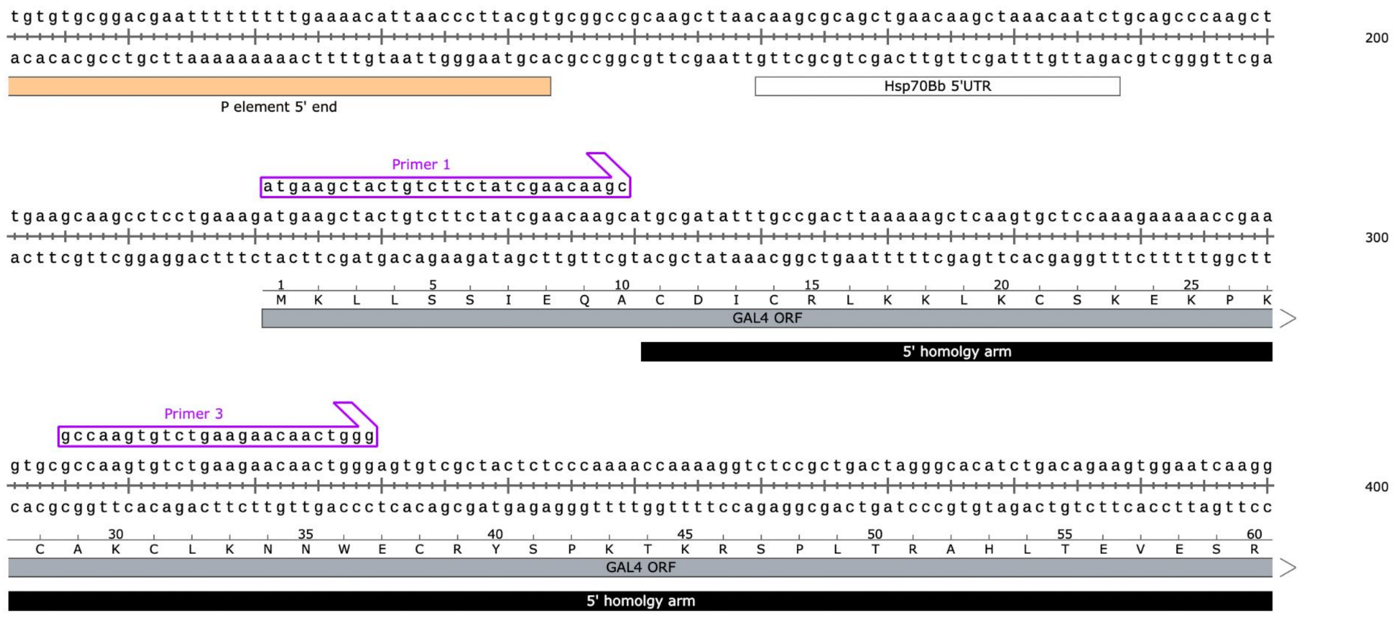

Picture 2 -

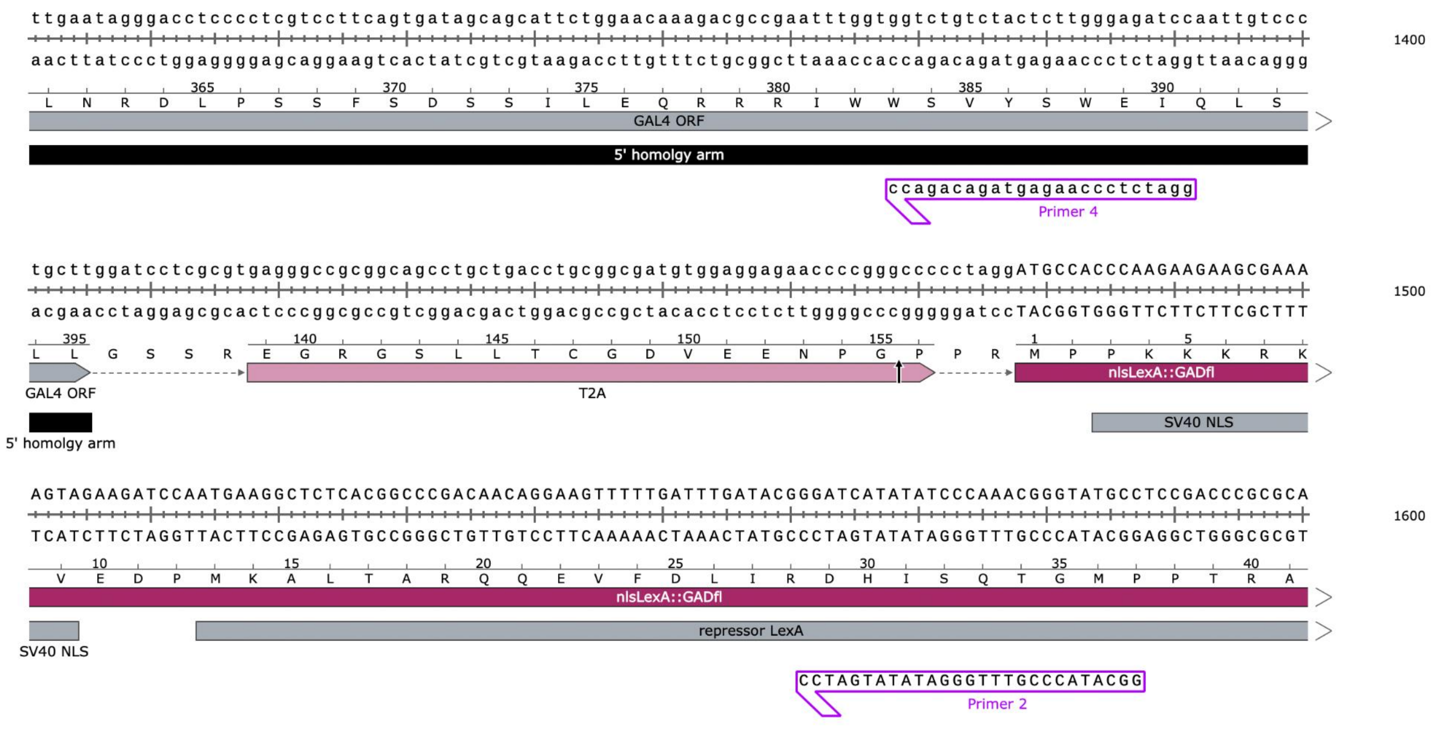

Fill in this table to organize what you know about these primers…

Pimer	Left/forward OR Right/reverse Primer?	Location (options are GAL4 ORF - not part of 5' homology arm, GAL4 ORF - part of 5' homology arm, 5' end of LexA)
1		
2		
3		
4		

Fill in the table below to predict our results when we run our PCR products on a gel. In addition to noting whether we expect a band, also record the expected size.

	1 and 4	3 and 4	1 and 2	3 and 2
Gal4<br>Original stock ordered from Bloomington Stock Center<br><br>Male in F_0 cross				
HACK<br>Carries the sequence to be used in HDR<br><br>Female in F_0 cross				
Converted<br>After successful CRISPR/HDR<br><br>Desired male offspring from F_1 cross and used as the male parent in F_2 cross				

We will sequence one of the bands from the converted line to confirm that the sequence matches what would be expected if CRISPR/HDR has inserted the donor DNA as expected. Which amplicon would you choose to sequence and which primer would you use as a sequencing primer? Explain your thinking.

Using PCR and Sequencing to Confirm HACK - Protocol

Fly Collection

- Please collect five to ten flies per tube.

- Please collect red eyed flies from the cross you are using to establish your stable stock (these would be siblings to the flies you are putting in your stable stock vial).

- Enter the **original Gal4 stock number and the stock identifying letter** in column C of the spreadsheet corresponding to each tube number in column B. (you will see that our collection tube numbers are not sequential because each sample will be used to generate 4 separate PCR samples).

Protocol for DNA Extraction

(entire booklet, here is a video to help you visualize what you will be doing and here is another, insect specific protocol)

1. Homogenize sample using clean pestle
2. Add 180 microliters of PBS
3. Homogenize sample using clean pestle
4. Add 20 microliters of proteinase K
5. Add 200 microliters of buffer AL (without ethanol)
6. Vortex thoroughly
7. Incubate at 56 degrees C for 10 minutes
8. While waiting - number tops of spin columns and place in collection tubes
9. Add 200 microliters ethanol (96 to 100%)
10. Vortex thoroughly
11. Centrifuge at 14,000 rpm for 1 minute
12. Pipette all of liquid mixture (leave the chunks behind) into spin column of the same number
13. Centrifuge at 8,000 rpm for 1 minute
14. Discard collection tube and flow-through
15. Place spin columns in new collection tubes
16. Add 500 microliters of Buffer AW1
17. Centrifuge at 8,000 rpm for 1 minute
18. Discard collection tube and flow-through
19. Place spin columns in new collection tubes
20. Add 500 microliters of Buffer AW2
21. Centrifuge at 14,000 rpm for 3 minutes
22. Discard flow-through and place spin column back in the same collection tube
23. Centrifuge at 14,000 rpm for 1 minute
24. Label 1.5 ml tubes with appropriate numbers
25. Place spin column in tube with matching number
26. Add 100 microliters of Buffer AE - place directly on the membrane
27. Let sit for at least 1 minute
28. Centrifuge at 8,000 rpm for 1 minute
29. Discard spin columns
30. Eluted nucleic acids are now in 1.5 ml tube
31. Store in refrigerator until next step

PCR

(watch videos at the bottom of this page to learn more about Q5)

1. Label PCR tubes
2. Make master mix for each DNA prep

reagent	Per sample	(Number of samples) +1	Total volume
water	7 µl	X 5	35 µl
Q5 Hot Start High-Fidelity 2X Master Mix	10 µl		50 µl
single DNA prep	1 µl		5 µl

3. Vortex and centrifuge each master mix
4. Aliquot 18 µl master mix into each tube
5. Add 1 µl of forward primer (1 or 3) (10 µM)
6. Add 1 µl of reverse primer (2 or 4) (10 µM)
7. Centrifuge
8. Perform PCR
    a. 98°C 2 min
        i. 98°C 10 sec
        ii. 60°C 20 sec
        iii. 72°C 60 sec
        iv. x35
    b. 72°C 5 min

c. hold at 4°C

Primers (updated on 4-28-2023 based on SnapGene files)

primer 1: atgaagctactgtcttctatcgaacaagc
primer 2: ggcatacccgtttgggatatatgatcc
primer 3: gccaagtgtctgaagaacaactggg
primer 4: ggatctcccaagagtagacagacc

Gels

1. Label a new set of PCR tubes
2. Add 1.5 µl of DNA loading dye to each tube
3. Add 10 µl from each PCR tube into new tube with corresponding label
4. Run the samples in 1% agarose gel/TBE using lane assignments from spreadsheet
5. Run 5 microliters of 100 bp ladder in lanes 1 and 10
6. Run at 100V for 60 minutes

PCR Clean Up

This protocol is to be done with the remaining 10 μl of PCR sample. We are only going to clean the samples we intend to sequence.

1. Add 5 volumes of PB to 1 volume of PCR sample
2. Number spin columns and place in collection tubes
3. Pipette all of sample into spin column
4. Centrifuge at 13,000 rpm for 1 minute
5. Discard flow-through and place spin column back in same collection tube
6. Add 750 microliters of Buffer PE
7. Centrifuge at 13,000 rpm for 1 minute
8. Discard flow-through and place spin column back in same collection tube
9. Centrifuge at 13,000 rpm for 1 minute
10. Label 1.5 ml tubes with appropriate numbers
11. Place spin column in tube with matching number
12. Add 30 microliters of Buffer EB - place directly on the membrane
13. Let sit for at least 1 minute
14. Centrifuge at 13,000 rpm for 1 minute
15. Discard spin columns
16. Eluted nucleic acids are now in 1.5 ml tube

Quantifying DNA Concentration

The next step is to quantify the concentration of DNA present in the PCR reaction after purification with a Qiagen kit. We are doing this step in order to use the optimal amount of DNA in the sequencing reaction.

A few suggestions for using the nanospec:

We need to blank the nanospec every 30 minutes. The purpose of blanking is to set the absorption of the liquid our sample is dissolved in to zero. It is kind of the same idea as taring a balance. The nanospec will prompt you to measure the blank two times before allowing you to measure your samples.

Wipe the top and bottom of the sample area gently with a kimwipe after every sample.

Use 1-2 µl of sample for a measurement.

Close the sample arm GENTLY.

Wipe the top and bottom of the sample area with DI water at the end of a session.

Record the ng/µl portion of the display in the spreadsheet. This is the concentration of nucleic acids.

Stock Solutions

We are going to make stock solutions with a concentration of 4 ng/µl. To do this we will use our cleaned PCR product and add the appropriate amount of molecular grade water to yield our desired final concentration. To do these calculations use the formula:

$C_1V_1=C_2V_2$

Where C stands for concentration and V stands for volume and the one stands for initial and the two for final.

We will make our stock solutions in autoclaved 1.5 ml tubes. Please label the tubes with the appropriate number and an S for stock solution.

Sequencing Reactions

These reactions need to be sent to the company in autoclaved 8 tube strips. Each tube must be labeled top and side with the initials CR and sequencing tube number (example: CR01, CR02…). The tubes must be kept connected and must be in numerical order.

- Label your tubes on the TOP and SIDE with the labels in column O on the spreadsheet
- Add 5 microliters of primer 2 (diluted to 5 pmol/microliter) to each tube
- Add 10 microliters of the correct stock solution to each tube

I. Pre-Mixed

In the **same tube**, mix template (10 µl) and your primer (5 µl) according to the table below. To use a GENEWIZ Universal Primer, simply submit template at the requested concentration in 10 µl. See the Technical Notes Tab for Tips on how to purify PCR products.

DNA Type	DNA Length (include vector)	Template Concentration in 10 µl	Template Total Mass	Your Primer Total Picomoles	Premixed Volume* (Template + Your Primer)
Plasmids	<6 kb	~50 ng / µl	~500 ng	25 pmol	15 µl
	6 - 10 kb	~80 ng / µl	~800 ng		
	> 10 kb	~100 ng / µl	~1000 ng		
Purified PCR Products	<500 bp	~1 ng / µl	~10 ng	25 pmol	15 µl
	500 - 1000 bp	~2 ng / µl	~20 ng		
	1000 - 2000 bp	~4 ng / µl	~40 ng		
	2000 - 4000 bp	~6 ng / µl	~60 ng		
	>4000 bp	Treat as plasmid	Treat as plasmid		

What happens to the stocks after they are stabilized but before they can be used by other researchers?

Remove Marker Transgenes from Stock

To visually identify rare progeny from the HACK events, we used marker transgenes (3xP3-RFP and y[+]) which were useful during screening but now you may want to remove these markers from our payload transgene (T2A-nlsLexA::GAD) for a few reasons.

1. Many imaging techniques use different fluorophores to color-label cells and you might need the red channel for your signal detection. If you remove these marker transgenes, scientists could use the red channel for imaging and also could follow another transgene that carries y[+] marker if needed. Lin and Potter deposited their stocks with RFP+ still in them, but we want to make our stocks more user friendly.

2. We want to make the enhancer activity of the converted line as close as possible to the original. However, marker transgenes come with their own enhancers (3xP3 and yellow enhancer in our case) which might drive LexA expression in other unintended cells that the original GAL4 did not drive in. By removing these markers, we could avoid this possibility.

How do scientists 'efficiently' remove transgenes that you do not need any more from the genome? You could use CRISPR/Cas9 again to create two double strand breaks just outside of our marker transgenes, and let the NHEJ to join the ends without the markers, but this process is unpredictable and not efficient. We use an efficient way called site-specific recombination. For example, Cre recombinase proteins recognize and recombine two *loxP* DNA sequences, thus 'excising' out any intervening DNA sequence between two *loxP* sequences (https://blog.addgene.org/plasmids-101-cre-lox). For an efficient removal of our marker transgenes after identification, we designed our HACK donor with two *loxP* sequences just outside of our marker transgenes (highlighted in blue) and these sequences are also in your converted LexA line after HDR.

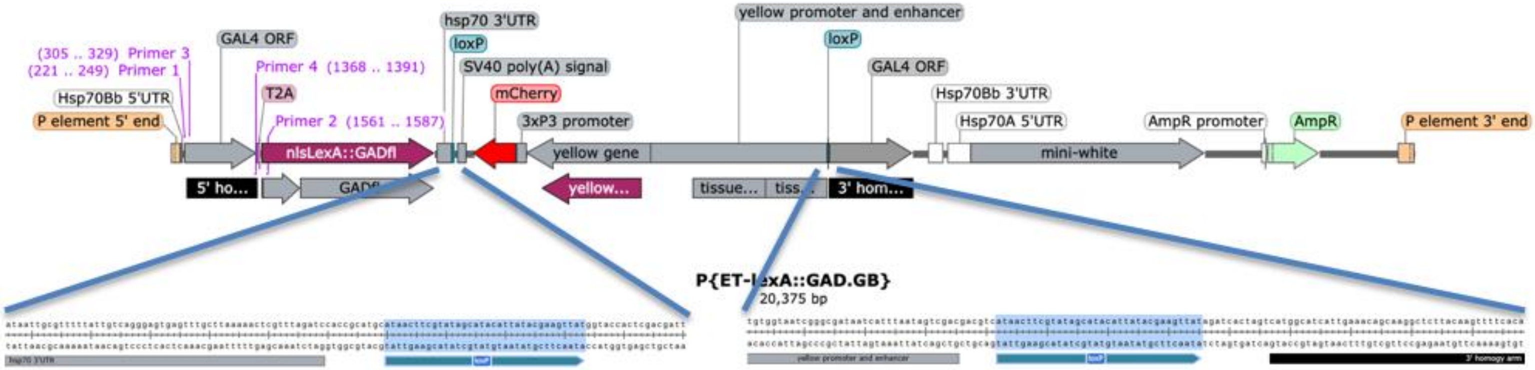
(305 .. 329) Primer 3
(221 .. 249) Primer 1
Hsp70Bb 5'UTR
P element 5' end
GAL4 ORF
Primer 4 (1368 .. 1391)
T2A
Primer 2 (1561 .. 1587)
nlsLexA::GADfl
5' ho...
GADfl
hsp70 3'UTR
loxP
SV40 poly(A) signal
mCherry
3xP3 promoter
yellow gene
yellow...
yellow promoter and enhancer
loxP
GAL4 ORF
tissue...
tiss...
3' hom...
Hsp70Bb 3'UTR
Hsp70A 5'UTR
mini-white
AmpR promoter
AmpR
P element 3' end
P{ET-lexA::GAD.GB}
20,375 bp

Deletion

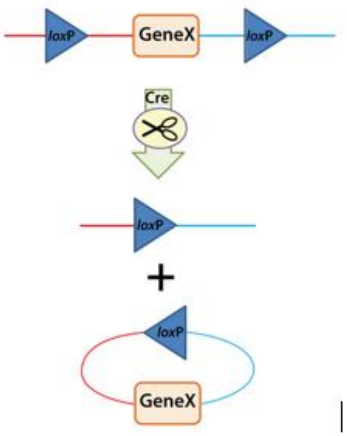
loxP
GeneX
loxP
Cre
loxP
+
loxP
GeneX

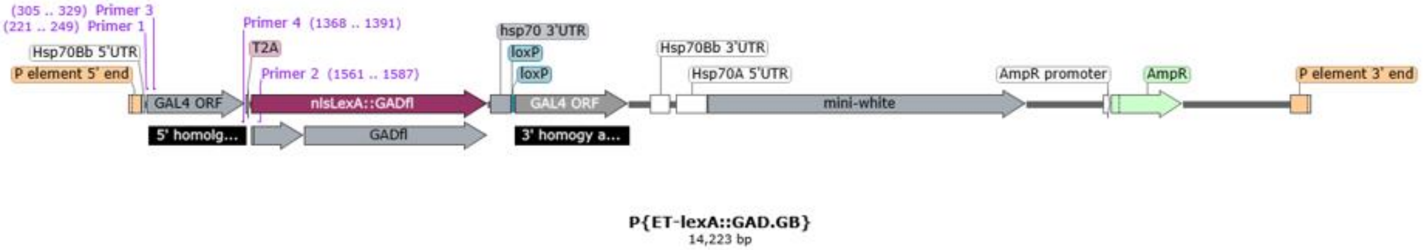
(305 .. 329) Primer 3
(221 .. 249) Primer 1
Hsp70Bb 5'UTR
P element 5' end
GAL4 ORF
Primer 4 (1368 .. 1391)
T2A
Primer 2 (1561 .. 1587)
nlsLexA::GADfl
5' homolg...
GADfl
hsp70 3'UTR
loxP
loxP
GAL4 ORF
3' homogy a...
Hsp70Bb 3'UTR
Hsp70A 5'UTR
mini-white
AmpR promoter
AmpR
P element 3' end
P{ET-lexA::GAD.GB}
14,223 bp

When your partner scientists receive your stable stocks, they will cross your lines to another transgenic line carrying P{Crey} which expresses the Cre recombinase in germlines. In F3 male germlines, Cre proteins recognize loxP-3xP3-RFP-y[+]-loxP sequence (also called '*loxP* cassette') in your converted LexA.G4H sequence, and remove the cassette by recombining two loxP sequences.

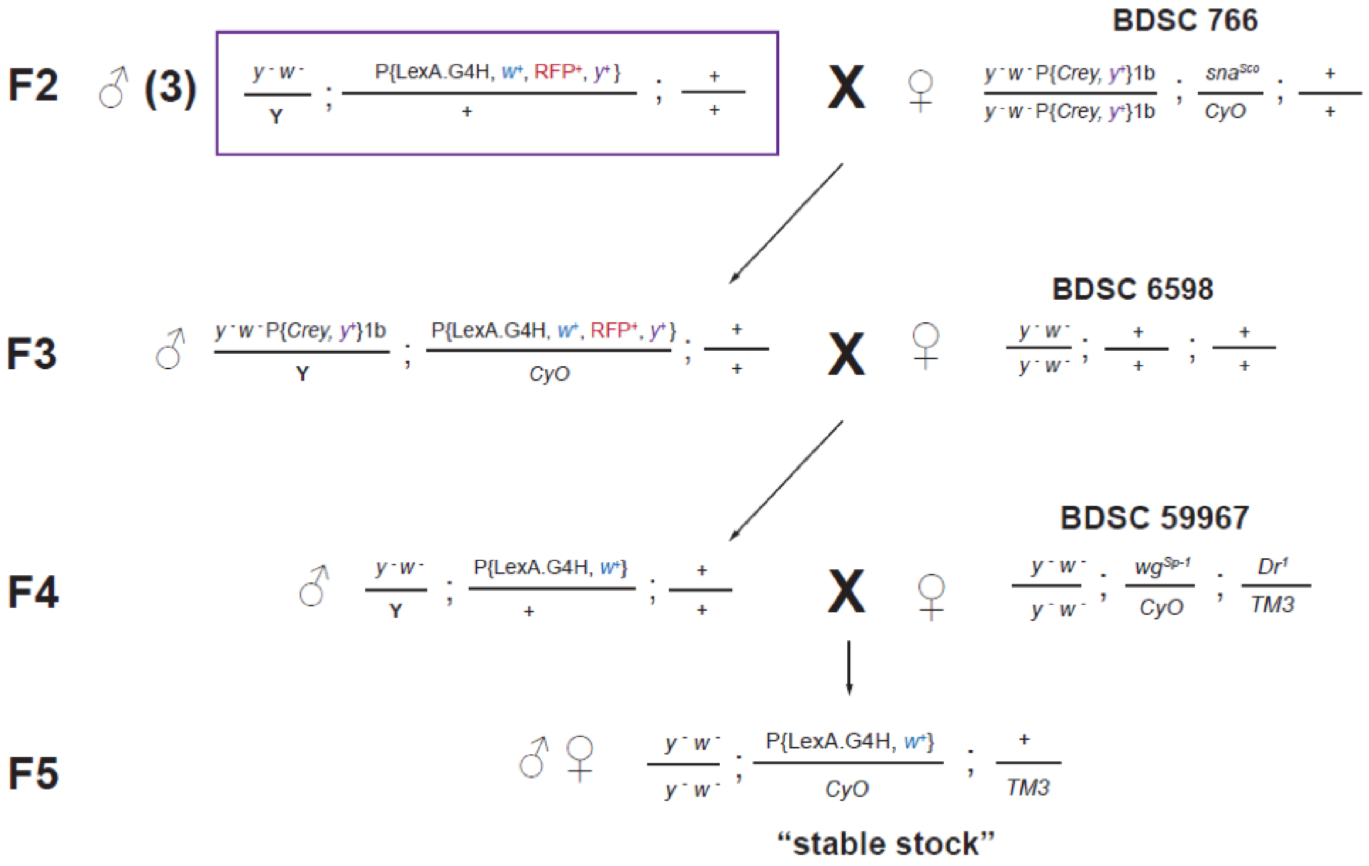

The cassette-removed LexA.G4H male progeny (F4) can be identified by their yellow body and mini-white but without RFP expression. Compared to your HACK, this site-specific recombination is so efficient, all F4 male progeny with mini-white are usually with yellow body color. A single male carrying cassette-removed LexA.G4H transgene (now only marked by mini-white) will be balanced again to establish a 'stable' stock. After confirming expression patterns of the cassette-removed LexA.G4H stable stock using LexAop2-mCD8::GFP line, the final 'stable' stock will be deposited in BDSC. The images of GAL4 and converted LexA.G4 expression patterns will be published somewhere as documentation of 'identical' or 'near identical' enhancer activities of YOUR converted LexA.G4H line!

Enter stocks in Bloomington Stock Center

The stocks are added to the collection at the Bloomington Stock Center - a repository for fruit flies for the research world. Read this New York Times article (Giaimo, Cara. New York Times, Late Edition (East Coast); New York, N.Y. [New York, N.Y]. 15 Dec 2020) for a description of the place.

Fruit Flies and the People Who Care for Them: [Science Desk]

Giaimo, Cara

ProQuest document link

FULL TEXT

The rooms that make up the Bloomington Drosophila Stock Center at Indiana University are lined wall to wall with identical shelves. Each shelf is filled with uniform racks, and each rack with indistinguishable glass vials.

The tens of thousands of fruit fly types within the vials, though, are each magnificently different. Some have eyes that fluoresce pink. Some jump when you shine a red light on them. Some have short bodies and iridescent curly wings, and look "like little ballerinas," said Carol Sylvester, who helps care for them. Each variety doubles as a unique research tool, and it has taken decades to introduce the traits that make them useful. If left unattended, the flies would die in a matter of weeks, marooning entire scientific disciplines.

Throughout the Covid-19 pandemic, workers across industries have held the world together, taking on great personal risk to care for sick patients, maintain supply chains and keep people fed. But other essential jobs are less well-known. At the Stock Center dozens of employees have come to work each day, through a lockdown and afterward, to minister to the flies that underpin scientific research.

Tiny Bug, Huge Impact

To most casual observers, fruit flies are little dots with wings that hang out near old bananas. But over the course of the last century, researchers have turned the insect -- known to science as Drosophila melanogaster -- into a sort of genetic switchboard. Biologists regularly develop new "strains" of flies, in which particular genes are turned on or off. Studying these slight mutants can reveal how those genes function -- including in humans, because we share over half of our genes with Drosophila. For instance, researchers discovered what is now called the hippo gene -- which helps regulate organ size in both fruit flies and vertebrates -- after flies with a defect in it grew up to be unusually large and wrinkly. Further work with the gene has indicated that such defects may contribute to the unchecked cell growth that leads to cancer in people.

Other work with the flies has shed light on diseases from Alzheimer's to Zika, taught scientists about decision-making and circadian rhythms and helped researchers using them to win six Nobel Prizes. Over a century of tweaking fruit flies and cataloging the results has made Drosophila the most well-characterized animal model we have.

It's a big role for an unassuming bug. "When I try and tell people what I do, the first thing they usually say is, 'Why would you keep fruit flies alive? I try and kill them!'" said Ms. Sylvester, who has been a stockkeeper at Bloomington since 2014.

If a few hitchhike to her house from the grocery store, her kids razz her, she added: "'Mom, you brought your co-workers home from work again.'"

The Bloomington Drosophila Stock Center is the only institution of its kind in the United States, and the largest in the world. It currently houses over 77,000 different fruit fly strains, most of which are in high demand. In 2019, the center shipped 204,672 vials of flies to labs in 49 states and 54 countries, said Annette Parks, one of the center's five principal investigators.

It is "one of the jewels we have in the community," said Pamela Geyer, a stem cell biologist at the University of Iowa who has been ordering flies from the stock center for 30 years.

Other model organisms can be frozen at particular life stages for long-term storage; lab freezers the world over hold

mouse embryos and E. coli cultures. But fruit flies can't go on ice. Caring for the creatures means regularly "flipping" them: transferring them from an old vial to a clean one that has been provisioned with a dollop of food. Quarantined with other members of their strain, the flies mate and lay eggs, which hatch, pupate and reproduce, continuing the cycle.

"We have strains in our collection that have been continuously propagated like that since around 1909," across generations and institutions, said Cale Whitworth, another stock center principal investigator. To keep their millions of Drosophila flipped and happy, the center employs 64 stockkeepers, as well as one media preparator -- think fly-food cook -- as well as a kitchen assistant and five dishwashing personnel.

Don't Flip Out

At the stock center, as everywhere, the pandemic's first stirrings felt ominous. "I remember joking with people, 'We're the people in the beginning of the dystopian novel, and we don't know what's coming yet,'" Ms. Sylvester said.

As case numbers rose, Dr. Whitworth packed a go-bag with a pillow and a toothbrush, imagining the worst. "I was in the full-on, 'Everyone's sick, last man on Earth' type thing," he said. "Like, 'How many flies can I flip in a 20-hour period, sleep for four hours, and keep flipping the next day?'"

Instead, when Indiana University shut down on March 15, the stock center stayed open.

Kevin Gabbard, the fly-food chef, did an emergency shop. Although they eat the same thing every day -- a yeasty mash of mostly corn-based products -- flies can be picky. Mr. Gabbard, risking nothing, ordered two months' worth of their preferred brands. "You think cornmeal's cornmeal," he said. "But it's not if it's not right."

The co-directors developed a more robust Hail Mary plan that, if absolutely necessary, would allow them to "keep most of the flies alive with just eight people," said Dr. Whitworth. They also decided to halt all shipments, focusing their energy on fly care.

On March 26, flies stopped leaving the building -- and almost immediately, supportive messages began rolling in. "You are all amazing," read one email. "The fly community is strong because of the phenomenal work that you do."

Around the same time, the employees had a choice to make. Deemed essential workers, they were authorized to come to campus. The university guaranteed them full pay even if they decided to stay home, or time-and-a-half for coming in. (The center covers its costs through a combination of federal National Institutes of Health grants and its own earnings from fly sales.)

The vast majority chose to continue working, said Dr. Whitworth -- even though the job was suddenly quite different. The center is usually a very social workplace, with birthday parties and group lunches. Hours are normally flexible, a big selling point for employees, many of whom are parents or students, or have retired from full-time work.

Now people work in masks, often in separate rooms. Shifts in one of the center's buildings became strictly scheduled to avoid overlap. "You can be working alone for quite a while, maybe all day," said Roxy Bertsch, who has been a stockkeeper since 2018.

And for the first several weeks, the stockkeepers -- many of whom perform additional duties, such as packing, shipping and training -- spent all their time flipping flies, which is monotonous and hard on the hands. "All we were doing was coming in, feeding flies and leaving," Mrs. Bertsch said.

But she kept going back. After her son was potentially exposed to the coronavirus, and she had to self-quarantine, she counted down the 14 days until she could return.

"There is no way you are keeping me from work if I could be here," she said.

Ms. Sylvester specializes in caring for flies whose mutations mean they need extra TLC. She also worked full-time throughout the shutdown, buoyed by concern for her charges. "I mostly just love the flies and don't want them to die," she said. "I never thought I would love larvae so much."

Getting Back to Ship-Shape

In mid-May, the center began shipping stocks again. Dr. Parks passed along another batch of messages, many of them now tinged with relief.

"Feels like Christmas," tweeted a lab at Denmarks' Aarhus University, with a photo of a box of vials.

One message earlier in the spring from Tony Parkes, a biologist at Nipissing University in Ontario, had extolled all of

those "who go about their work with few accolades, but on whom everyone counts as a foundational backbone."

When Dr. Parkes's lab paused, he spent some of his unexpected down time thinking about the stock center. It is an equalizer, he said, enabling even small labs to tackle big questions "without requiring vast resources."

It also allows researchers to literally share their discoveries with each other. "You don't have to maintain your own library to have access to all of that information," he said, because the stock center is "there whenever you wish."

The people who keep the center running think about this, too. "It means a lot to know that you're a part of that," said Mrs. Bertsch.

But it adds some pressure. "We all feel this big weight to make sure the stock center is there for everyone," said Dr. Whitworth.

The pandemic continues, of course, and more obstacles loom. Although the fall semester passed without incident, cases are rising in the area, increasing the potential for another shutdown. Mail delays, both domestic and overseas, have prompted the center to suggest that their customers turn to private carriers -- flies perish if kept in transit too long.

Although they are no longer being paid extra, everyone keeps coming to work. And even if things take a turn, Dr. Whitworth is ready. "I never unpacked my bag," he said. "It's still sitting in the closet."

Photograph

"Flipped" vials that had contained fruit flies in the Bloomington Drosophila Stock Center at Indiana University. (PHOTOGRAPH BY KAITI SULLIVAN FOR THE NEW YORK TIMES) (D1); Right, Roxy Bertsch. Below, Cale Whitworth. Middle row from left: Margaret Elkins, a stockkeeper; a fruit fly; fly food recipes on the walls. Bottom row from left: fruit fly stocks; White 1, a stock that is the oldest in the collection. (PHOTOGRAPHS BY KAITI SULLIVAN FOR THE NEW YORK TIMES; BOB GIBBONS/ALAMY) (D3)
