# Supplementary figures and images for: Simplified homology-assisted CRISPR for gene editing in Drosophila
Source: G3 (Bethesda). 2023 Dec 7;14(2):jkad277. doi: 10.1093/g3journal/jkad277 (PMC10849607; doi:10.1093/g3journal/jkad277)

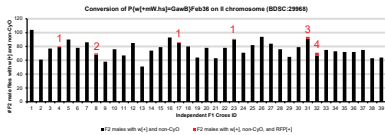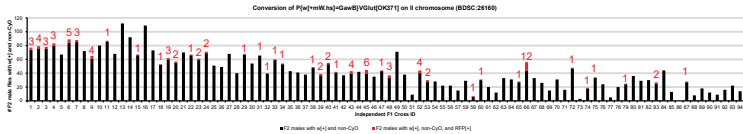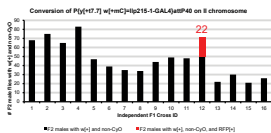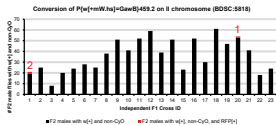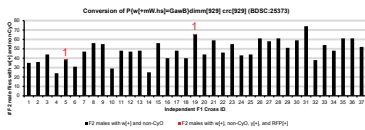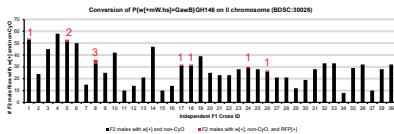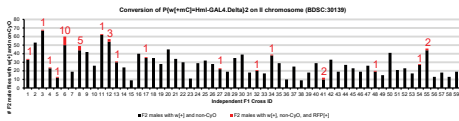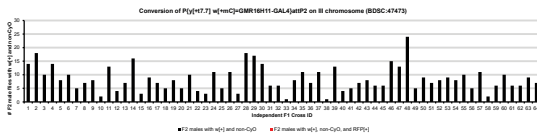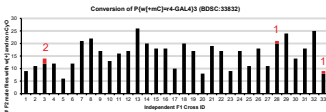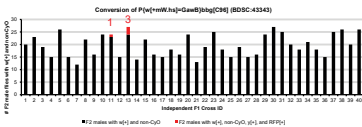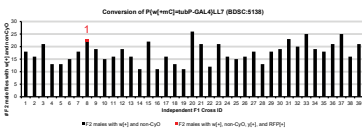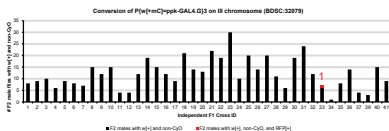

Supplement: jkad277_Supplementary_Data [file jkad277_supplementary_data.zip › Figure_S1_G3-2023-404519.pdf]

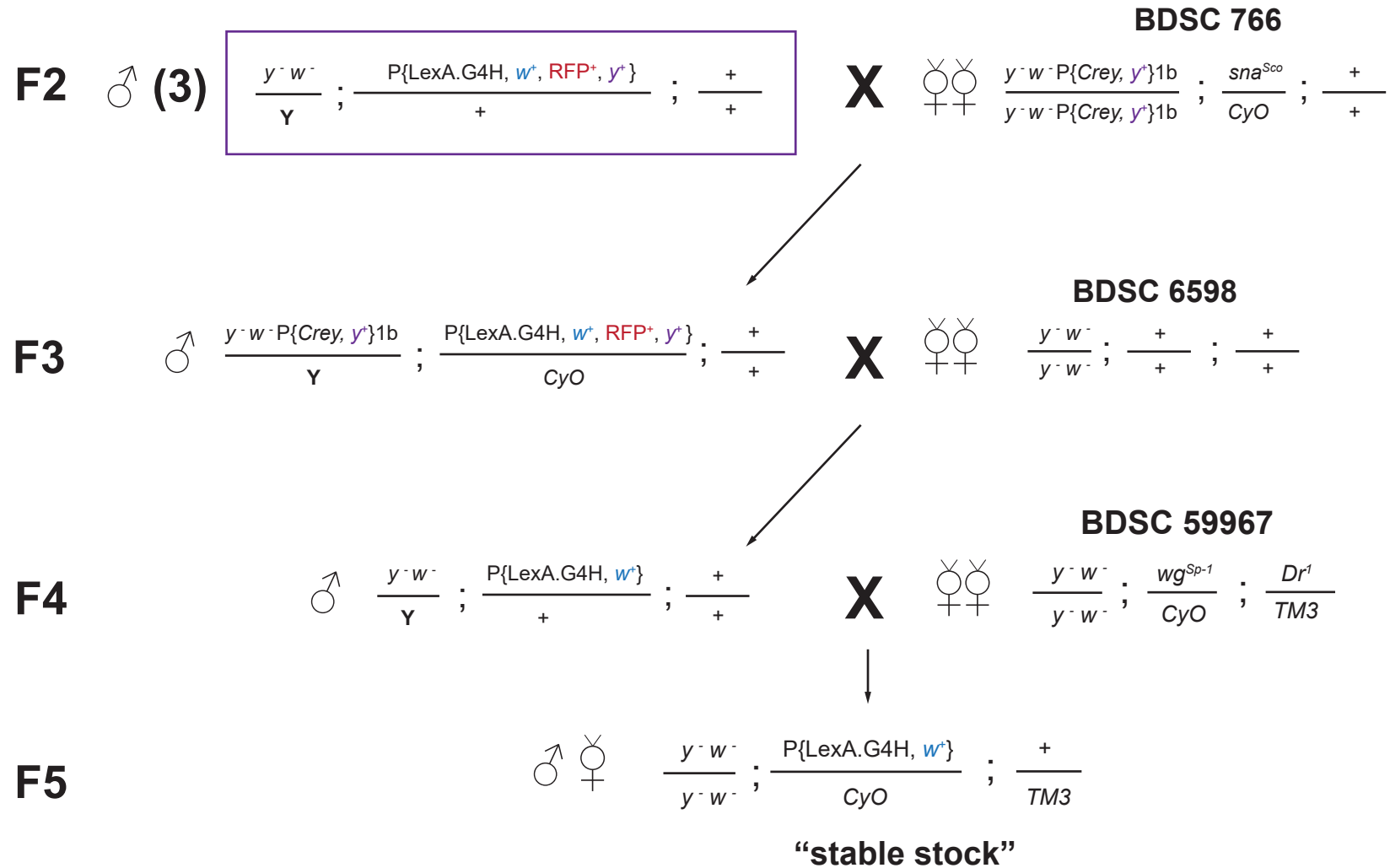

Supplement: jkad277_Supplementary_Data [file jkad277_supplementary_data.zip › Figure_S2_G3-2023-404519.pdf]
